# Josephin domain containing 2 (JOSD2) promotes lung cancer by inhibiting LKB1 (Liver kinase B1) activity

**DOI:** 10.1038/s41392-023-01706-y

**Published:** 2024-01-05

**Authors:** Tao Yuan, Chenming Zeng, Jiawei Liu, Chenxi Zhao, Fujing Ge, Yuekang Li, Meijia Qian, Jiamin Du, Weihua Wang, Yonghao Li, Yue Liu, Xiaoyang Dai, Jianya Zhou, Xueqin Chen, Shenglin Ma, Hong Zhu, Qiaojun He, Bo Yang

**Affiliations:** 1 Institute of Pharmacology & Toxicology, Zhejiang Province Key Laboratory of Anti-Cancer Drug Research, College of Pharmaceutical Sciences, Zhejiang University, Hangzhou, 310058 China; 2 Innovation Institute for Artificial Intelligence in Medicine, Zhejiang University, Hangzhou, 311199 China; 3 Ministry of Education Key Laboratory of Chinese Medicinal Plants Resource from Lingnan, Research Center of Medicinal Plants Resource Science and Engineering, Guangzhou University of Chinese Medicine, Guangzhou, 510006 China; 4 Center for Drug Safety Evaluation and Research of Zhejiang University, Hangzhou, 310058 China; 5 Department of Respiratory Disease, Thoracic Disease Center, The First Affiliated Hospital, College of Medicine, Zhejiang University, Hangzhou, 310006 China; 6 Department of Oncology, Hangzhou Cancer Hospital, Hangzhou, 310002 China; 7 Cancer Center of Zhejiang University, Hangzhou, China; 8 Engineering Research Center of Innovative Anticancer Drugs, Ministry of Education, Hangzhou, China

**Keywords:** Lung cancer, Cancer therapy

## Abstract

Non-small cell lung cancer (NSCLC) ranks as one of the leading causes of cancer-related deaths worldwide. Despite the prominence and effectiveness of kinase-target therapies in NSCLC treatment, these drugs are suitable for and beneficial to a mere ~30% of NSCLC patients. Consequently, the need for novel strategies addressing NSCLC remains pressing. Deubiquitinases (DUBs), a group of diverse enzymes with well-defined catalytic sites that are frequently overactivated in cancers and associated with tumorigenesis and regarded as promising therapeutic targets. Nevertheless, the mechanisms by which DUBs promote NSCLC remain poorly understood. Through a global analysis of the 97 DUBs’ contribution to NSCLC survival possibilities using The Cancer Genome Atlas (TCGA) database, we found that high expression of *Josephin Domain-containing protein 2 (JOSD2)* predicted the poor prognosis of patients. Depletion of JOSD2 significantly impeded NSCLC growth in both cell/patient-derived xenografts in vivo. Mechanically, we found that JOSD2 restricts the kinase activity of LKB1, an important tumor suppressor generally inactivated in NSCLC, by removing K6-linked polyubiquitination, an action vital for maintaining the integrity of the LKB1-STRAD-MO25 complex. Notably, we identified the first small-molecule inhibitor of JOSD2, and observed that its pharmacological inhibition significantly arrested NSCLC proliferation in vitro/in vivo. Our findings highlight the vital role of JOSD2 in hindering LKB1 activity, underscoring the therapeutic potential of targeting JOSD2 in NSCLC, especially in those with inactivated LKB1, and presenting its inhibitors as a promising strategy for NSCLC treatment.

## Introduction

Non-small cell lung cancer (NSCLC), which accounts for approximately 85% of lung cancer, is one of the leading causes of cancer death worldwide.^1^ Kinase-targeted therapies such as EGFR, ALK and MET inhibitors are commonly used to treat NSCLC patients.^2^ However, only ~30% NSCLC patients harbor the activating mutations in these kinases hence are responsive to these therapies.^3^ Therefore, further understanding of NSCLC tumorigenesis is in urgent need for developing more effective therapeutic treatments.

The deubiquitinases (DUBs), a group of diverse enzymes with well-defined catalytic sites that are frequently overactivated in cancers, have attracted much attention recently as potential therapeutic targets. For instance, OTUD6B, USP7 and COPS5, frequently found overexpressed in breast cancers, have been regarded as promising therapeutic targets.^4,5^ Nevertheless, the underlying mechanisms of DUBs in promoting NSCLC tumorigenesis remain poorly understood, which has hindered the discovery of DUBs inhibitors for NSCLC treatment. This warrants thorough investigation on identifying DUBs with important oncogenic roles in NSCLC and further elucidate their tumorigenic mechanisms.

In our analysis, Josephin Domain-containing protein 2 (JOSD2) stood out as its high expression was significantly correlated with poor prognosis of NSCLC patients and depletion of JOSD2 posed most potent inhibitory effects on NSCLC cell growth. JOSD2 belongs to the Machado-Josephin Domain-containing proteases (MJD) subfamily of DUBs along with other three cysteine-proteases, Ataxin-3, Ataxin-3L and JOSD1, which share a common catalytic Josephin domain.^6^ A recent study suggested that JOSD2 might function as a DUB to stabilize PHGDH protein level and PHGDH defines a metabolic subtype of lung adenocarcinomas with poor prognosis.^7^ In addition, Krassikova et al. also demonstrated that JOSD2 promoted glycolysis in tumor cells through deubiquitination and stabilization of three important enzymes involved in glucose metabolism, namely PHGDH, PFK-1 and Aldolase A.^8^ However, the biological function and underlying mechanism of JOSD2 in lung cancer are poorly understood yet. Therefore, we were encouraged to investigate the roles and mechanisms of JOSD2 in NSCLC.

LKB1 (Liver kinase B1), a commonly recognized tumor suppressor in NSCLC, functions as a serine/threonine kinase that phosphorylates and activates the AMP-activated protein kinase (AMPK) to suppress cell proliferation. The kinase activity of LKB1 is controlled by a conformational change triggered by binding to STRAD and MO25.^9,10^ STRAD induces relocalization of LKB1 from nucleus to cytoplasm,^11,12^ while MO25 stabilizes the interaction of STRAD and LKB1.^12^ LKB1 is inactivated by point mutations or deletions in ~15–35% NSCLC patients.^13–15^ Notably, even for those cells harboring wild-type LKB1, the LKB1/AMPK kinase pathway may remain inactivated,^16,17^ since additional negative regulatory mechanism including epigenetic silencing, posttranslational modifications could also modulate the complex integrity and kinase activity. A recent study revealed that in hepatocellular carcinoma, K63-linked polyubiquitination on LKB1 by Skp2-SKP1/Cullin/F-box (SCF) ubiquitin ligase is critical for maintaining LKB1-STRAD-MO25 complex integrity and LKB1 activation.^18^ However, it remains elusive whether similar ubiquitination event can dictate the complex integrity and kinase activity of LKB1 in NSCLC models. Additionally, the corresponding DUB responsible for removing such ubiquitination on LKB1 and suppressing LKB1 kinase activity can be of great interest as an oncogenic factor and potential therapeutic target for NSCLC.

In this study, we performed a global profiling on the contribution of 97 DUBs in NSCLC patient survival possibilities by The Cancer Genome Atlas (TCGA) database and found that JOSD2 functioned as a deubiquitinase of LKB1. Further results showed that JOSD2-mediated removal of K6-linked polyubiquitination on LKB1 lysine residues 191, 269 and 423 resulted in disruption of LKB1-STRAD-MO25 complex integrity and inhibition of LKB1 kinase activity in NSCLC. Moreover, suppression of JOSD2 by RNA interference or pharmacological inhibitors significantly impeded the growth of NSCLC both in vitro and in vivo. Taken together, our study not only identified JOSD2 as an important tumor-promoting factor that inhibits LKB1 kinase activity through deubiquitination of LKB1 in NSCLC, but also provided a potential therapeutic strategy in treating NSCLC patients by targeting JOSD2 with inhibitors.

## Results

### JOSD2 is overexpressed and associated with poor prognosis in NSCLC patients

**Fig. 1:**
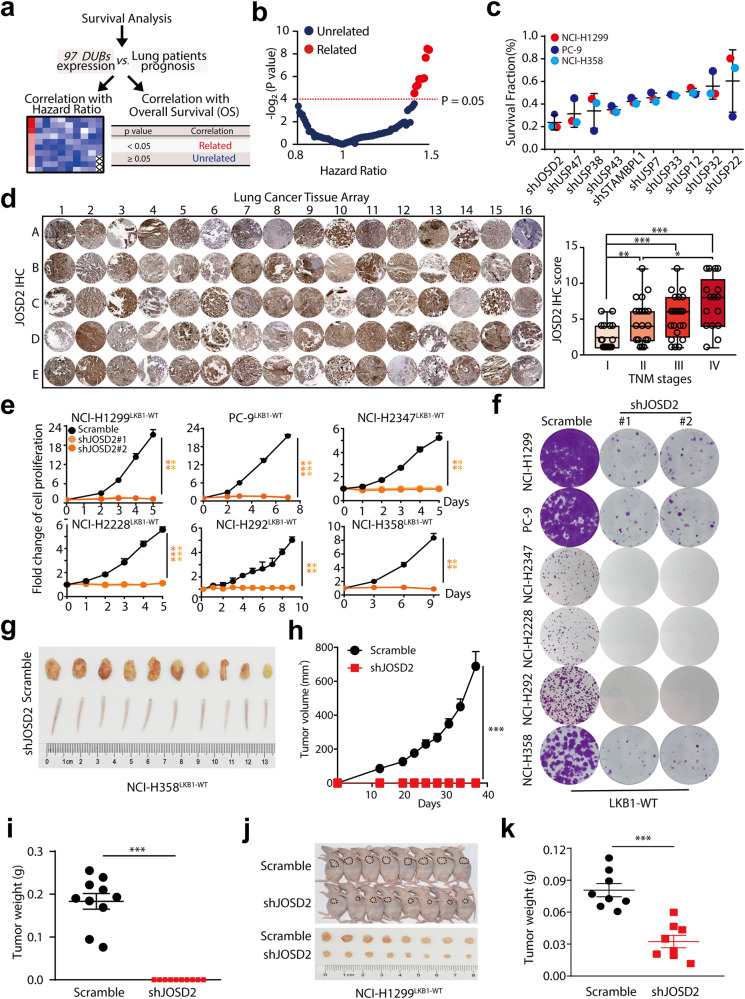
JOSD2 is overexpressed and associated with poor prognosis in NSCLC patients. **a** Schematic illustration of global survival analysis. TCGA analysis of overall survival (OS) in NSCLC patients separated by the expression of 97 *DUBs* individually. **b** Plot illustrating hazard ratio compared with *P* value (−log_2_) between the expression of 97 *DUBs* and OS in NSCLC patients. **c** Cell survival assays in NCI-H1299, PC-9 and NCI-H358 cells transfected with scramble control or indicated DUBs shRNA. The cells were infected with lentiviruses packaged scramble control or indicated DUBs shRNA for 96 h, then these cells were seeded 4000 cells per well in 96-well plates and cultured for 48 h, followed by SRB staining. **d** Immunohistochemistry (IHC) staining of JOSD2 in a lung cancer tissue array consisting of LUADs (*n* = 80) (left). Analysis of JOSD2 IHC staining in correlation to TNM stages (right). The evaluation of the IHC staining was performed by pathologist who is blind from the stage information of these patients, IHC score was evaluated by multiplication of positive staining proportions (1 score, <25%; 2 score, 25–50%; 3 score, 50–75%; 4 score, 75–100%) and intensity of protein expressions (1 score, weak staining; 2 score, moderate staining; 3 score, high staining). **e** Cell proliferation assay and **f** Colony formation assay in NCI-H1299, PC-9, NCI-H2347, NCI-H2228, NCI-H292 and NCI-H358 cells transfected with scramble control or JOSD2 shRNA (#1, #2). The indicated cell lines were infected with lentiviruses for 96 h, then these cells were seeded 1000 cells per well in 96-well plates or 6-well plates, followed by SRB staining (means ± SD, *n* = 3). **g** Images of scramble control and shJOSD2-expressing tumors of NCI-H358 xenografts (at the endpoint). **h** Tumor volume of scramble control and shJOSD2-expressing tumors of NCI-H358 xenografts, the tail of mice means the tumor is not growing. **i** Tumor weight of each mice in indicated groups (at the endpoint) of NCI-H358 xenografts (means ± SEM, *n* = 10). **j** Images of scramble control and shJOSD2-expressing tumors of NCI-H1299 xenografts (at the endpoint). **k** Tumor weight of each mice in indicated groups (at the endpoint) of NCI-H1299 xenografts (means ± SEM, *n* = 8). *P < 0.05; **P < 0.01; ***P < 0.001

To identify potential DUBs that can drive tumorigenesis in NSCLC, we globally analyzed the correlation of 97 *DUBs* mRNA levels with the hazard ratio and overall survival (OS) in NSCLC patients using TCGA database. Among these DUBs, 10 DUBs including USP7, USP22, USP38, JOSD2, USP33, USP12, USP47, USP43, USP33 and STAMBPL1 displayed a negative correlation with OS in NSCLC patients, suggesting potential tumor-promoting roles in NSCLC (Fig. 1a, b and Supplementary Fig. S1a, b). To identify the DUB most closely associated with NSCLC cell growth, we knocked down these DUBs one by one in 3 NSCLC cell lines including NCI-H1299, NCI-H358 and PC-9, and then compared their inhibitory effects on cell growth, and found that depletion of JOSD2 posed a strong inhibitory effect on these 3 cell lines (Fig. 1c). JOSD2 belongs to the MJD subfamily of DUBs along with other three cysteine-proteases, Ataxin-3, Ataxin-3L and JOSD1.^6^ Furthermore, data mining using TCGA database showed that the mRNA levels of *JOSD2* and *Ataxin-3* was significantly elevated in NSCLC compared to normal tissue, to a greater extent than that of the other MJD members, but only *JOSD2*, not *JOSD1* and *Ataxin-3*, was significantly associated with poor overall survival of NSCLC patients (Supplementary Fig. S1b–d). Additional analysis using the SurvExpress database (http://victortrevino.bioinformatics. mx:8080/Biomatec/SurvivaX.jsp) revealed that the expression levels of *JOSD2* were also remarkably correlated with poor prognosis of lung adenocarcinomas (LUADs, *P* < 0.001) and squamous cell carcinomas (LUSCs, *P* < 0.001) (Supplementary Fig. S1e).^19^

To further validate the overexpression of JOSD2 in NSCLC, we performed IHC staining to evaluate the JOSD2 protein levels in a lung cancer tissue array consisting of 80 LUADs (Fig. 1d and Supplementary Table S1). JOSD2 protein levels were significantly correlated with TNM stages of LUADs, as increasing JOSD2 expressions were associated with more advanced cancer stages (Fig. 1d and Supplementary Fig. S1f). The multivariate analysis of these 80 LUADs suggests that JOSD2 protein levels might not serve as an independent prognostic factor for NSCLC (Supplementary Fig. S1g). Further studies expanding the sample sizes are required to affirm the potential roles of JOSD2 as a predictive factor for NSCLC.

### JOSD2 depletion impairs NSCLC growth in vitro/vivo

Next, we performed a series of experiments to demonstrate JOSD2 influenced NSCLC growth in vitro/vivo. Firstly, we constructed two specific shRNAs independently targeting JOSD2. To verify the specificity of JOSD2 shRNAs, we investigated their effects on other family members and found that JOSD2 shRNAs only significantly downregulate JOSD2 levels but pose little effects on JOSD1, Ataxin-3 and Ataxin-3L (Supplementary Fig. S2a). Subsequently, we silenced endogenous JOSD2 in multiple NSCLC cell lines (NCI-H1299, PC-9, NCI-H2347, NCI-H2228, NCI-H292 and NCI-H358) and found that JOSD2 depletion significantly inhibited the cell proliferation and colony formation in these NSCLC cells, but impose minimal effect on human normal lung epithelial cells BEAS-2B (Fig. 1e, f and Supplementary Fig. S2b, c). To validate the influence of JOSD2 on tumor formation in vivo, nude mice were subcutaneously transplanted with NCI-H358 cells transfected with scramble control or JOSD2 shRNA. As shown in Fig. 1g–i and Supplementary Fig. S2d, JOSD2 depletion resulted in a robust inhibition of tumor formation in the NCI-H358 xenografted tumors. Similarly, on a pre-existing NCI-H1299 tumor models, intratumor injection of lentiviral vector delivering JOSD2 shRNA exhibited significant suppressive effect on the tumor growth, with inhibition ratio as 59.72% (*P* < 0.001) (Fig. 1j–k and Supplementary Fig. S2e).

Taken together, these data confirmed that depletion of JOSD2 significantly arrested the NSCLC cell proliferation in vitro and tumor growth in vivo.

### JOSD2 negatively regulates LKB1/AMPK signaling

**Fig. 2:**
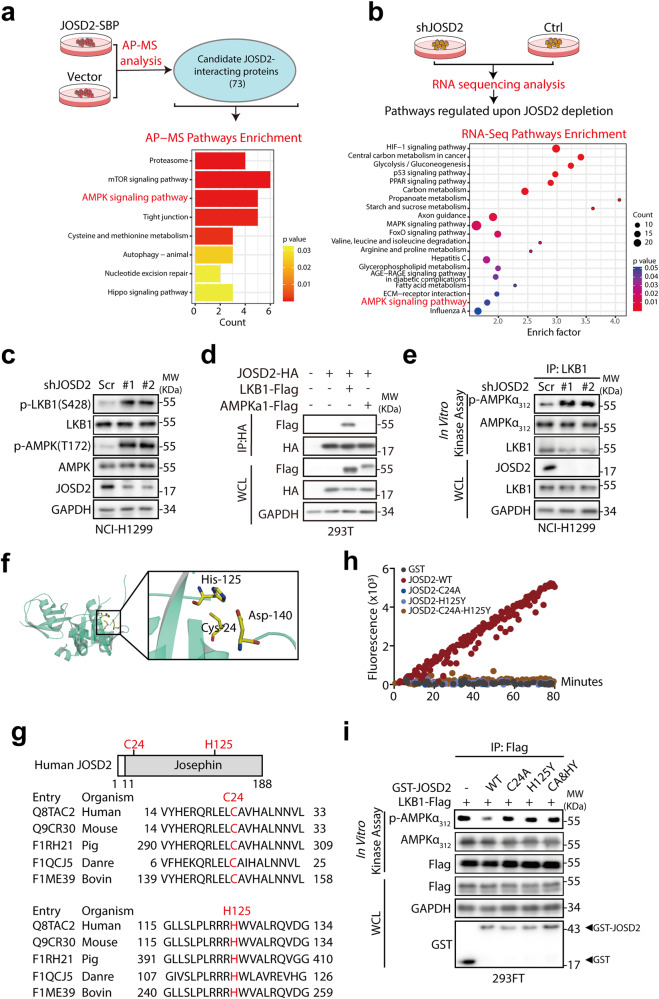
JOSD2 depletion activates the LKB1/AMPK pathway and LKB1 kinase activity. **a** IPs by Streptavidin magnetic beads from 293T cells transfected with Vector or JOSD2-SBP for 48 h were subjected to AP-MS analysis of JOSD2-interacting proteins. **b** NCI-H1299 cells were infected with lentiviruses of scramble control or JOSD2 shRNA for 96 h and the cell lysates were subjected to RNA sequencing analysis of related pathways regulated upon JOSD2 depletion. **c** NCI-H1299 cells were infected with lentiviruses of scramble control or JOSD2 shRNA (#1, #2) for 96 h and the cell lysates were subjected to IB. **d** Immunoprecipitates (IPs) by HA beads from 293FT cells transfected with the vector or JOSD2-HA along with LKB1-Flag or AMPKa1-Flag for 48 h were subjected to IB. **e** IPs by anti-LKB1 antibody from NCI-H1299 cells expressing scramble control or JOSD2 shRNA (#1, #2) were subjected to in vitro kinase assay followed by IB. **f** The position of cysteine 24, histidine 125 and aspartic acid 140 in human JOSD2 (PDB: 6PGV). **g** Schematic illustration of human JOSD2 protein marked with two candidate catalytic sites including cysteine 24 (C24) and histidine 125 (H125) (top). Sequence alignment of C24 and H125 catalytic sites within JOSD2 orthologues in different species (bottom). **h** In vitro ubiquitin-AMC assay by incubating purified GST, GST-JOSD2 or the indicated catalytic-inactive GST-JOSD2 protein with ubiquitin-AMC as the substrate at 37 °C for 1 h. **i** IPs by Flag beads from 293FT cells transfected with LKB1-Flag for 48 h were incubated with purified GST, GST-JOSD2 or the indicated catalytic-inactive GST-JOSD2 proteins at 37 °C for 1 h, and then subjected to in vitro kinase assay followed by IB

We next sought to investigate the mechanism by which JOSD2 promotes NSCLC growth. Firstly, we explored the JOSD2 expression levels of multiple NSCLC cell lines as well as normal lung epithelial cell BEAS-2B and found that JOSD2 level was extremely low in BEAS-2B compared to these NSCLC cell lines (Supplementary Fig. S3a). To identify the potential substrate(s) and the signaling pathways regulated by JOSD2 in an unbiased manner, we performed three sets of independent experiments: (1) AP-MS and LC-MS to detected the JOSD2-interacting proteins (Fig. 2a, Supplementary Fig. S3b and Supplementary Tables S2 and 3); (2) RNA sequencing analyses of NCI-H1299 cells (harboring high JOSD2 levels) with or without JOSD2 shRNA (shJOSD2 *vs*. Ctrl, duplicate) to explore the signaling pathways modulated by JOSD2 (Fig. 2b). By analysis, we found multiple tumor-related pathways were enriched, so we further examined the key effectors of these pathway upon depletion of JOSD2, and found that these cancer-associated pathways experienced varying degrees of upregulation or downregulation (Supplementary Fig. S3c). Among them, YAP and TAZ, the downstream effectors of the Hippo pathway, were mostly downregulated upon silencing JOSD2. This observation was consistent with our previous study, where we identified YAP/TAZ as the substrates for JOSD2 in cholangiocarcinoma.^20^ While phosphorylation of LKB1 at Ser428 and AMPKα at Thr172, two well-established readouts of LKB1/AMPK pathway, were predominantly upregulated upon JOSD2 knockdown (Fig. 2c and Supplementary Fig. S3c). More importantly, we found only AMPK pathway was enriched among the Top pathways in all the three analyses (Fig. 2a, b and Supplementary Fig. S3b–e), further substantiating its critical roles in NSCLC. Taken together, these results suggested that JOSD2 plays a key role in LKB1/AMPK signal transduction. Next, we asked how JOSD2 regulates this pathway. Our immunoprecipitation results showed that JOSD2 only interacts with LKB1, but not AMPK (Fig. 2d), suggesting that JOSD2 regulates LKB1/AMPK signaling pathway through interaction with LKB1.

As the kinase activity is critical for LKB1 to exert its tumor suppressor function,^21,22^ we then examined whether JOSD2 imposes negative regulatory effect on LKB1 by measuring LKB1 kinase activity toward AMPKα in vitro. Intriguingly, LKB1 protein purified from JOSD2-depleted NCI-H1299 cells displayed an increased ability to phosphorylate AMPKα (Fig. 2e and Supplementary Fig. S3f), affirming that JOSD2 inhibited LKB1/AMPK signaling through the interaction with LKB1 and suppression of its kinase activity.

To further verify the causal link between JOSD2 and LKB1 activity, we evaluated the phosphorylation levels of LKB1 and AMPK along with JOSD2 protein levels across 13 different cell lines harboring wild-type LKB1. As shown in supplementary Fig. S3g, both the p-LKB1 and p-AMPK levels were inversely correlated with the protein levels of JOSD2 (r = −0.7512 for p-LKB1 *vs*. JOSD2 (*P* < 0.01), *r* = −0.7851 for p-AMPK *vs*. JOSD2 (*P* < 0.01)), supporting our notion that LKB1 kinase activity was negatively regulated by JOSD2.

Given that JOSD2 is classified as a DUB, we next investigated whether the catalytic activity of JOSD2 is required for LKB1 kinase activity inhibition. Cysteine 24 has previously been reported to be involved in JOSD2 catalytic activity as C24A (cysteine 24 mutated to alanine) mutant partially abolishes DUB activity of JOSD2.^23^ The crystal structure of JOSD2 has then been further revealed, indicating that it contains an important triad, which is critical for its DUB activity. This triad is composed of cysteine 24, histidine 125 and aspartic acid 140 (Fig. 2f),^24^ which distinguishes JOSD2 from JOSD1, Ataxin-3 and Ataxin-3L. As reported, the histidine residue can serve as an acid and attract proton from nucleophilic center cysteine and thus contribute to the DUB activity.^25^ We therefore speculated that histidine 125 might also contribute to the DUB activity of JOSD2 since it is also a proton acceptor and thus introduced H125Y (histidine 125 mutated to tyrosine) mutant in our study. Interestingly, sequence comparison revealed that both cysteine 24 and histidine 125 are conserved in JOSD2 orthologs across different species (Fig. 2g). Subsequent in vitro ubiquitin-AMC assay showed that the catalytic activity of JOSD2 was impaired in JOSD2 C24A, H125Y and the double mutants, suggesting that both cysteine 24 and histidine 125 were important catalytic-active sites of JOSD2 (Fig. 2h).

To determine whether the DUB activity of JOSD2 is required for its inhibition on LKB1 kinase activity, we purified recombinant human GST-JOSD2 protein and its catalytic-inactive mutants. Only JOSD2-WT, but not its catalytic-inactive mutants resulted in decreased phosphorylation of AMPKα in vitro (Fig. 2i and Supplementary Fig. S3h).

Collectively, these results indicated that JOSD2 negatively regulates LKB1 kinase activity in NSCLC cells through its deubiquitination activity and residues cysteine 24 and histidine 125 of JOSD2 are both important catalytic sites.

### JOSD2 binds and removes K6-linked polyubiquitination of LKB1

**Fig. 3:**
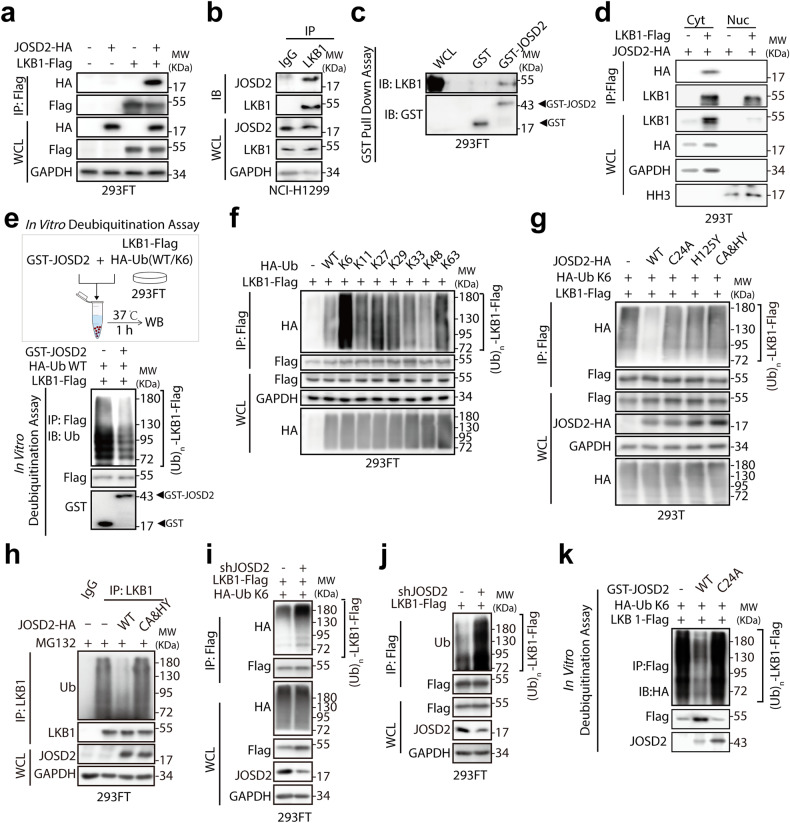
JOSD2 deubiquitinates LKB1 via K6 linkage. **a** IPs by Flag beads from 293FT cells transfected with vector or JOSD2-HA along with vector or LKB1-Flag for 48 h were subjected to IB. **b** IPs by control IgG or anti-LKB1 antibody from NCI-H1299 cells were subjected to IB. **c** Cell lysates of 293FT cells were incubated with purified GST or GST-JOSD2 protein coupled to GSH-Sepharose at 4 °C for 2 h. Proteins retained on Sepharose were subjected to IB. **d** 293T cells transfected with LKB1-Flag along with JOSD2-HA for 48 h were subjected to nucleoplasmic separation and the cell lysates were subjected to IPs. **e** Schematic illustration of in vitro deubiquitination assay (top). Ubiquitinated LKB1 with HA-tagged wild-type ubiquitin (WT-Ub) incubated with purified GST or GST-JOSD2 protein at 37 °C for 1 h were subjected to in vitro deubiquitination assay, followed by IB (bottom). **f** IPs by Flag beads from 293FT cells transfected with LKB1-Flag along with WT-Ub or indicated ubiquitin mutants for 48 h were subjected to IB. **g** IPs by Flag beads from 293T cells transfected with LKB1-Flag and HA-tagged ubiquitin-K6 (HA-Ub K6) along with JOSD2-WT or indicated JOSD2 catalytic-inactive mutants for 48 h were subjected to IB. **h** IPs by LKB1 antibody or normal IgG from 293FT cells transfected with JOSD2-WT/CA&HY for 48 h were subjected to IB. **i** 293FT cells expressing scramble control or JOSD2 shRNA were transfected with LKB1-Flag along with HA-ub K6 for 48 h and the cell lysates were subjected to IPs. **j** 293FT cells expressing scramble control or JOSD2 shRNA were transfected with LKB1-Flag for 48 h and the cell lysates were subjected to IPs. **k** Ubiquitinated LKB1 with HA-Ub K6 incubated with purified GST or GST-JOSD2/C24A protein at 37 °C for 1 h were subjected to in vitro deubiquitination assay, followed by IB

To gain further insight on how JOSD2 regulates LKB1 kinase activity through its DUB activity, we first examined the interaction between exogenously expressed JOSD2 and LKB1 (Fig. 3a). Our result clearly indicated that overexpressed JOSD2 binds to LKB1. We then checked the endogenous interaction between JOSD2 and LKB1 and found that endogenous JOSD2 co-immunoprecipitated with LKB1 (Fig. 3b). Moreover, in GST pull down assay, we found that LKB1 protein purified from 293FT cell lysates overexpressing LKB1 could be pulled down by recombinant human GST-JOSD2 protein, but not GST protein (Fig. 3c), further confirming that JOSD2 interacts with LKB1. In addition, the interaction between JOSD2 and LKB1 was found to be more abundant in the cytoplasm than that in the nuclear fraction (Fig. 3d).

Using an in vitro deubiquitination assay, we then demonstrated that GST-JOSD2 efficiently removes the wild-type ubiquitin (WT-Ub) linkage from LKB1 (Fig. 3e and Supplementary Fig. S4a). Interestingly, total protein levels of LKB1 remained unchanged when JOSD2 protein levels were manipulated (Fig. 2c), indicating that the polyubiquitination on LKB1 removed by JOSD2 was non-degradative linkage. To pinpoint the specific lysine linkage on LKB1 mediated by JOSD2, we performed a cell-based ubiquitination assay using WT-Ub and seven mutant forms of ubiquitin: ubiquitin-K6, ubiquitin-K11, ubiquitin-K27, ubiquitin-K29, ubiquitin-K33, ubiquitin-K48 and ubiquitin-K63, each links to the substrate in a distinctive manner. As shown in Fig. 3f and Supplementary Fig. S4b, LKB1 was ubiquitinated through K6, K27, K29 and K63 linkages, and among these, K6 was the most abundant ubiquitin linkage type. In addition, only the JOSD2-WT, but not the JOSD2 catalytic-inactive mutants, decreased K6-linked or the endogenous polyubiquitination of LKB1 (Fig. 3g, h, Supplementary Fig. S4c). Consistently, JOSD2 knockdown significantly increased the levels of K6-linked or endogenous polyubiquitination of LKB1, respectively (Fig. 3I, j). To further validate the DUB activity of JOSD2 toward LKB1, we performed an in vitro deubiquitination assay and the results showed that GST-JOSD2 recombinant protein, but not GST-JOSD2-C24A, successfully removed K6-linked polyubiquitination of LKB1 (Fig. 3k, Supplementary Fig. S4d). Subsequently, we tried to explore the potential E3 ligase mediated K6-ub for LKB1 and we introduced exogenous SKP2, the only reported E3 ubiquitin ligase for LKB1, and found that SKP2 imposed almost no effect on LKB1 K6 linkage (Supplementary Fig. S4e). Given that the previous report identified SKP2 as the E3 ubiquitin ligase for LKB1 K63-linked ubiquitination, it is possible that these two ubiquitin linkages utilized differential E3 ubiquitin ligases, and further study is required to explore and identify the E3 ligase responsible for LKB1 K6 linkage in the future. In addition, to better consolidate our conclusion that JOSD2 regulated LKB1 activity by removing K6-ub but not K63-ub, we also investigated the effects of JOSD2 on K63-ub of LKB1, as shown in supplementary Fig. S4f, g, neither overexpression nor depletion of JOSD2 posed effects on LKB1 K63-ub, suggested that as a DUB for LKB1, JOSD2 preferentially cleaved K6 linkage but not K63 linkage.

Hence, these results indicated that JOSD2 was a bona fide DUB for LKB1, as JOSD2 bound to LKB1 and removed the K6-linked polyubiquitination on LKB1.

### K6-linked polyubiquitination is required for LKB1 activation

**Fig. 4:**
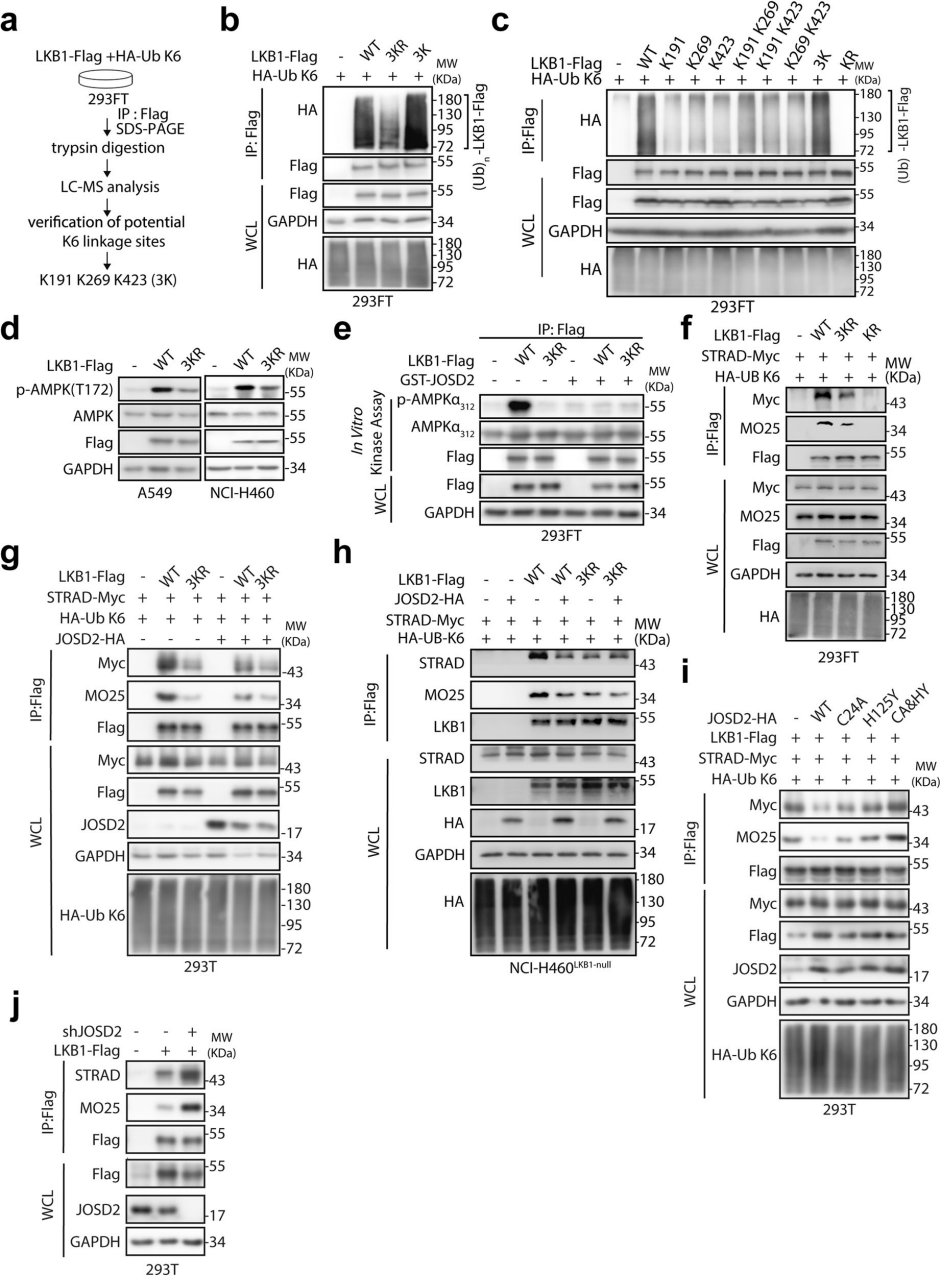
JOSD2 disrupts the LKB1 complex integrity by removing K6-linked polyubiquitination of LKB1. **a** Schematic representation of mass spectrometry analysis to identify LKB1 K6-linked polyubiquitination sites. **b**, **c** IPs by Flag beads from 293FT cells transfected with indicated LKB1-Flag and HA-Ub K6 for 48 h were subjected to IB. **d** LKB1-deficient A549 and NCI-H460 cells stably transfected with the indicated LKB1-Flag were subjected to IB. **e** IPs by Flag beads from 293FT cells transfected with the indicated LKB1-Flag for 48 h were incubated with purified GST or GST-JOSD2 protein at 37 °C for 1 h, and then subjected to in vitro kinase assay followed by IB. **f** IPs by Flag beads from 293FT cells transfected with STRAD-Myc, HA-Ub K6 and the indicated LKB1-Flag for 48 h were subjected to IB. **g** IPs by Flag beads from 293T cells transfected with vector or JOSD2-HA along with STRAD-Myc, HA-Ub K6 and indicated LKB1-Flag for 48 h were subjected to IB. **h** IPs by Flag beads from NCI-H460 cells transfected with vector or JOSD2-HA along with STRAD-Myc, HA-Ub K6 and indicated LKB1-Flag for 48 h were subjected to IB. **i** IPs by Flag beads from 293T cells transfected with vector, JOSD2-WT or indicated JOSD2 catalytic-inactive mutants along with the STRAD-Myc, HA-Ub K6 and LKB1-Flag for 48 h were subjected to IB. **j** 293T cells expressing scramble control or JOSD2 shRNA were transfected with LKB1-Flag for 48 h and the cell lysates were subjected to IPs. K191/K269/K423: mutated all the lysines but K191/K269/K423 at LKB1 with arginines; K191 K269/ K269 K423/ K191 K423: mutated all the lysines but K191 and K269 or K269 and K423 or K191 and K423 at LKB1 with arginines; 3 K: mutated all the lysines but K191, K269 and K423 at LKB1 with arginines; 3KR: mutated the lysines of K191, K269 and K423 at LKB1 with arginines; KR: mutated all lysines with arginines

We next attempted to identify the sites of K6 linkage ubiquitin chains on LKB1. We performed immunoprecipitation of exogenously expressed LKB1 from 293FT cell line co-transfected with ubiquitin-K6, which was then subjected to proteolytic digestion and liquid chromatography-mass spectrometry (LC-MS) analysis (Fig. 4a). The LC-MS analysis identified lysine residues 191 (K191), 269 (K269) and 423 (K423) as three promising K6-linked polyubiquitination sites on LKB1 (Supplementary Fig. S5; Supplementary Table S4). To verify these K6 linkage sites, we constructed a LKB1 mutant carrying three K-to-R substitutions at the positions 191, 269 and 423 (LKB1-3KR). As shown in Fig. 4b, LKB1-3KR displayed much less K6-linked polyubiquitination compared to wild-type LKB1. To further confirm this, we mutated all the lysines at LKB1 with arginines (LKB1-KR). While the single R-to-K mutants and double R-to-K mutants partially restored the K6-linked polyubiquitination at LKB1, only the triple R-to-K mutant of LKB1 (LKB1-3K), displayed similar levels of K6-linked polyubiquitination compared to LKB1-WT (Fig. 4c). On the contrary, K-to-R mutants on other lysine residues on LKB1 showed little effect on K6-linked polyubiquitination compared to K191, 269 and 423 (Supplementary Fig. S6a). We also tested lysine residues 41, 44, 48, 62 and 64, which were reported previously for mediating K63-linked polyubiquitination of LKB1.^18^ We found that the 5KR mutant of LKB1 (LKB1-5KR, K63 linkage-deficient mutant) displayed no significant alteration on K6-linked polyubiquitination (Fig. S6a). Interestingly, sequence comparison also revealed that K191, K269, K423 are conserved in LKB1 orthologs across different species (Supplementary Fig. S6b).

We then asked whether JOSD2-mediated deubiquitination of LKB1 via K6 linkage is critical for inhibiting LKB1 kinase activity. Stably expressed LKB1-WT displayed robust activation of the AMPK phosphorylation, while the LKB1-3KR mutant significantly impeded the LKB1 kinase activity to induce AMPK signaling (Fig. 4d, Supplementary Fig. S6c). To further demonstrate that JOSD2 regulates the kinase activity of LKB1 through its DUB activity, we performed an in vitro kinase activity assay by incubating LKB1-3KR and LKB1-WT with recombinant GST-JOSD2 protein. Notably, GST-JOSD2 significantly diminished the kinase activity of LKB1 but showed only slight effect on LKB1-3KR, suggesting that the three lysine residues responsible for K6-linked ubiquitination on LKB1 are critical in mediating JOSD2 modulation on LKB1 kinase activity (Fig. 4e).

Altogether, these results suggested that JOSD2 suppresses LKB1 kinase activity by removing the K6-linked polyubiquitination on lysine residues 191, 269 and 423 of LKB1.

### JOSD2 disrupts the LKB1 complex integrity by removing K6-linked polyubiquitination of LKB1

To further delineate the molecular mechanism by which JOSD2 regulates LKB1 kinase activity upon removal of K6-linked polyubiquitination, we investigated the integrity of LKB1-STRAD-MO25 complex as LKB1 is mainly activated through the formation of the heterotrimeric complex. We found that the interacting ability of LKB1-3KR with STRAD and MO25 was more greatly impaired when compared to that of LKB1-5KR, implicating the important roles of K6-ub for LKB1 activity. Moreover, LKB1-KR displayed most greatly impaired activity when compared to that of LKB1-3KR and LKB1-5KR, which mainly due to LKB1-KR lacking K6-ub and K63-ub modification both of which mediated the formation of LKB1-STRAD-MO25 complex (Fig. 4f, Supplementary Fig. S6d, e). Furthermore, JOSD2 overexpression attenuated the interaction of LKB1-WT, but not LKB1-3KR, with STRAD and MO25 (Fig. 4g, h, Supplementary Fig. S6f). More importantly, only overexpression of JOSD2-WT, but not JOSD2 catalytic-inactive mutants, could interrupt the LKB1 interaction with STRAD and MO25 (Fig. 4i, Supplementary Fig. S6g), suggesting that the deubiquitination activity is required for modulating LKB1 complex integrity. In line with this, depletion of JOSD2 enhanced the complex integrity by strengthening the interaction of STRAD and MO25 with LKB1 (Fig. 4j and Fig. S6h).

We further introduced immunofluorescent assay to investigate the influence of LKB1-3KR mutant on subcellular localization. As shown in Supplementary Fig. S6i, LKB1-WT was mainly localized in the cytoplasm; however, LKB1-3KR was predominantly localized in the nucleus. Given that LKB1-3KR failed to form the ternary complex with STRAD and MO25 and impeded the kinase activity of LKB1, the nuclear retention of LKB1-3KR further confirmed these observations since nuclear localized LKB1 is generally inactivated.

Taken together, K6-linked polyubiquitination of LKB1 dictates its kinase activity by maintaining the integrity of the LKB1-STRAD-MO25 complex, while JOSD2 impedes LKB1 kinase activity by removing K6-linked polyubiquitination which in turn disrupts the heterotrimeric complex integrity.

### JOSD2 promotes NSCLC growth via regulating LKB1

**Fig. 5:**
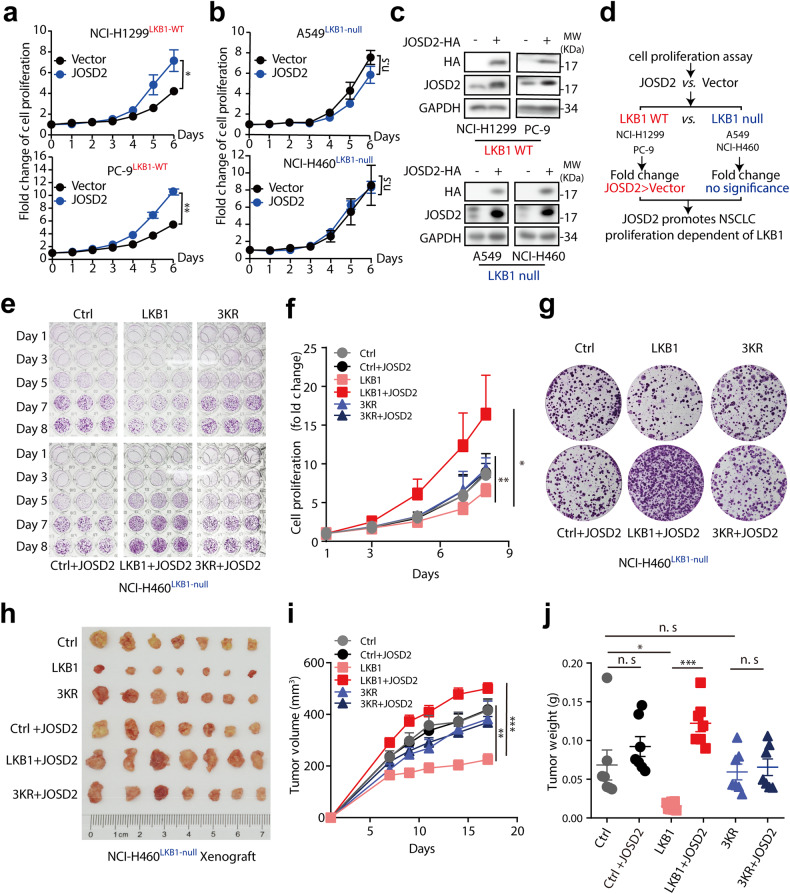
JOSD2 promotes NSCLC growth via regulating LKB1. **a** Cell proliferation assay of LKB1-WT cells (NCI-H1299 and PC-9) and **b** LKB1-null cells (A549 and NCI-H460) transfected with vector control or JOSD2. The indicated cell lines were infected with lentiviruses for 96 h, then these cells were seeded 1000 cells per well in 96-well plates or 6-well plates, followed by SRB staining. (means ± SD, *n* = 3). **c** Immunoblotting analysis showing the overexpression efficiency of JOSD2. **d** Schematic illustration of studies and results in (**a**)–(**c**). **e**–**g** Cell proliferation and colony formation of NCI-H460 cells harboring LKB1-null/WT/3KR transfected with JOSD2. NCI-H460 cells were infected with lentiviruses packaged Vector/LKB1-WT/3KR for 24 h and supplemented with fresh culture medium. After 3 days, these cells were selected by treating with the culture medium containing 5 μg/mL puromycin for 24 h. Then these cells were infected with lentiviruses packaged Vector/JOSD2 for 24 h. After 72 h, these cells were seeded 1000 cells per well in 96-well plates or 6-well plates, followed by SRB staining. (means ± SD, *n* = 5). **h**–**j** The in vivo effect of JOSD2 overexpression on NCI-H460 xenograft tumor growth studies. **h** Tumors images of indicated groups (at the endpoint). (*n* = 7). **i** Tumor volume of indicated groups. (means ± SEM, *n* = 7). **j** Tumor weight of each mice in indicated groups (at the endpoint). (mean ± SEM, *n* = 7). n.s: *P* > 0.05; *P < 0.05; **P < 0.01; ***P < 0.001

We then further ask whether the tumor-promoting effect of JOSD2 is dependent on its regulatory effect on LKB1. As expected, JOSD2 overexpression significantly promoted cell proliferation of LKB-WT NSCLC cells NCI-H1299 and PC-9, with no appreciable change in LKB-null cells A549 and NCI-H460 (Fig. 5a–d). Moreover, the effects of JOSD2 promoting LKB-WT NSCLC cells/xenografts growth in vitro/vivo depends on its enzymatic activity (Supplementary Fig. S7a–c). Correspondingly, JOSD2 depletion significantly inhibited the cell proliferation in these NSCLC cells harboring LKB-WT (Fig. 1e), whereas imposed a weaker effect on LKB-null cells A549 cells at least under our experimental condition (Supplementary Fig. S7d, e). While the exogenous introduction of LKB1 evidently increased the susceptibility of A549 cells towards JOSD2 knockdown (Supplementary Fig. S7d, e). Nonetheless, in LKB1-null cells, silencing JOSD2 still exhibited a certain inhibitory effect on the proliferation, suggesting that other protein substrates in addition to LKB1, might also be involved in JOSD2-driven NSCLC proliferation and cannot be excluded.

To further demonstrate that the tumor-promoting effect of JOSD2 depends on LKB1, LKB1-WT and LKB1-3KR were introduced to LKB-null NCI-H460 cells. We found JOSD2 distinctly promoted the cell proliferation and colony formation in NCI-H460 harboring LKB1-WT, but with no appreciable change in vector or LKB1-3KR groups (Fig. 5e–g, Supplementary Fig. S7f, g). More importantly, JOSD2 distinctly accelerated the in vivo tumor growth in NCI-H460 xenografted tumors harboring LKB1-WT, but exerting minimal impact in control and LKB1-3KR groups (Fig. 5h–j). Taken together, the tumor-promoting effect of JOSD2 is dependent, at least partially, on its regulation on LKB1.

### JOSD2 depletion inhibits NSCLC patient-derived xenograft growth by activating LKB1

**Fig. 6:**
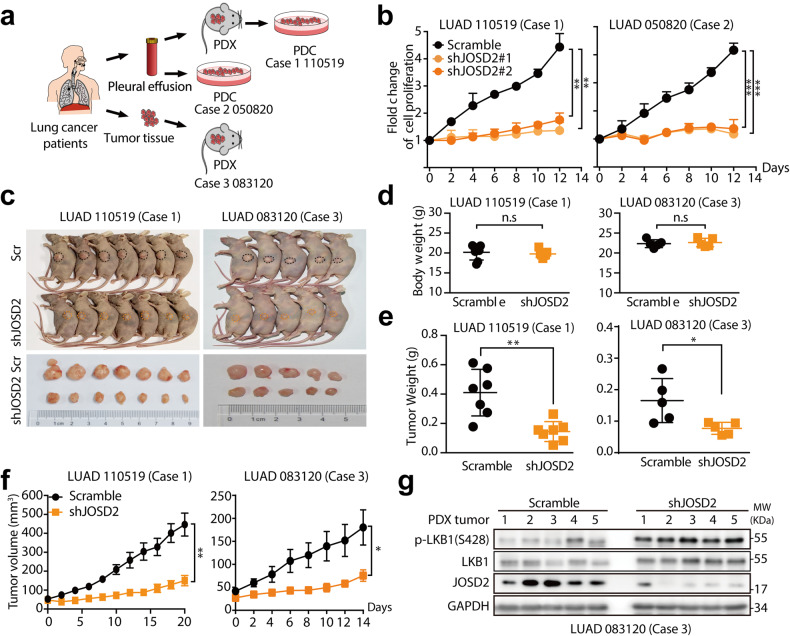
JOSD2 depletion suppresses the growth of patient-derived cells and patient-derived xenografts. **a** Schematic illustration of patient-derived cell (PDC) and patient-derived xenograft (PDX) models. The schematic diagram was generated from online tools of BioRender (https://app.biorender.com). **b** Cell proliferation assay in two PDC cells expressing scramble control or JOSD2 shRNA (#1, #2). The PDCs were infected with lentiviruses for 96 h, then these cells were seeded 1000 cells per well in 96-well plates, followed by SRB staining. (means ± SD, *n* = 3) (means ± SD, *n* = 3). **c**, **g** The in vivo effect of shJOSD2 on PDX model. **c** Images of mice bearing scramble control and shJOSD2-expressing tumors (top). Images of scramble control and shJOSD2-expressing tumors (bottom) (at the endpoint). **d** Body weight of each mice in indicated groups (at the endpoint). (mean ± SEM). **e** Tumor weight of each mice in indicated groups (at the endpoint). (mean ± SEM). **f** Tumor volume of indicated groups was measured every 2 days (mean ± SEM). **g** Proteins extracted from PDX tumors of LUAD 083120 were subjected to IB. n.s, *P* > 0.05; *P < 0.05; **P < 0.01; ***P < 0.001

To further investigate the contribution of JOSD2 in NSCLC growth in preclinical settings, we used patient-derived cell (PDC) and patient-derived xenograft (PDX) models obtained from 3 cases of NSCLC patients harboring LKB1-WT (Fig. 6a). We found that JOSD2 depletion by RNA interference robustly inhibited the proliferation of both PDC cells in vitro (Fig. 6b), accompanied with the activation of p-LKB (Supplementary Fig. S8a). Consistently, intratumor injection of lentiviral vector delivering JOSD2 shRNA significantly delayed the tumor growth of both PDXs in vivo (Fig. 6c–f and Supplementary Fig. S8b and Supplementary Table S5).

To further clarify whether JOSD2 inhibition is capable of activating LKB1 kinase activity in the xenograft model, we examined intratumor-related protein levels and found a robust activation of LKB1 in shJOSD2-treated PDX tumors (Fig. 6g and Supplementary Fig. S8c). Furthermore, shJOSD2 robustly suppressed cell proliferation in the tumor regions of PDX as evidenced by the greatly reduced Ki-67 positive cells (Supplementary Fig. S8d). In addition, we further examined the effects of JOSD2 knockdown on cell cycle and found depletion of JOSD2 significantly induced cell cycle arrest (Supplementary Fig. S8e), moreover, the levels of p-ERK and p-Akt, two classical pathways associated with cell proliferation, were also significantly inhibited (Supplementary Fig. S3c). These findings are consistent with our in vitro finding that JOSD2 negatively regulates LKB1 pathway and promotes NSCLC cell growth dependent of LKB1.

These results collectively provided clinical relevance that JOSD2 plays a crucial role in promoting NSCLC cell proliferation through LKB1 kinase activity inhibition.

### Identification of HY041004 as potential inhibitor of JOSD2

In vitro ubiquitin-AMC assay is frequently used to measure DUB activity.^26–28^ This assay is based on the hydrolysis of ubiquitin-AMC by DUBs, releasing AMC, which can be monitored by fluorescence spectroscopy. To search for JOSD2 inhibitors, we employed a library of compounds consisting of 15 reported DUB inhibitors, 31 clinically-used drugs as well as 53 small molecules with novel chemical structures from our collaborator (Supplementary Table S6).

**Fig. 7:**
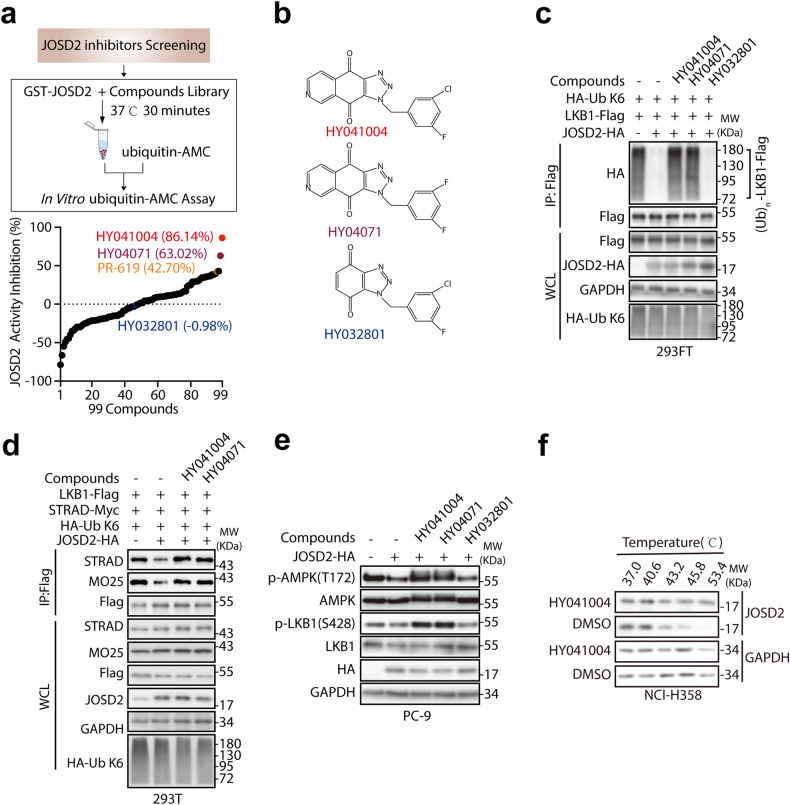
JOSD2 inhibitors abolish JOSD2 DUB activity. **a** Schematic illustration of JOSD2 inhibitors screening (top) and JOSD2 activity inhibition ratio of indicated compounds (*n* = 99) (bottom). Black dashed line indicates no inhibition on JOSD2 activity. 2 μM indicated compounds were incubated with GST-JOSD2 protein at 37 °C for 30 min and then subjected to in vitro ubiquitin-AMC assay. **b** Chemical structures of HY041004, HY04071 and HY032801. **c** 293FT cells transfected with LKB1-Flag, HA-Ub K6 and JOSD2-HA for 48 h were treated with indicated compounds for 4 h at concentration of 0.5 μM. IPs by Flag beads from cell lysates were subjected to IB. **d** 293T cells transfected with LKB1-Flag, STRAD-Myc, HA-Ub K6 and JOSD2-HA for 48 h were treated with indicated compounds for 4 h at concentration of 0.5 μM. IPs by Flag beads from cell lysates were subjected to IB. **e** PC-9 cells stably transfected with JOSD2-HA were treated with indicated compounds for 4 h at concentration of 0.5 μM and cell lysates were subjected to IB. **f** NCI-H358 cells were incubated with DMSO or 40 μM HY041004 and subjected to cellular thermal shift assay. Cell lysates were lysed by RIPA lysis buffer and then subjected to IB

We tested their inhibitory effect at the concentration of 2 μM on JOSD2 DUB activity using an in vitro ubiquitin-AMC assay with recombinant GST-JOSD2 (Fig. 7a; Supplementary Table S6). PR-619, a pan inhibitor of DUB,^29^ was introduced as a positive control (JOSD2 activity inhibition ratio = 42.70%). Among the 99 compounds, HY041004^30^ and HY04071^30^ (Fig. 7b) stood out as promising JOSD2 inhibitors with the most potent inhibitory effect against JOSD2 catalytic activity as 86.14% and 63.02% respectively. On the contrary, compound HY032801 that showed little effect (−0.98%) was used as “negative control” compound in the following studies. Further cellular assays revealed that both HY041004 and HY04071 robustly blocked the loss of K6-linked polyubiquitination on LKB1 caused by JOSD2 (Fig. 7c), suggesting that these two compounds not only inhibit the in vitro deubiquitinating activity of JOSD2, but also efficiently suppress its catalytic activity in the cells. In addition, HY041004 and HY04071 were able to restore the LKB1 complex integrity (Fig. 7d) and kinase activity (Fig. 7e) in JOSD2 overexpressed cells. Among the two inhibitors, HY041004 was more potent in suppressing JOSD2 catalytic activity (IC_50_ values are 0.26 μM and 0.93 μM for HY041004 and HY04071, respectively; Supplementary Fig. S9a). To investigate the on-target engagement of HY041004 in cancer cells, we also conducted cellular thermal shift assay.^31^ Compared with the DMSO control, HY041004 stabilized JOSD2 at denaturation temperatures ranging from 37.0 °C to 53.4 °C, which demonstrated the interaction of HY041004 and JOSD2 (Fig. 7f).

To clarify the specificity of HY041004 on JOSD2, we compared its influence on the 4 MJDs family members, and found that this compound exhibited the most potent inhibitory effects against JOSD2, a less but comparable inhibition on JOSD1 with IC_50_ value as 0.47 μM, and a much more reduction of suppression on the other two MJDs Ataxin-3 and Ataxin-3L, with IC_50_ values as 5.4 and 12.8 μM, respectively (Supplementary Fig. S9b).

### HY041004 inhibits NSCLC growth in vitro/vivo

**Fig. 8:**
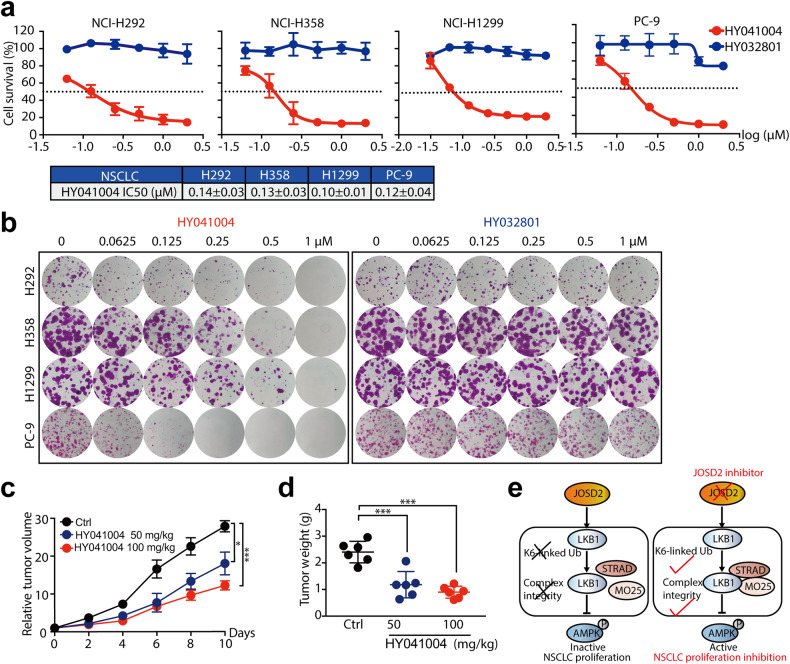
HY041004 efficiently suppresses NSCLC growth in vitro/vivo. **a** Proliferation assay and **b** Colony formation assay in NCI-H292, NCI-H358, NCI-H1299 and PC-9 cells. The indicated cell lines were treated with indicated compounds at a series of concentrations for 72 h, followed by cell proliferation assay and colony formation assay. Black dashed line indicates 50% of cell survival. (means ± SD, *n* = 3). **c**, **d** The in vivo effect of HY041004 on NCI-H1299 xenografted tumors (orally, twice daily, 50 mg/kg and 100 mg/kg) for indicated days. **c** Relative tumor volume of indicated groups was measured every 2 days (mean ± SEM. *n* = 6/group). **d** Tumor weight of each mice in indicated groups (at the endpoint). **e** Schematic illustration of pharmacological inhibitory mechanism of JOSD2 inhibitors. *P < 0.05; ***P < 0.001

To assess the in vitro pharmacological inhibitory effect of JOSD2 inhibitor, we first treated LKB1-WT NSCLC cell lines with indicated concentrations of HY041004 for 72 h. Our results showed that HY041004 prominently reduced cell viability and induced cell apoptosis in a concentration-dependent manner (Fig. 8a, b and Supplementary Fig. S9c, d). In contrast, negative control compound, HY032801, which possesses no JOSD2 inhibitory effect, failed to inhibit cell proliferation (Fig. 8a, b, Supplementary Fig. S9c).

Above-mentioned data showed that HY041004 may also suppressed JOSD1 DUB activities, with a less extent than that of JOSD2. Given the facts that: (1) JOSD1 expression was unrelated with the prognosis of NSCLC patients (Supplementary Fig. S1d); (2) JOSD1 depletion only induced a marginal reduction of cell proliferation (Supplementary Fig. S9e, f), we were encouraged to speculate that the anticancer activities of HY041004 may largely owing to its JOSD2-targeting effect.

To further demonstrate the inhibitory effect of HY041004 was dependent on JOSD2-LKB1 axis, we treated LKB1-null NSCLC cell lines with HY041004, and found the susceptibility of these five LKB1-null cell lines towards HY041004 were less than that in LKB1 wild-type NSCLC cell lines (Fig. 8a, Supplementary Fig. S10a). Intriguingly, the exogenous introduction of LKB1-WT, but not 3KR, could strengthen the anti-proliferative effect of HY041004 to a certain extent (Supplementary Fig. S10b), indicating the anticancer activity of HY041004 was largely dependent on the JOSD2-LKB1 axis.

Having shown HY041004 displayed potent anticancer effects in NSCLC cell in vitro, we next validated the in vivo efficacy. Treatment of tumor-bearing mice with orally administration of HY041004 significantly suppressed tumor growth and induced intratumor cell apoptosis, with T/C value as 43.88% (*P* < 0.001, *vs*. control) and inhibition ratio as 62.50% (*P* < 0.001, *vs*. control), for 100 mg/kg group (Fig. 8c, d, Supplementary Fig. S10c, Supplementary Table S7).

Collectively, we identified HY041004 as a novel small-molecule inhibitor for JOSD2 and confirmed its pharmacological inhibitory effect both in vitro/vivo, providing a viable therapeutic strategy in NSCLC patients by targeting JOSD2 with inhibitors (Fig. 8e).

## Discussion

As our understandings on DUBs and their roles in tumorigenesis deepened, targeting DUB has been regarded as a promising approach for cancer treatment. In hope of identifying critical DUB(s) as new therapeutic target for NSCLC, we performed a global profiling of 97 DUBs and discovered that JOSD2 played an important role in promoting cell proliferation of NSCLC both in vitro and in vivo. Mechanistically, we demonstrated that JOSD2 inhibits LKB1 kinase activity by removing K6-linked polyubiquitination of LKB1. The therapeutic potential of this newly identified JOSD2-LKB1 regulatory axis was then verified by both RNA interference and small-molecule inhibitors that abolish DUB activity of JOSD2 and exert potent anticancer activities.

JOSD2 belongs to the MJD subfamily of DUB that also includes Ataxin-3, Atanxin-3L and JOSD1. Ataxin-3 is overexpressed and promotes cell proliferation in both NSCLC^32^ and testicular cancer.^33^ Mechanically, Ataxin-3 inhibits the phosphatase and tensions homolog (PTEN) expression and then activates the AKT/mTOR pathway. Ataxin-3L has been demonstrated to be required for hepatocyte growth factor-induced growth of NSCLC.^34^ Recently, JOSD1 has been identified as the most significantly upregulated DUB during the development of chemo-resistance in gynecological cancer.^35^ JOSD1 deubiquitinates K48 linkage of myeloid cell leukemia 1 (MCL1) to stabilize its protein levels and then exerts anti-apoptosis effect. Recently, the roles of JOSD2 in malignant tumors have been gradually revealed. Our previous study revealed the pro-tumor function of JOSD2 in cholangiocarcinoma and found that JOSD2 deubiquitinates and stabilizes YAP/TAZ by cleaving the proteolytic ubiquitination and inhibiting the latter degradation.^36^ Huang et al. found that JOSD2 interacts with β-catenin and decreases the latter’s polyubiquitination levels, therefore promoting Wnt pathway activation.^37^ A recent study suggested that in a PHGDH defined metabolic subtype in lung adenocarcinomas that associates with poor prognosis, JOSD2 might function as a DUB to stabilize PHGDH protein levels.^7^ Krassikova et al. also demonstrated that JOSD2 promoted glycolysis in tumor cells through deubiquitination and stabilization of three important enzymes involved in glucose metabolism, namely PHGDH, PFK-1 and Aldolase A.^8^ However, the biological function and underlying mechanism of JOSD2 in lung cancer yet remains to be fully understood. Our findings showed that JOSD2 is negatively associated with NSCLC overall survival, deepening the current understanding on MJD subfamily members. We also identified histidine residue 125 (H125) as a new catalytic site crucial for the deubiquitinating activity of JOSD2, in addition to previously reported cysteine 24. This newly identified catalytic site can serve as a potential target for developing inhibitors against JOSD2 catalytic activity.

In attempts to elucidate the underlying mechanisms of tumor-promoting effect of JOSD2, we found that overexpression of JOSD2 removes K6-linked polyubiquitination of LKB1, and the deubiquitination of K6 linkage of LKB1 leads to disruption of LKB1/STRAD/MO25 complex integrity, thus inhibits kinase activity of LKB1 and downstream AMPK signaling pathway, so as to induce cell proliferation. The regulation of LKB1 activity by ubiquitination was also observed in a recent study, whereby Lee et al. showed that K63 linkage of LKB1 mediated by E3 ligase Skp2 dictates the complex integrity and kinase activity of LKB1.^18^ However, the LKB1 kinase activity maintained by K63 linkage confers LKB1 with the oncogenic activity in hepatocellular carcinoma (HCC) models.^18^ In our study, we showed that SKP2 was not responsible for the LKB1 K6-linked ubiquitination (Supplementary Fig. S4e), suggesting that the K6 linkage on LKB1 utilized some other E3 ubiquitin ligase(s) instead of SKP2. And such K6 linkage primed LKB1 kinase activity contributed to its tumor suppressive, but not promoting function in NSCLC, this is consistent with previous finding that LKB1 functions as a tumor suppressor in NSCLC and other cancers such as sporadic endometrial cancers,^38^ neuroendocrine lung cancers,^39^ pancreatic and biliary neoplasms.^40^ The seemingly contradictory roles of K63- and K6-linkages on LKB1 function in cancer malignancy may be explained by the differential tumor models. In Lee’s study, K63 linkage on LKB1 is enhanced by HRAS mutations, as the E3 ligase activity of Skp2 is greatly induced by HRAS mutations in HCC models.^18^ Whereas, our study focused on NSCLC, a different cancer type with a distinct cellular context harboring frequent KRAS mutations (30.9%);^41^ more importantly, the tumor suppressive function of LKB1 in NSCLC is well established and has been confirmed both in experimental models and NSCLC patients. Therefore, differential ubiquitin linkages may show great diversity in the impact on a particular protein substrate and its biological functions based on the cellular context.

In our study, we observed that the simultaneous introduction of JOSD2 and LKB1 in LKB1-null cells led to a higher level of cell growth than the control group, even though LKB1 alone has been known to suppress this. In fact, besides its traditional role as a tumor suppressor, LKB1 was found to promote cancer through the AKT-FoxO3a pathway.^13,14^ It is possible that introducing JOSD2 exogenously and restoring LKB1 in NCI-H460 cells - which lack LKB1 but have overactivated AKT^15,16^-might stimulate more aggressive tumor growth. CAL-101 (Idelalisib), an FDA-approved PI3K inhibitor that is known to suppress the AKT-FoxO3a pathway,^42^ was introduced and almost completely abolish the accelerated cell proliferation instigated by JOSD2 + LKB1, implicating the involvement of AKT signaling (Supplementary Fig. S11). It should also be noted that overexpression of LKB1 in NCI-H460 cells may activate other signaling pathways or increase the levels of other proteins, among which probably includes the potential substrates for JOSD2, which merit further exploration in the future.

While a number of different polyubiquitin chains have been demonstrated to modulate distinct cell signaling pathways, our knowledge on the cellular functions of K6 linkage remains elusive. Parkin was reported to be ubiquitinated by K6 linkage and the modification hinders the process of mitophagy.^43^ Another study identified Mitofusin-2 as a substrate of K6 linkage using a K6-specific affirmer.^44^ The tumor suppressor gene, BRCA1, is known to be responsible for assembling K6 linkage through its E3 ubiquitin ligase activity,^45^ suggesting that K6 linkage may be essential in BRCA1-mediated tumor suppression. HECT-type ubiquitin ligase HUWE1,^46^ also mediates the K6 linkage after valosin-containing protein (VCP) inhibition. Interestingly, VCP inhibitor CB-5083, which increases the K6-linked polyubiquitination, is currently under investigation as an approach to target the ubiquitin proteasome system in different types of hematological and solid malignancies.^47,48^ These studies collectively support the notion that K6-linked polyubiquitination may serve as an important signal for tumor suppression. Herein, our finding is consistent with these studies as we found that K6-linked polyubiquitination is critical for the tumor suppressive function of LKB1. Furthermore, the findings that JOSD2 specifically removes K6 linkage of LKB1 to suppress its kinase activity also deepened our understanding on the biological functions of K6 linkage in signaling transduction, particularly in LKB1 related pathways.

Our study identified the first JOSD2 inhibitor HY041004, which exhibited potent anticancer capacities against NSCLC cells both in vitro and in vivo. This compound was discovered by a cell-free ubiquitin-AMC assay using rhJOSD2, and its inhibition against JOSD2 catalytic activity was verified at cellular levels through the following aspects: the attenuation of K6 linkage removal, the restoration of LKB1-STRAD-MO25 complex integrity and the reactivation of LKB1 kinase activity. These findings not only provide a new inhibitor against DUB(s) with the potential to be developed as a candidate compound for cancer therapy, but also implicated that the pharmacological inhibition of JOSD2 by small molecular compound(s) could reactivate LKB1 by modulating its K6 linkage, thus further verified this newly-established JOSD2-LKB1 axis which was critical for NSCLC proliferation. However, in addition to LKB1, the previously reported substrate of JOSD2, such as PHGDH,^7,8^ could not be excluded in the process that HY041004 arrested NSCLC proliferation, as this compound also displayed some activities in LKB1-null cells A549 and NCI-H460, with a less robust extent compared to those LKB1-WT cells. In addition to that, we also noticed that inhibition of JOSD2 influenced the phosphorylation levels of AKT, MEK, and ERK (integral components of the AKT and MAPK pathways), the protein level of Occludin (an important component of tight junction pathway) etc. (Supplementary Fig. S3c). These observations could likely be attributed to the suppression of cell proliferation and the induction of apoptosis following JOSD2 inhibition and the regulatory mechanisms impacting these pathways by JOSD2 demand further clarification in future studies.

Our findings demonstrated the role of JOSD2 as an oncogene in promoting growth of NSCLC both in vitro and in vivo. Mechanistically, JOSD2-mediated removal of K6-linked polyubiquitination on LKB1 lysine residues 191, 269 and 423 resulted in disruption of LKB1-STRAD-MO25 complex integrity and inhibition of LKB1 kinase activity in NSCLC. Our study not only provides great insight into how LKB1 kinase activity is prohibited in NSCLC, but also offers new therapeutic strategies for targeting JOSD2 in NSCLC patients with JOSD2 inhibitors.

## Materials and methods

### Cell culture

All cell lines were purchased from the Cell Bank of the Chinese Academy of Sciences (Shanghai, China) and CoBioer Biosciences Co., Ltd (Nanjing, China). All cells were cultured at 37 °C in 5% CO_2_. Authentication of the cell lines was confirmed by short tandem repeating profiling every 6 months. All cell lines were routinely tested and confirmed to be free of mycoplasma.

### Antibodies and reagents

Antibodies against LKB1 (#3050), p-LKB1 (Ser428, #3482), Ki-67 (#9449), AMPKα (#5832), p-AMPKα (Thr172, #2535), p-MEK (#9154), MEK (#4694), p-ERK (#4370), ERK (#4695), YAP (#14074), TAZ (#8418), Cleaved-PARP (#5625), PARP (#9532), Occludin (#91131), p-AKT (#4060), AKT (#4685) and MO25 (#2716) were purchased from Cell Signaling Technology. Antibodies against LKB1 (SC-32245), GST (SC-138) and Ubiquitin (SC-8017) were purchased from Santa Cruz Biotechnology. Antibodies against JOSD2 (SAB2103354) were purchased from Sigma-Aldrich. Antibodies against JOSD2 (orb184482) were purchased from Biorbyt. Antibodies against GAPDH (db106) and HA (db2603) were purchased from diagbio Biosciences. Antibodies against Flag (#A00187) and Myc (#A00704-100) were purchased from Gen-Script. Anti-DYKDDDDK IP resin (#L00425) were purchased from Gen-Script and the anti-HA affinity gel (#B23302) was purchased from BioTools.

### Gene transfection and viral infection

To transfect cells with exogenous plasmids, Jet PRIME (Polyplus, Strasbourg, France; #114-15) was used according to the manufacturer’s instructions. For viral infection, cells were with lentiviruses packaged indicated plasmids for 24 h and supplemented with fresh culture medium. After 3 days, these cells were selected by treating with the culture medium containing 5 μg/mL puromycin for 24 h, then those surviving cells were used to perform the subsequent experiments.

### Cell proliferation assay and colony formation assay

For cell proliferation assay, cells infected with lentiviruses packaged indicated plasmids were seeded indicated number cells per well in 96-well plates. Cell number (absorbance) was then estimated by the sulforhodamine B (SRB) (Sigma-Aldrich, #230162) assay and the absorbance of the individual wells was determined at the optical density of 515 nm. For colony formation assay, cells infected with lentiviruses packaged indicated plasmids were seeded indicated number cells per well in 6-well plates. Two weeks later, cells were stained with SRB and the numbers of colonies having more than 50 cells were counted.

### Immunoblotting analysis and immunoprecipitation

For immunoblotting analysis, cells were lysed in RIPA lysis buffer (50 mM Tris-Base, 150 mM NaCl, 5 mM EDTA, 1% NP40, 0.1% SDS, pH 7.4) supplemented with protease inhibitor cocktail (Selleck, S7380). The protein concentrations of cell lysates were measured by using a BCA Protein Quantification Kit (YEASEN, 20201ES86). Lysates were boiled for 10 minutes and separated using SDS-PAGE. For immunoprecipitation assay, cells were lysed in RIPA lysis buffer directly, or 4% SDS lysis buffer (4% SDS, 150 mM NaCl, 50 mM Triethylamine, pH 7.4) followed by sonication. Clarified cell lysates were incubated with indicated antibody overnight at 4 °C, followed by incubation with Protein A/G beads (Biotool, L00425) at 4 °C for 2 h or overnight. The immunocomplexes were washed five times with RIPA lysis buffer and then subjected to immunoblotting with indicated antibodies.

### In vivo deubiquitination assay

For the in vivo deubiquitination assay, 293T or 293FT cells were harvested 48 h after transfection with indicated plasmids. For denaturing, lysates were heated at 95 °C for 10 min in the presence of 4% SDS lysis buffer, followed by tenfold dilution with RIPA lysis buffer and sonication. The cell lysates were then incubated with Flag beads for 6 h at 4 °C and then beads were washed with RIPA lysis buffer five times and subjected to immunoblotting analysis.

### In vitro kinase assay

Endogenous LKB1 or exogenous LKB1-Flag was immunoprecipitated from cells by antibodies against LKB1 or Flag, and then immunoprecipitates were incubated with recombinant His-AMPKα_1–312_ for 30 min at 37 °C in 80 μl of reaction buffer (25 mM Tris-HCl, 10 mM MgCl_2_, 2 mM DTT, 0.5 mM ATP, pH 7.5). After incubation, proteins were eluted in SDS-sample buffer and subjected to immunoblotting analysis. LKB1 kinase activity was determined by measuring Thr172 phosphorylation of recombinant AMPKα_1–312_.

### Statistical analysis

Statistical analysis was performed using GraphPad Prism software (GraphPad Software Inc.). The values were presented as means ± standard deviation (SD) or means ± standard error of mean (SEM). Two-tailed unpaired Student *t* tests and one-way ANOVA tests were used for statistical analysis. Results were considered significant when *P* < 0.05 (*P < 0.05, **P < 0.01 and ***P < 0.001).

### Study approval

All animal studies were performed in specific-pathogen-free (SPF) conditions and approved by the Animal Research Committee at Zhejiang University with ethical approval number IACUC-s19-043. The patient-derived xenografted study was approved by the Ethics Committee of the Hangzhou First People’s Hospital (Ethics Approval License: 2018-122). Informed patient consent was obtained.

### Supplementary information

Supplementary Material

Raw Data of Statistical Analysis

## Data Availability

The raw data of RNA sequencing have been deposited (accession number: GSE198415), to review GEO accession GSE198415, go to the following site, and enter token efypcqayjfmlhul into the box: https://www.ncbi.nlm.nih.gov/geo/query/acc.cgi?acc=GSE198415. The mass spectrometry proteomics data have been deposited to the ProteomeXchange Consortium via the Proteomics Identification Database (PRIDE),^49^ partner repository with the dataset identifier PXD046139 (Username: reviewer_pxd046139@ebi.ac.uk, Password: FqSPFwKY). All the plasmids used in this manuscript will be available from the corresponding author upon reasonable request.
