# Supplementary material for: Josephin domain containing 2 (JOSD2) promotes lung cancer by inhibiting LKB1 (Liver kinase B1) activity
Source: Signal Transduct Target Ther. 2024 Jan 5;9:11. doi: 10.1038/s41392-023-01706-y (PMC10766984; doi:10.1038/s41392-023-01706-y)
Supplement: Supplementary file 1 — Supplementary Material [file 41392_2023_1706_MOESM1_ESM.docx]

Supplementary Materials for

**Josephin domain containing 2 (JOSD2) promotes lung cancer by inhibiting LKB1 (Liver kinase B1) activity**

Running title: JOSD2 promotes lung cancer by inhibiting LKB1 activity

Tao Yuan^1,#^, Chenming Zeng^2,#^, Jiawei Liu^3,#^, Chenxi Zhao^1^, Fujing Ge^1^, Yuekang Li^1^, Meijia Qian^1^, Jiamin Du^1^, Weihua Wang^1^, Yonghao Li^1^, Yue Liu^1^, Xiaoyang Dai^4^, Jianya Zhou^5^, Xueqin Chen^6^, Shenglin Ma^6^, Hong Zhu^1,7,*^, Qiaojun He^1, 8,*^, Bo Yang^1,*^

^#^: These authors contribute equally to this study.

Correspondence to: hongzhu@zju.edu.cn, qiaojunhe@zju.edu.cn, yang924@zju.edu.cn.

**This PDF file includes:**

Materials and Methods

Figures. S1 to S11

Tables S1 to S9

**Other Supplementary Materials for this manuscript include the following:**

Raw data of Statistical Analysis

**Materials and Methods**

Cell culture

PC-9, NCI-H2228, NCI-H2347, NCI-H1650, NCI-H23, NCI-H1395 and NCI-H1373 are LUAD cell lines, NCI-H226 and Calu-1 are LUSC cell lines, NCI-H292 is mucoepidermoid pulmonary carcinoma, NCI-H1299, NCI-H358, NCI-H23 and NCI-H838 are from metastases. All these NSCLC cell lines were cultured in RPMI 1640 (Gibico) containing 10% fetal bovine serum (FBS) (Gibco BRL, Grand Island, NY, USA) except that A549 cells was maintained in F12 medium (Gibico) supplemented with 10% FBS. 293T and 293FT cells were cultured in Dulbecco’s modified Eagle’s medium (DMEM) (Gibico) supplemented with 10% FBS.

**Data mining**

The correlation of human DUBs mRNA levels with survival possibilities of NSCLC patients were analyzed using the TCGA database. Gene expression of JOSD2, JOSD1, Ataxin-3 and Ataxin-3L in human lung cancer and normal tissues were compared using TCGA database. The correlation of JOSD2 mRNA levels with prognosis of NSCLC patients were analyzed using the SurvExpress database ^1^.

**GST pull down assay**

For GST pull down assay, GST fusion protein was bound to GSH Sepharose (Sangon Biotech, C600031) at 4℃ for 2 hours. The GSH Sepharose were washed with PBS twice and incubated with indicated cell lysates at 4℃ for another 1 hour. After washing with RIPA lysis buffer for three times, the bound proteins were subjected to immunoblotting with indicated antibodies. The specific antibodies and reagents used in this study were detailed in key resources materials.

**Immunohistochemistry (IHC) assay**

80 lung tumor samples of patients with lung adenocarcinomas were collected from the Affiliated Hangzhou Cancer’s Hospital, Zhejiang University School of Medicine (Hangzhou, China) and made into human lung cancer tissue array, the patient-derived tumor study was approved by the Ethics Committee of the Hangzhou Cancer’s Hospital (Ethics Approval License: 2020-023-01). Tissue slides were first de-paraffinized and immersed in PBS for 10 minutes. Then the slides were heated in microwave oven for 15 minutes in Citrate Antigen Retrieval buffer. After cooling to room temperature, the slides were incubated with 3% H_2_O_2_ to block endogenous peroxidases followed by incubation with 10% goat serum for 30 minutes to block non-specific staining. The slides were subsequently incubated with anti-JOSD2 (Biorbyt, orb184482) (For Ki-67 staining, the slides were subsequently incubated with anti-Ki67 (Cell Signaling Technology, #9449) diluted at 1:50 overnight at 4°C and indicated second antibody for 30 minutes. Lastly, the slides were treated with DAB for 3 minutes and rinsed off in deionized water to terminate DAB reaction. The evaluation of the IHC staining was performed by pathologist who is blind from the stage information of these patients, IHC score was evaluated by multiplication of positive staining proportions (1 score, <25%; 2 score, 25%-50%; 3 score, 50%-75%; 4 score, 75%-100%) and intensity of protein expressions (1 score, weak staining; 2 score, moderate staining; 3 score, high staining).

**Plasmids**

JOSD2 shRNA plasmids were cloned into pLKO.1/U6 constructs (Addgene). JOSD2 and LKB1 expression plasmids were cloned into pCDH-EF1-Puro constructs (System Biosciences). The mutations of JOSD2 and LKB1 were generated by site-directed mutagenesis and verified by sequencing. Sequences for gene-specific shRNA targeting DUBs were listed in supplementary Table S8, and primers sequences for site-directed mutagenesis of all the lysines at LKB1 with arginines (LKB1-KR) were listed in supplementary Table S9.

**NCI-H358 xenograft studies**

NCI-H358 cells were infected with lentiviruses packaged Scramble control or JOSD2 shRNA for 24 h and supplemented with fresh culture medium. After 3 days, these cells were selected by treating with the culture medium containing 5 μg/mL puromycin for 24 h. Then these cells were subcutaneously transplanted into female NSG mice (4-5 weeks) in a density of 5×10^6^ cells in 200 μL medium per mice. The tumor volume was measured by a caliper in the indicated times as soon as the tumor reached a palpable size. Calculation was carried out as follows: tumor volume = (length × width^2^) / 2.

**PC-9 xenograft studies**

PC-9 cells were infected with lentiviruses packaged Vector/JOSD2-WT/C24A for 24 h and supplemented with fresh culture medium. After 3 days, these cells were selected by treating with the culture medium containing 5 μg/mL puromycin for 24 h. Then these cells were subcutaneously transplanted into female NSG mice (4-5 weeks) in a density of 3×10^6^ cells in 200 μL medium per mice. The tumor volume was measured by a caliper in the indicated times as soon as the tumor reached a palpable size.

**NCI-H1299 xenograft studies**

Female BALB/c-Nude mice (4-5 weeks) were subcutaneously transplanted with 1 mm^3^ NCI-H1299 xenograft tumor mass. Once tumors reached approximately 50 mm^3^ in volume, mice were randomly assigned to scramble control or JOSD2 shRNA intra-tumor injection treatment. For the intratumor injection, a mice received an intratumorally injection of 5 ×10^6^ lentiviruses in 20 μL sterile saline solution every two days. The tumor volume was measured by a caliper in the indicated times as soon as the tumor reached a palpable size.

**NCI-H460 xenograft studies**

NCI-H460 cells were infected with lentiviruses packaged Vector/LKB1-WT/3KR for 24 h and supplemented with fresh culture medium. After 3 days, these cells were selected by treating with the culture medium containing 5 μg/mL puromycin for 24 h. Then these cells were infected with lentiviruses packaged Vector/JOSD2 for 24 h. After 72 h, these cells were subcutaneously transplanted into female BALB/c-Nude mice (4-5 weeks) in a density of 5×10^6^ cells in 200 μL medium per mice. The tumor volume was measured by a caliper in the indicated times as soon as the tumor reached a palpable size.

**RNA sequencing analysis**

NCI-H1299 cells were infected with lentiviruses of scramble control or JOSD2 shRNA for 24 h and supplemented with fresh medium cultured for another 3 days. Cells were washed with RNA-free PBS three times and Using TRIzol (Invitrogen) to extract total RNA of these cells. The concentration and integrity of RNA were assessed using Bioanalyzer 2100 and RNA 6000 Nano LabChip Kit (Agilent, CA, USA). The RNA fragments were reverse-transcribed to create the cDNA by SuperScript™ II Reverse Transcriptase (Invitrogen, cat. 1896649, USA). RNA sequencing analysis was performed on the illumina Novaseq™ 6000 (LC-Bio Technology CO., Ltd., Hangzhou, China) according to the recommended protocols. RNA-Seq reads were then aligned to *Homo sapiens* GRCh38 with STAR and genome-wide transcript counting was performed by StringTie to generate a matrix of fragments per kilobase of exon per million fragments mapped (RPKM). All the raw data of RNA sequencing have been deposited (accession number: GSE198415), to review GEO accession GSE198415, go to the following site, and enter token efypcqayjfmlhul into the box: https://www.ncbi.nlm.nih.gov/geo/query/acc.cgi? acc=GSE198415.

***In vitro* ubiquitin-AMC assay**

For the *in vitro* ubiquitin-AMC assay, purified proteins were incubated in reaction buffer (50 mM Tris-HCl, 5 mM MgCl_2_, 2 mM DTT, 2 mM ATP, pH 7.5) at 37°C for 1 hour. Reactions were initiated by adding 2 μM ubiquitin-AMC (Boston Biochem, #U-550). Fluorescence levels were measured continuously at 25°C using Tecan Spark Microplate Reader at an excitation wavelength of 345 nm and an emission wavelength of 445 nm. For JOSD2 inhibitors screening, the source of the inhibitors were: ① 15 reported DUB inhibitors, ②31 clinically used drugs, ③53 inhibitors with new structure synthesized by our collaborator Prof. Jiawei Liu who is one of the co-first authors of this study. ① and ② was purchased from Target Mol (USA). These compounds (at concentration of 2 μM) were incubated with GST-JOSD2 protein at 37°C for 30 minutes and then subjected to *in vitro* ubiquitin-AMC assay. PR-619 (Abcam, ab144641), a broad inhibitor of DUB, was introduced as a positive control^2^.

***In vitro* deubiquitination assay**

For the *in vitro* deubiquitination assay, purified GST-JOSD2 or GST protein was incubated with HA-Ub K6 or wild-type HA-Ub ubiquitinated LKB1-Flag purified by Flag beads. After reacting in reaction buffer (50 mM Tris-HCl, 5 mM MgCl_2_, 2 mM DTT, 2 mM ATP, pH 7.5) at 37°C for 1 hour, the Flag beads were washed with RIPA lysis buffer five times and subjected to immunoblotting analysis.

**Liquid chromatography-mass spectrometry (****LC-MS) analysis**

For LC-MS analysis of the ubiquitination sites on LKB1, LKB1-Flag and HA-Ub K6 were transfected into 293FT cells for 48 hours before harvest. Cells were collected and lysed in the presence of 4% SDS lysis buffer, followed by tenfold dilution with RIPA lysis buffer and sonication. The cell lysates were then incubated with Flag beads for 6 hours at 4°C and then beads were washed with RIPA lysis buffer for five times. The immunoprecipitated Flag-LKB1 complexes was separated using SDS-PAGE, when the immunoprecipitated complexes samples enter the separation gel about 3-4 cm, stopping the electrophoresis and putting the gel in Coomath reagent for 10-15 min, then washing the gel with the Coomath elute until the bands of target protein is clearly visible. Subsequently, the bands (~1 cm^2^) were excised from the gel with a clean pipette tip and subjected to trypsin digestion and LC-MS analysis. The identification of ubiquitin remnant-containing peptides is achieved by detecting a gly-gly adduct (114 Da) on the modified lysine residues. For LC-MS analysis of the candidate JOSD2-interacting proteins, Vector or JOSD2-HA were transfected into 293FT cells for 48 h before harvest and cells were collected and lysed in the presence of 1% NP40 lysis buffer, followed by tenfold dilution with RIPA lysis buffer. The cell lysates of two group were then incubated with HA beads for 6 hours at 4°C and then beads were washed with RIPA lysis buffer for five times. The immunoprecipitated JOSD2-HA complexes of two group were subjected to trypsin digestion and LC-MS analysis. To exclude the non-specific interacting proteins, we used the blank group that 293FT cells were transfected with Vector, but not JOSD2-HA, as a negative control and excluded the proteins identified in both two groups, moreover, those proteins identified by only one unique peptide also be excluded when we analyzed LC-MS data by KEGG enrichment analysis (KEGG enrichment analysis was conducted using the R package clusterProfiler (version 4.7.1) with default parameters).

**Affinity purification mass spectrometry (AP-MS) analysis**

293T cells were infected with lentiviruses packaged Vector or JOSD2-SBP (Streptavidin binding peptide) for 24 h and supplemented with fresh culture medium. After 3 days, these cells were selected by treating with the culture medium containing 5 μg/mL puromycin for another 3 days, therefore obtained 293T cell stable overexpression of Vector/JOSD2-SBP. These cells were collected and lysed in the presence of 1% NP40 lysis buffer, followed by tenfold dilution with RIPA lysis buffer. The cell lysates of two group were then incubated with Streptavidin Magnetic Beads (MedChemExpress, HY-K0208) for 6 hours at 4°C and then beads were washed with RIPA lysis buffer for 3 times and 1×PBS for 3 times. The immunoprecipitated JOSD2-SBP complexes were subjected to trypsin digestion and MS analysis. Similarly, to exclude the non-specific interacting proteins, we used the blank group that 293T cells infected with lentiviruses packaged Vector, but not JOSD2-SBP, as a negative control and excluded the proteins identified in both two groups when we analyzed.

The LC-MS analysis and AP-MS analysis were performed by Jingjie PTM BioLab (Hangzhou). Co. Inc, and the operation procedures were carried out according to the instructions provided by the company. In brief:

**Trypsin Digestion:** Take gel piece for example: The gel pieces were destined in 50 mM NH_4_HCO_3_ of 50% acetonitrile (v/v) until clarified. Gel pieces were dehydrated with 100 μl 100% acetonitrile for 5 min, rehydrated with 10 mM dithiothreitol at 56℃ for 60 min, dehydrated with 100% acetonitrile again, and rehydrated with 55 mM iodoacetamide. The gel pieces were incubated at room temperature in the dark for 45 minutes, washed with 50 mM NH4HCO3 and dehydrated with 100% acetonitrile, rehydrated with 10 ng/μl trypsin in 50 mM NH_4_HCO_3_ on ice for 1 h, subsequently digested with trypsin at 37℃ overnight. The peptides were extracted by 50% acetonitrile/5% formic acid, followed by 100% acetonitrile. And dried to completion and resuspended in 2% acetonitrile /0.1% formic acid.

**LC-MS/MS Analysis:** The tryptic peptides were dissolved in 0.1% formic acid (solvent A) and directly loaded onto a self-made reverse-phase analysis column (15 cm long, 75 μm i.d.). On the EASY-nLC 1000 UPLC system, at a constant flow rate of 400 nl/min, the solvent B (0.1% formic acid, 98% acetonitrile) was increased from 6% to 23% in 16 minutes, from 23% to 35% in 8 minutes, to 80% in 3 minutes, and then remained at 80% in the last 3 minutes. The peptides were passed through an NSI source and then coupled online to the UPLC by tandem mass spectrometry (MS/MS) in QQ Exactive^TM^ Plus (Thermo). The applied electrospray voltage is 2.0 kV. The m/z scan ranges from 350 to 1800 at full scanning, and complete peptides were detected at 70,000 resolutions at Orbitrap. The peptides were then selected for mass spectrometry using NCE setting as 28, and detected at a resolution of 17500 in Orbitrap. A data-dependent process that alternated 20 MS/MS scans after one MS scan with a dynamic exclusion time of 15.0 s. Automatic gain control (AGC) was set as 5E4.

**Data Processing:** Proteome Discoverer 1.3 was used to process the obtained MS/MS data. Tandem mass spectra were searched against Homo Sapiens (SwissProt). Mascot was used as the tandem mass spectrometry peptide identification search algorithms, the MS/MS data were evaluated by using a target-decoy protein sequence search library strategy and the analysis was performed to achieve the False Discovery Rate (FDR) less than 1%. Trypsin/P was specified as cleavage enzyme, allowing for up to 2 missing cleavages. The mass error was set to 10 ppm for the precursor ion and 0.02 Da for the fragment ions. Carbamidomethyl on Cys were specified as fixed modification and oxidation on Met was specified as variable modification. The peptide confidence was set at high and the peptide ion score was set > 20.

**Patient-derived xenograft (****PDX) studies**

PDXs were established in our lab using female BALB/c-Nude mice (4-5 weeks) implanted with tumor tissues from patients undergoing surgeries. These PDX models and PDC models in our study have been demonstrated harboring the wild-type LKB1 by sequencing. Once tumors reached approximately 50 mm^3^ in volume, mice were randomly assigned to scramble control or JOSD2 shRNA intra-tumor injection treatment. For the intratumor injection, a mice received an intratumorally injection of 5 ×10^6^ lentiviruses in 20 μL sterile saline solution every two days.

**Synthesis and production of HY041004 (3-(3-chloro-5-fluorobenzyl)-3H-[1,2,3] triazolo[4,5-g]isoquinoline-4,9-dione)**

The synthesis of HY041004 was accomplished through a three-step process ^3^. Initially, a magnetically stirring solution of 5-hydroxylisoquinoline (50.0 g, 34.4 mmol) in 1000 mL MeCN was added slowly to NaCO_3_ (180.0 g) in water (500.0 mL) at 0 °C (ice-water bath) under an atmosphere of nitrogen. 326.0 g (758.8 mmol) of (bis(trifluoroacetoxy)iodo) benzene was then added carefully. The mixture was then stirred at 0 °C for 30 min and at room temperature for 1 h. The mixture was extracted twice with methyl tert-butyl ether. The combined organic extracts were washed with brine and dried (MgSO_4_). The solvent was removed under vacuum and purification by silica gel flash chromatography gave isoquinolinie-5,8-dione (20.0 g, 125.6 mmol) as a grey solid. Secondly, to a stirred solution of 3-chloro-5-fluorobenzyl bromide (82.1 g, 367.3 mmol) in DMF (300 mL), of sodium azide (28,7 g, 442.0 mmol) was added. The reaction mixture was stirred at 80 °C for 24 h in an oil bath and then extracted with methyl tert-butyl ether. The combined organic extracts were washed with brine and dried over MgSO_4_ and concentrated under reduced pressure to give 3-chloro-5-fluorobenzyl azide (112.0 g, 603.4 mmol) as yellow oil. Finally, to a stirred solution of isoquinolinie-5,8-dione (20.0 g, 125.0 mmol) in 200 mL of EtOAC, of 3-chloro-5-fluorobenzyl azide (26.0 g, 140.9 mmol) was added and the mixture was refluxed for 48 h at 80°C under a nitrogen atmosphere. The reaction mixture obtained was then passed through a 1 cm silica gel layer. After evaporation the solvent, the crude product was purified by flash column chromatography on silica gel using EtOAc: PE (1:3) as an eluent to give the target compound 3-(3-chloro-5-fluorobenzyl)-3H-[1,2,3]triazolo[4,5-g] isoquinoline-4,9-dione (HY041004, 5.5 g, 16.0 mmol, Yield: 12.8%) of as a pale white solid. m.p.: 172-174°C; 1H NMR (400 MHz, Chloroform-d) δ 9.49 (s, 1H), 9.18 (s, 1H), 8.13 (s, 1H), 7.30 (s, 1H), 7.19 – 7.05 (m, 2H), 5.98 (s, 2H). 13C NMR (101 MHz, Chloroform-d) δ 175.6, 175.0, 164.0, 157.2, 149.3, 145.3, 138.8, 138.0, 136.6, 136.1, 125.4, 124.7, 119.8, 117.4, 114.3, 52.8. HRMS (ESI): m/z [M+H]+calcd for C_16_H_9_ClFN_4_O_2_: 343.0393; found: 343.0395.

**Cellular thermal shift assay**

For cellular thermal shift assay, it was conducted as previously described ^4^. The NCI-H358 cells were incubated with DMSO or HY041004 for 30 minutes at 37°C and heated at different temperatures for 3 minutes. The cells were lysed using RIPA lysis buffer and the supernatants were analyzed by immunoblotting analysis.

**Antitumor activity of HY041004 *in vivo***

Female BALB/c-Nude mice (4-5 weeks) were subcutaneously transplanted with 1 mm^3^ NCI-H1299 xenograft tumor mass. Once tumors reached approximately 50 mm^3^ in volume, mice were randomly assigned to control or HY041004 treatment group. HY041004 was given by oral gavage twice a day (50 mg/kg or 100 mg/kg). The tumor volume was measured by a caliper every 2 days as soon as the tumor reached a palpable size. Relative tumor volume (RTV) was calculated by (RTV=TV_t_ / TV_0_, where TV_0_ is the tumor volume measured when starting treatment). The T/C value was calculated as RTV_treated_ / RTV_control_. The anti-tumor effect of HY041004 treatment was calculated as (1-Tumor Weight_treated_ / Tumor Weight_control_) × 100%.

**TUNEL assay**

Apoptosis in xenografted tumors was detected by a TUNEL assay kit (Beyotime Biotechnology, Shanghai, China), according to the manufacturer's protocols. Images were captured by a fluorescence microscope.

**Immunofluorescent assay**

A549 cells transfected with LKB1-WT/3KR were plated in fluorescent chamber slides and fixed with 4% paraformaldehyde at 4 ℃ for 1 h. The slides were washed by PBS for 10 min three times. Cells were permeabilized with blocking buffer (0.1% Triton-X100 in PBS) at 37 ℃ for 30 min, then incubated with anti-Flag antibody (dilution rate 1:100) at 4 ℃ overnight. The slides were washed by PBS for 10 min three times, and incubated with secondary antibodies with 1:1000 dilution rate at room temperature for 1 h avoiding light. Followed by PBS washing for 10 min three times and DAPI (1:2500 diluted in PBS) incubating at room temperature for 2 min. Ultimately, the slides were sealed with anti-fade reagent (#0100-01, SouthernBiotech, Birmingham, AL, USA) and observed by confocal microscope.


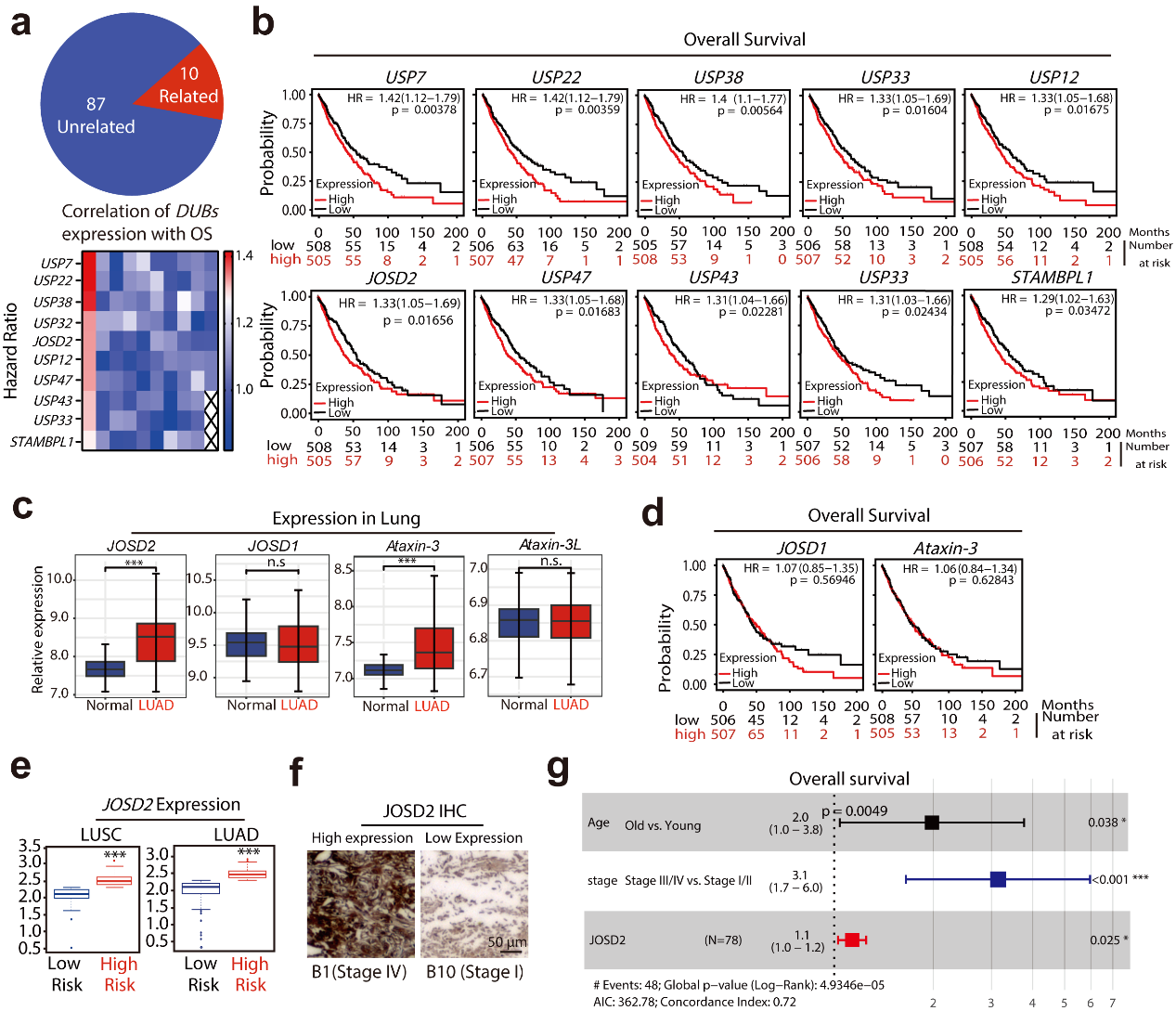


**Figure. S1. Screening of DUBs critical for NSCLC survival**

(**a**) Pie chart showing the distribution of 97 *DUBs* based on the correlation of their expression with OS of NSCLC patients (Top). Heatmap of hazard ratio depicting risk potential of 97 *DUBs* in NSCLC patients. 10 *DUBs* (*P* < 0.05) that are related with OS in NSCLC patients were listed (bottom). (**b**) Analysis of OS in NSCLC patients separated by expression of indicated *DUBs* (related with OS of NSCLC). (**c**) TCGA database analysis of *JOSD2*, *JOSD1*, *Ataxin-3* and *Ataxin-3L* expression in normal tissues and lung adenocarcinomas (LUADs). (**d**) TCGA database analysis of OS in NSCLC patients separated by expression of *JOSD1* or *Ataxin-3*. (**e**) SurvExpress analysis of *JOSD2* mRNA expression in squamous cell carcinomas (LUSCs) and LUADs separated by low risk or high risk. (**f**) Representative images of the high and low JOSD2 IHC scores in Fig. 1d (B1, Stage IV for high IHC score image; B10, Stage I for low score image, respectively). (**g**) Multivariate analysis the correlation of JOSD2 expression and overall survival of NSCLC patients, together with tumor stages and age on 80 NSCLC patients. n.s: *P* > 0.05; ****P* < 0.001.


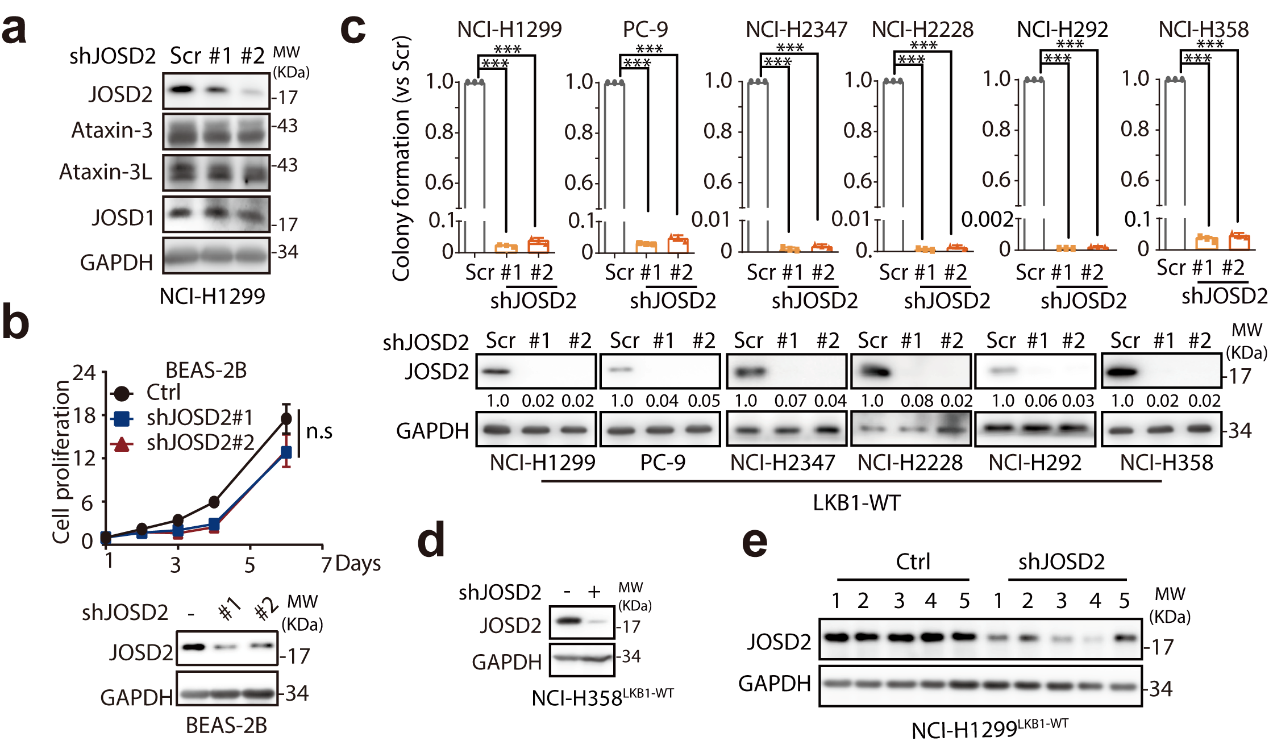


**Figure. S2. Depletion of JOSD2 poses slight inhibitory effects on Normal lung** **epithelial cells**

(**a**) Cell lysates of NCI-H1299 were subjected to IB. (**b**) Cell proliferation assay in BEAS-2B cells transfected with scramble control or JOSD2 shRNA (#1, #2) (left). BEAS-2B cells were infected with lentiviruses for 96 h, then these cells were seeded 1000 cells per well in 96-well plates, followed by SRB staining (means ± SD, n=3). IB analysis showing the knockdown efficiency of JOSD2 (right). (**c**) Statistical analysis of colony formation assay in Fig. 1f (top). IB analysis showing the knockdown efficiency of JOSD2 (bottom) (means ± SD, n=3). (**d**) IB analysis the knockdown efficiency of JOSD2 in NCI-H358 cells before subcutaneously transplant. (**e**) IB analysis showing the knockdown efficiency of JOSD2 in NCI-H1299 xenografted model. n.s: *P* > 0.05; ****P* < 0.001.


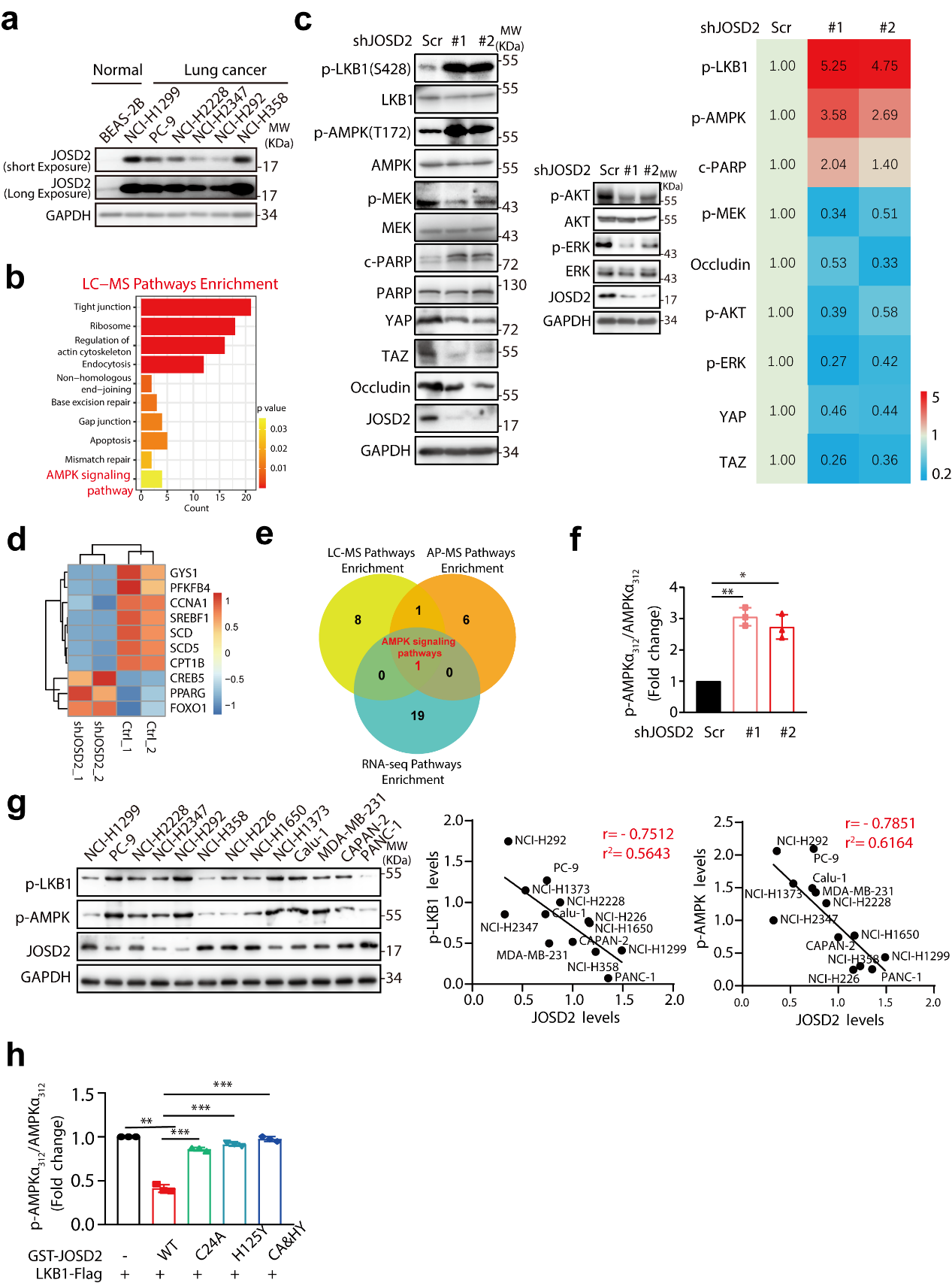


Figure. S3. LKB1/AMPK pathway is regulated by JOSD2

(**a**) Cell lysates of BEAS-2B, NCI-H1299, PC-9, NCI-H2228, NCI-H2347, NCI-H292 and NCI-H358 were subjected to IB. (**b**) IPs by HA beads from 293T cells transfected with Vector or JOSD2-HA for 48 h were subjected to LC-MS analysis of JOSD2-interacting proteins. (**c**) Cell lysates were subjected to IB and semi-quantitative analyses of the blots. (**d**) Representative differential expressed genes with logFC (Fold Change)>2 in the AMPK pathway. (**e**) AMPK pathway was enriched in these three methods of AP-MS, LC-MS and RNA sequencing. (**f**) Semi-quantitative analyses of the blots in Fig. 2e. (means ± SD, n=3). (**g**) Cell lysates of indicated cells were subjected to IB, and correlation analysis were performed between the expression levels of JOSD2, p-LKB1 and p-AMPK in several NSCLC cell lines. (**h**) Semi-quantitative analyses of the blots in Fig. 2i. (means ± SD, n=3). **P* < 0.05; ***P* < 0.01; ****P* < 0.001.


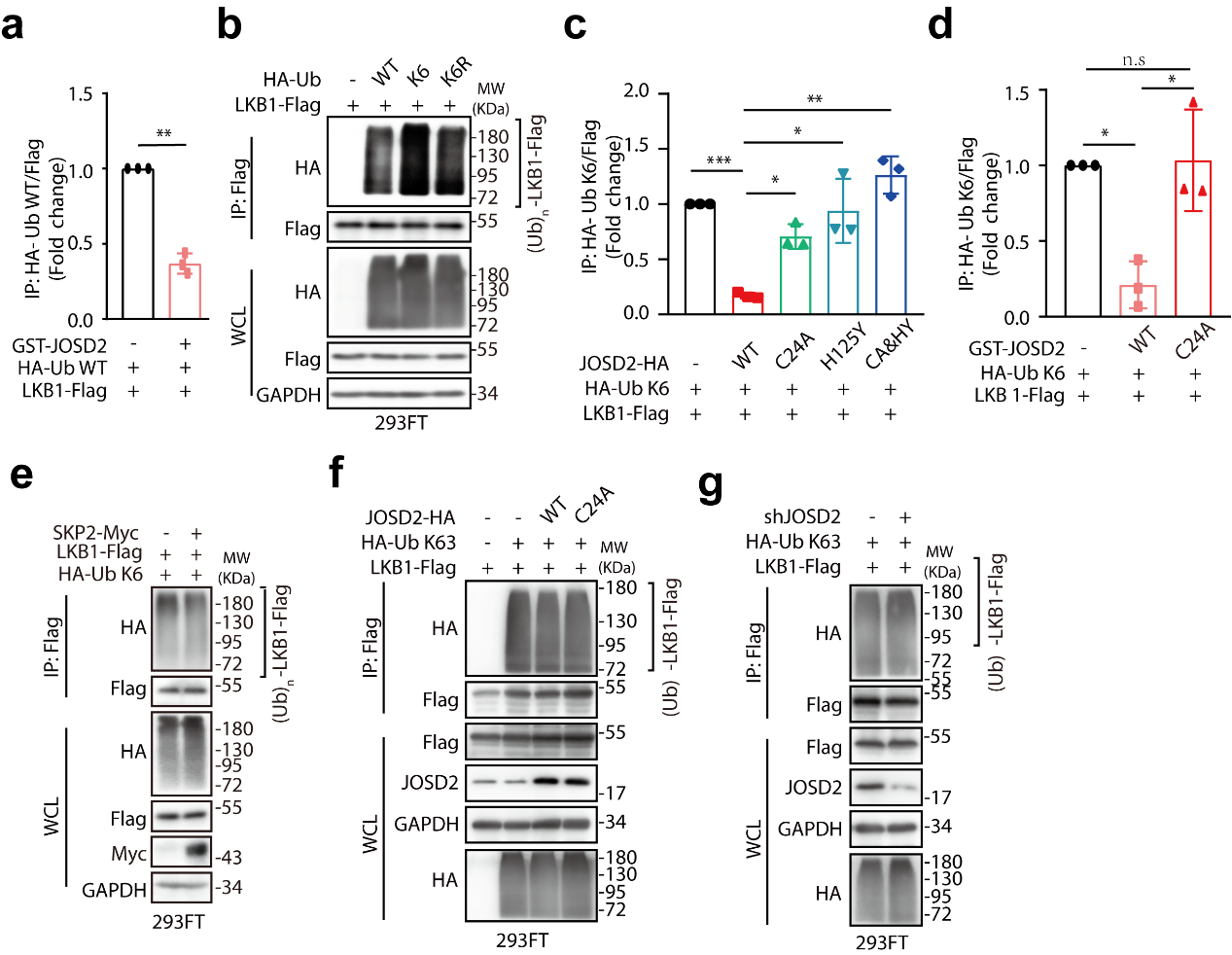


**Figure. S4. LKB1 mainly ubiquitinated through K6 linkage**

(**a**) Semi-quantitative analyses of the blots in Fig. 3e (means ± SD, n=3). (**b**) IPs by Flag beads from 293FT cells transfected with LKB1-Flag along with WT-ub or indicated ubiquitin mutants for 48 h were subjected to IB. (**c**) Semi-quantitative analyses of the blots in Fig. 3g (means ± SD, n=3). (**d**) Semi-quantitative analyses of the blots in Fig. 3k. (means ± SD, n=3). (**e**) IPs by Flag beads from 293FT cells transfected with LKB1-Flag, HA-ub K6 along with SKP2-Myc or not for 48 h were subjected to IB. (**f**) IPs by Flag beads from 293FT cells transfected with LKB1-Flag, HA-ub K63 along with JOSD2-WT or C24A for 48 h were subjected to IB. (**g**) 293FT cells infected with Scramble or JOSD2 shRNA were transfected with LKB1-Flag, HA-ub K63 for 48 h, and the cell lysates were subjected to IPs. n.s: *P* > 0.05; **P* < 0.05; ***P* < 0.01; ****P* < 0.001.


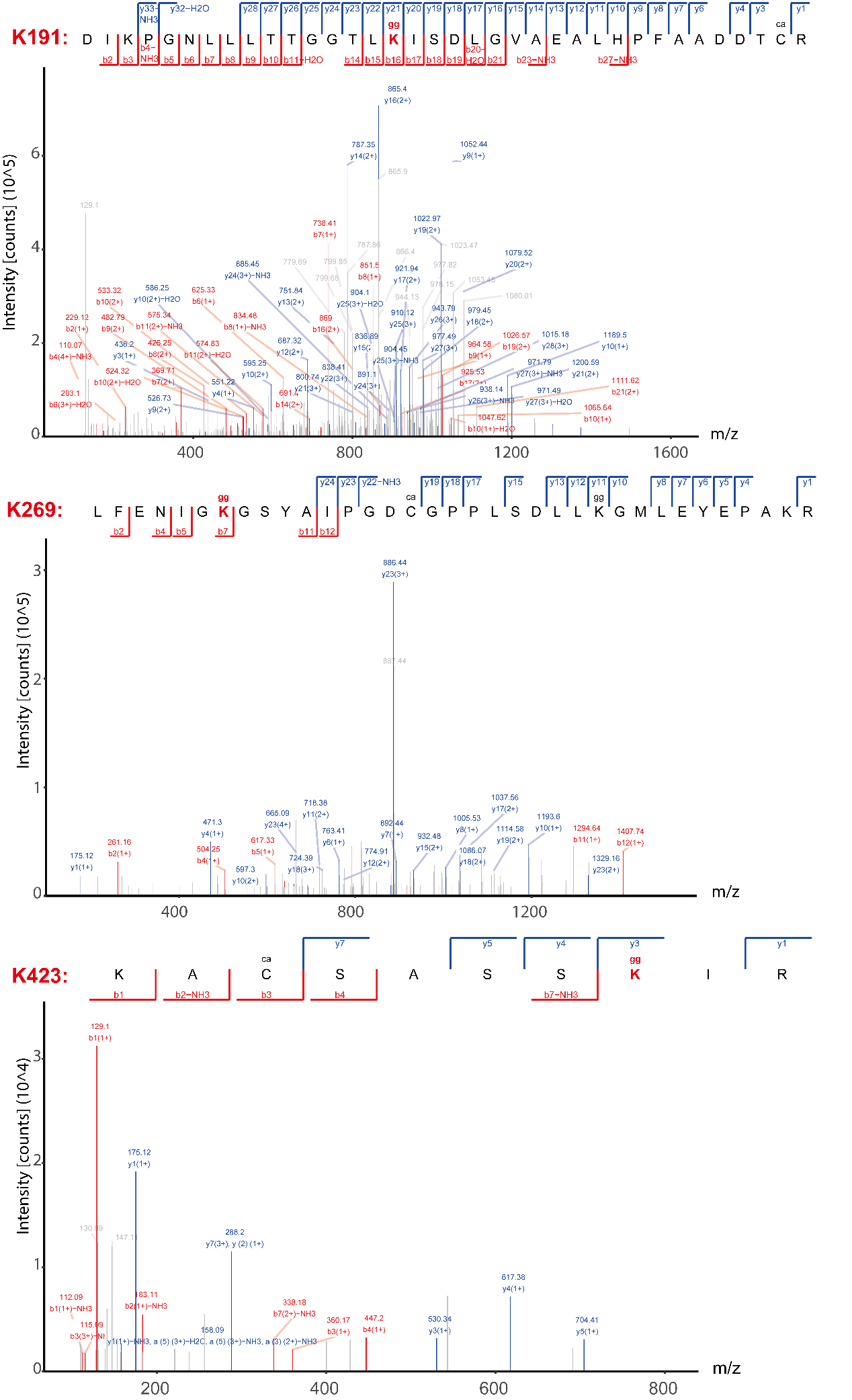


**Figure. S5. The mass spectrum of LKB1 lysine 191 (K191), lysine 269 (K269) and lysine 423 (K423) ubiquitination sites**

Identification of LKB1 lysine 191 (K191), lysine 269 (K269) and lysine 423 (K423) ubiquitination sites using mass spectrometry analysis. The identification of ubiquitin remnant-containing peptides is achieved by detecting a gly-gly adduct (114 Da) on the modified lysine residues.

**
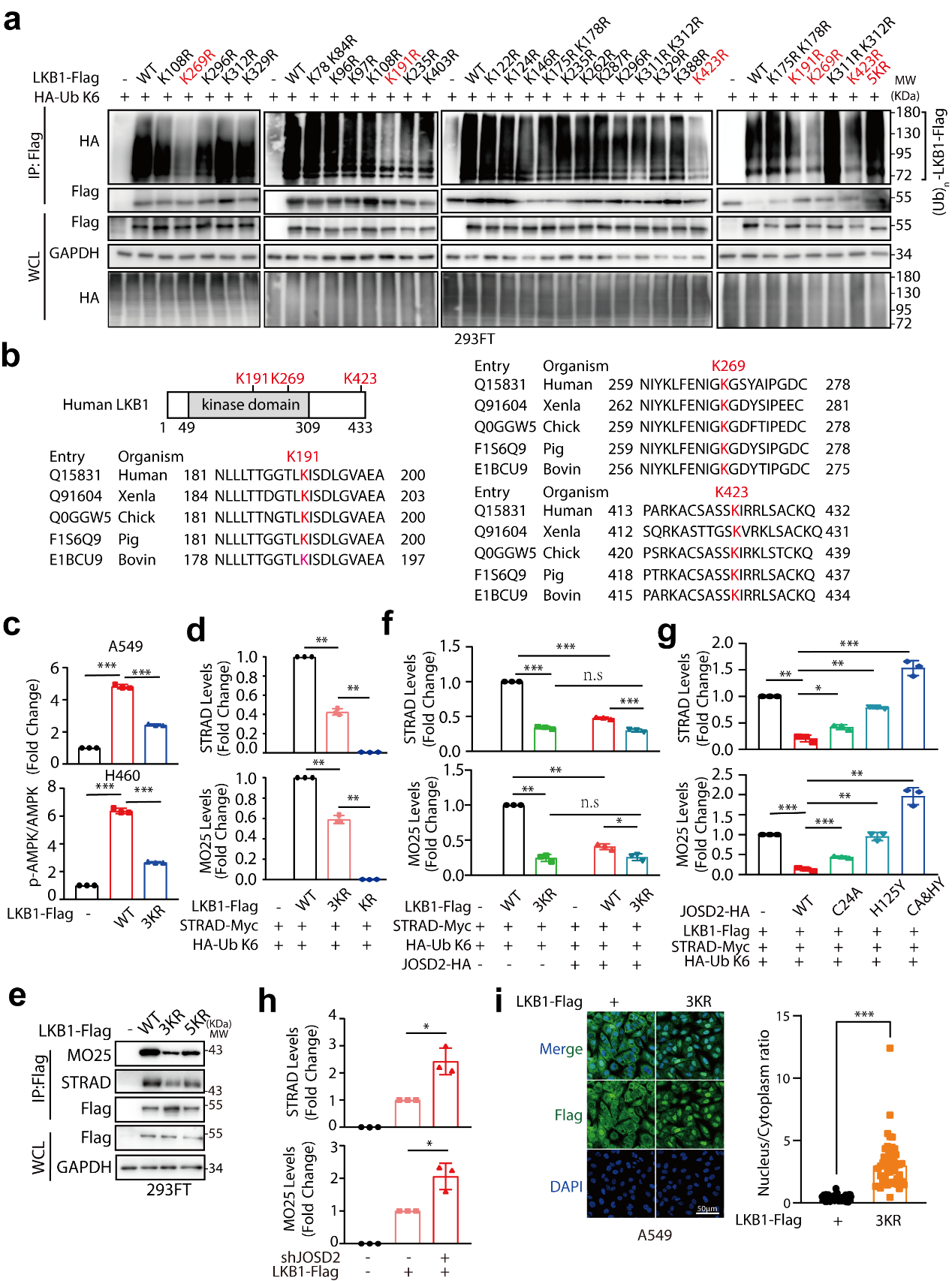
Figure. S6. Identification of LKB1 K6-linked polyubiquitination sites**

(**a**) IPs by Flag beads from 293FT cells transfected with indicated LKB1-Flag along with HA-ub K6 for 48 h were subjected to IB. (**b**) Schematic illustration of human LKB1 protein marked with three potential ubiquitination sites including K191, K269 and K423 (top). Sequence alignment of K191, K269 and K423 ubiquitination sites within LKB1 orthologues of different species (bottom). (**c**) Semi-quantitative analyses of the blots in Fig. 4d. (means ± SD, n=3) (**d**) Semi-quantitative analyses of the blots in Fig. 4f. (means ± SD, n=3). (**e**) IPs by Flag beads from 293FT cells transfected with the indicated LKB1-Flag for 48 h were subjected to IB. (**f**) Semi-quantitative analyses of the blots in Fig. 4g. (means ± SD, n=3). (**g**) Semi-quantitative analyses of the blots in Fig. 4i. (means ± SD, n=3). (**h**) Semi-quantitative analyses of the blots in Fig. 4j. (means ± SD, n=3). (**i**) Immunofluorescent assay to investigate the subcellular localization of LKB1-WT/3KR. A549 cells were transfected with LKB1-WT/3KR-Flag for 48 h, followed by immunofluorescent assay. (means ± SEM, n=50). n.s: *P* > 0.05; **P* < 0.05; ***P* < 0.01; ****P* < 0.001.


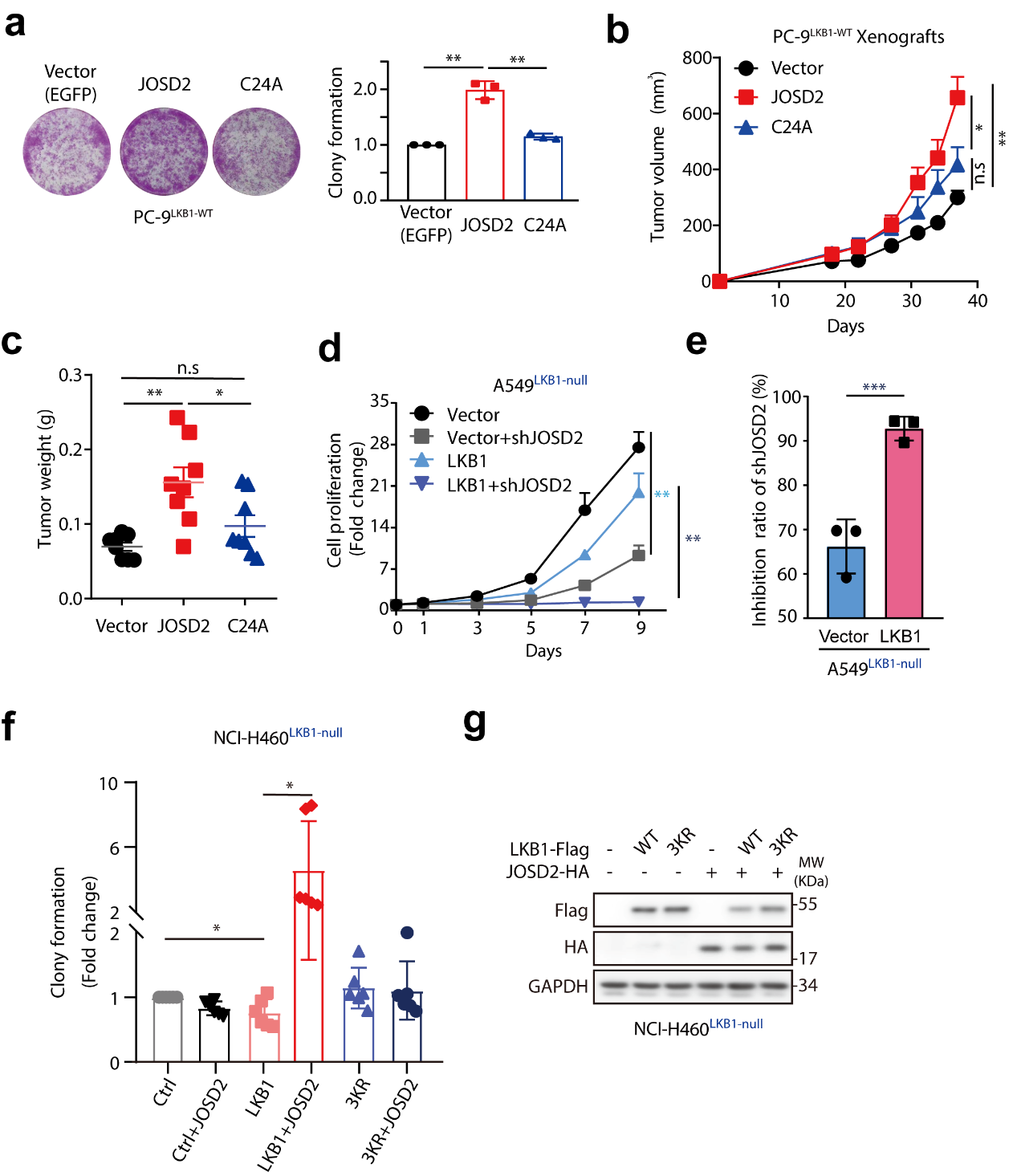


**Figure. S7. LKB1 was critical for JOSD2 in regulating cell proliferation**

(**a**) Clony formation assay in PC-9 cells infected with lentiviruses packaged Vector (EGFP), JOSD2-WT or C24A. PC-9 cells were infected with indicated lentiviruses for 96 h, then these cells were seeded 2000 cells per well in 6-well plates, followed by SRB staining (means ± SD, n=3). (**b**) Tumor volume of Vector/JOSD2/C24A-expressing tumors of PC-9 xenografts. (**c**) Tumor weight of each mice in indicated groups (at the endpoint) of PC-9 xenografts (means ± SEM, n=10). (**d**) Cell proliferation assay in LKB1-null/WT A549 cells transfected with scramble control or JOSD2 shRNA. A549 cells harboring LKB1-null/WT were infected with indicated lentiviruses for 96 h, then these cells were seeded 1000 cells per well in 96-well plates, followed by SRB staining (means ± SD, n=3). (**e**) The inhibition ratio of JOSD2 knockdown on cell proliferation in A549 cells harboring LKB1-null/WT (means ± SD, n=3). (**f**) Statistical analysis of colony formation assay in Fig. 5g (means ± SD, n=6). (**g**) IB analysis showing the overexpression efficiency of LKB1-WT/3KR and JOSD2 (means ± SD, n=3). n.s: *P* > 0.05; **P* < 0.05; ***P* < 0.01; ****P* < 0.001.


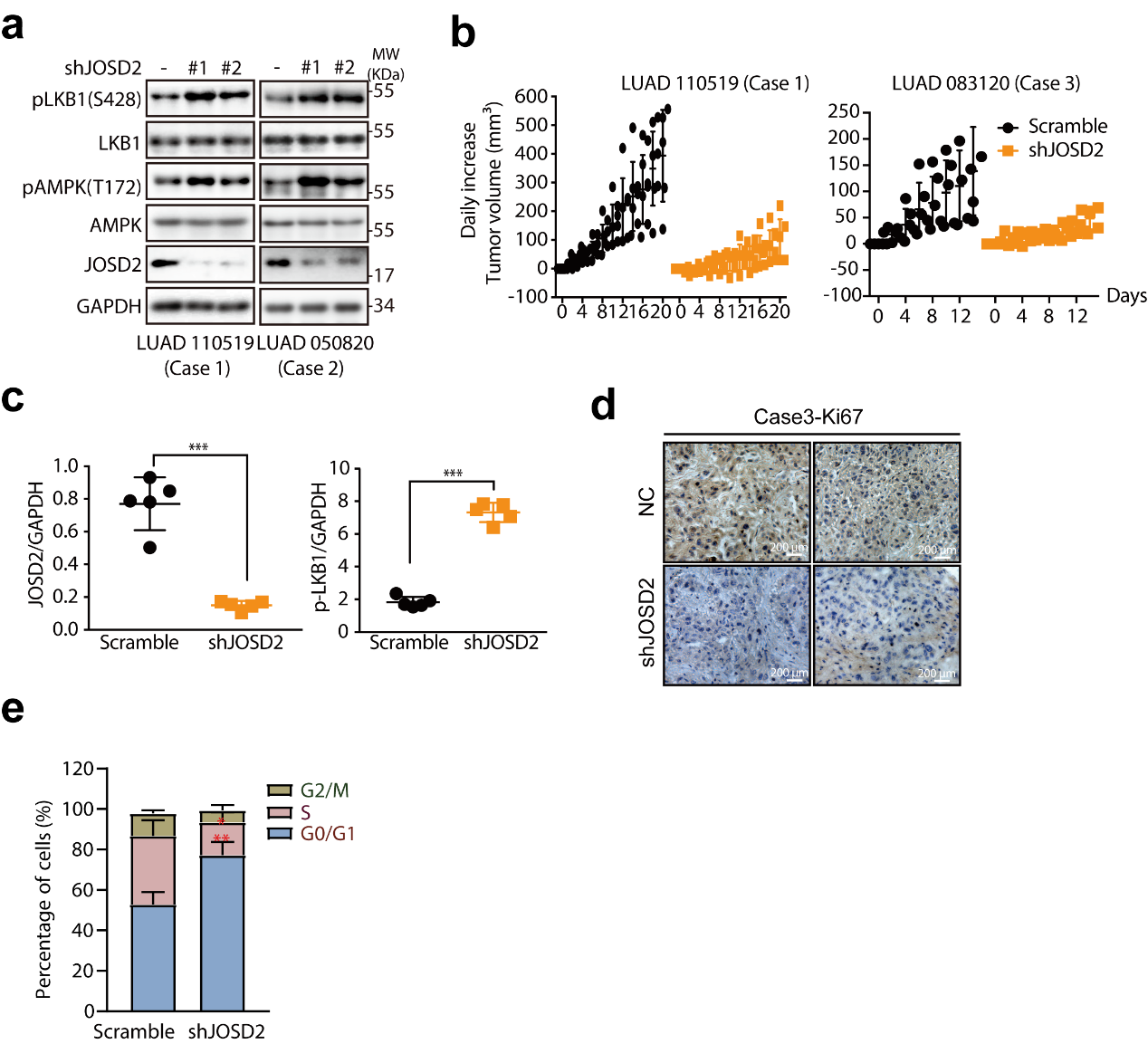


**Figure. S8. Analysis of related protein levels in PDCs and PDXs**

(**a**) Cell lysates of Case 1 and Case 2 PDCs transfected with scramble control or JOSD2 shRNA for 96 h were subjected to IB. (**b**) Daily increase in tumor volume of each mice in indicated groups recorded by days. (**c**) Quantification of p-LKB1 and JOSD2 protein levels in Fig. 6g. (means ± SD, n=5). (**d**) Representative images of Ki67 IHC staining in NC (negative control) group and JOSD2 knockdown group of PDX (case 3) tumors. (**e**) NCI-H1299 transfected with scramble control or JOSD2 shRNA for 96 h were subjected to flow cytometry. **P* < 0.05; ***P* < 0.01; ****P* < 0.001.


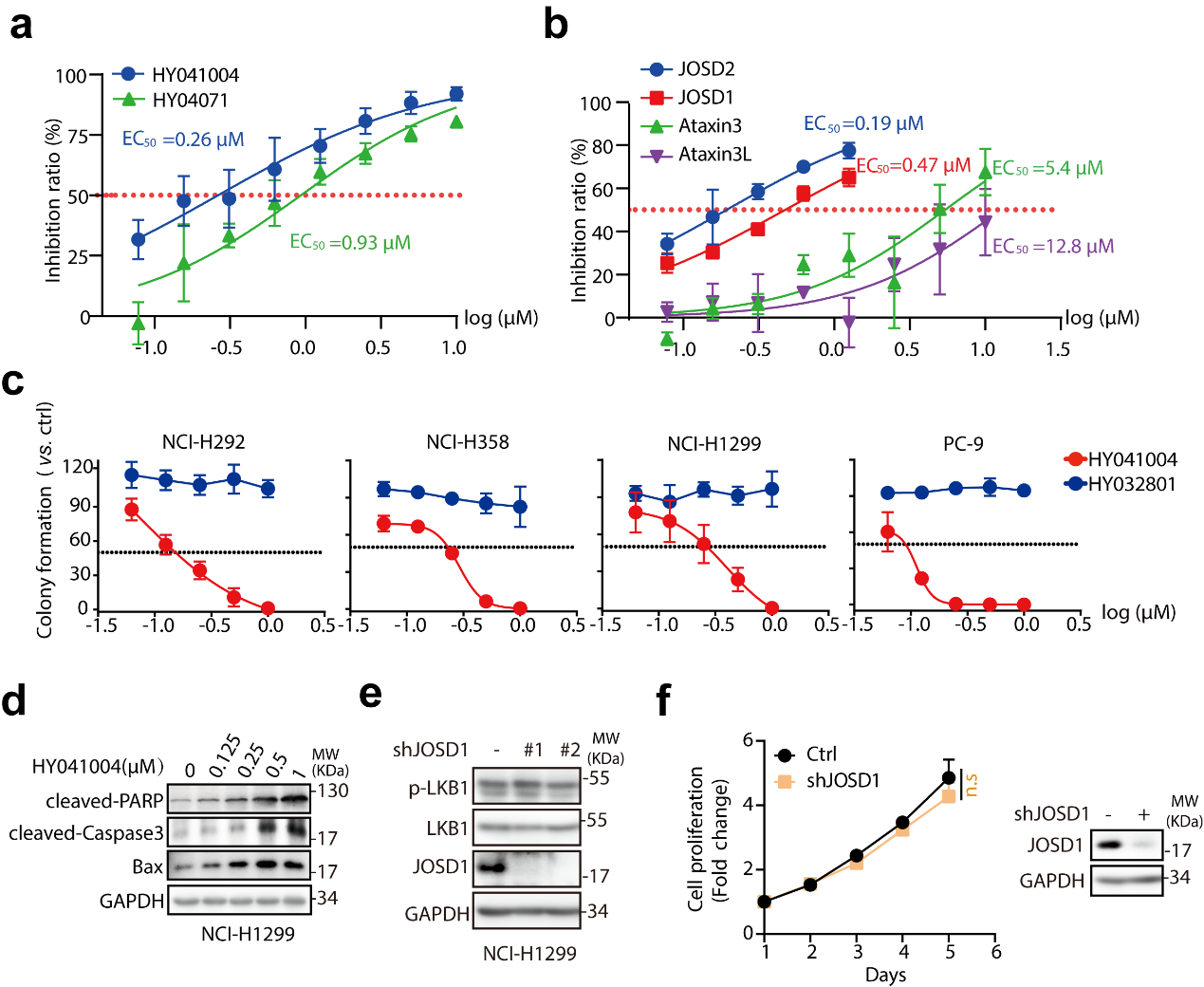


**Figure. S9. HY041004 showed a selectivity among MJDs with the most potent inhibitory effects against JOSD2**

(**a**) The IC_50_ values of HY041004 and HY04071 on JOSD2 catalytic activities. The indicated concentration HY041004 and HY04071 were incubated with GST-JOSD2 protein at 37°C for 30 minutes and then subjected to *in vitro* ubiquitin-AMC assay. (means ± SD, n=3). (**b**) the IC_50_ values of HY041004 on MJDs family members including JOSD2, JOSD1, Ataxin-3 and Ataxin-3L. The indicated concentration HY041004 and HY04071 were incubated with GST-JOSD2/JOSD1/Ataxin-3/ Ataxin-3L protein at 37°C for 30 minutes and then subjected to *in vitro* ubiquitin-AMC assay. (means ± SD, n=3). (**c**) Statistical analysis of colony formation assay in Fig. 8b. (means ± SD, n=3). (**d**) Cell lysates of NCI-H1299 treated with a series of concentrations HY041004 for 72 hours were subjected to IB. (**e**) Cell lysates of NCI-H1299 transfected with scramble control or JOSD1 shRNA for 96 h were subjected to IB. (**f**) Cell proliferation assay in NCI-H1299 cells transfected with scramble control or JOSD1 shRNA (left). NCI-H1299 cells were infected with indicated lentiviruses for 96 h and seeded 1000 cells per well in 96-well plates, followed by SRB staining. (means ± SD, n=3). IB analysis showing the knockdown efficiency of JOSD1 (right). n.s: *P* > 0.05.


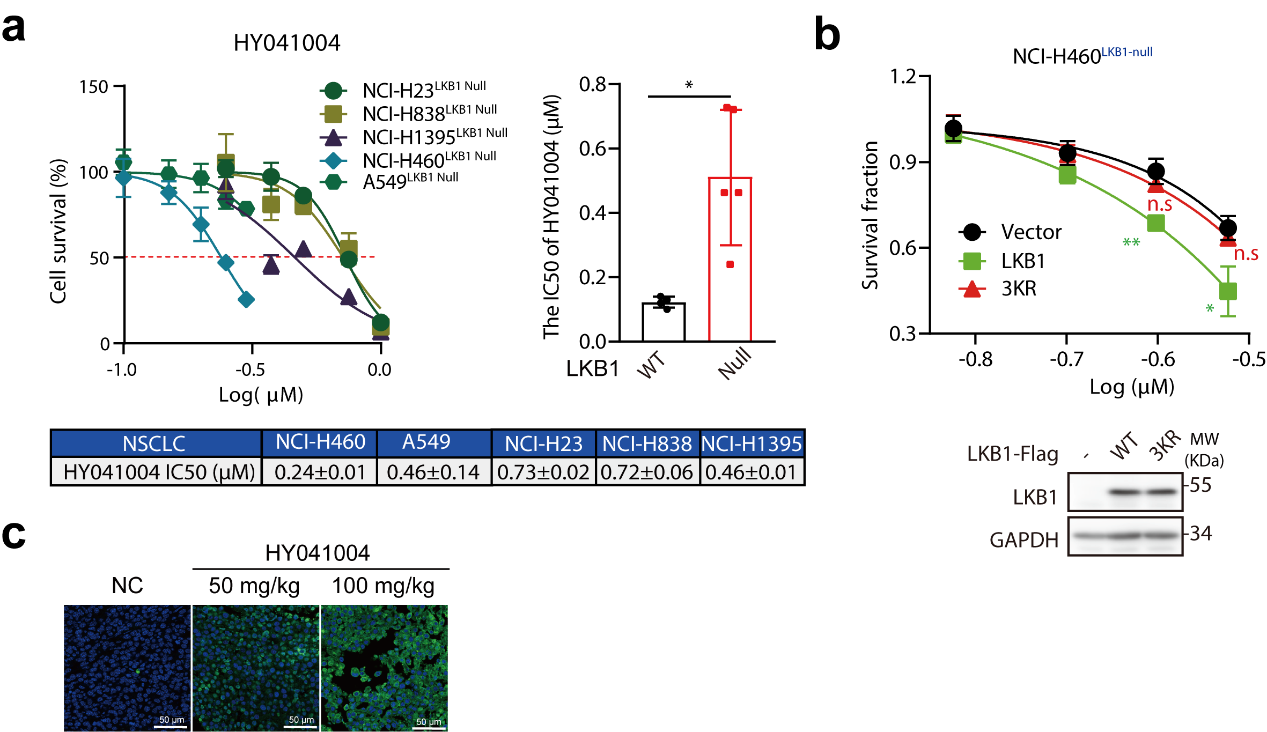


**Figure. S10. HY041004 exerted the anti-cancer activity through its regulation on LKB1**

(**a**) Proliferation assay in LKB1-null cells (means ± SD, n=3). The indicated cell lines were treated with a series of concentrations HY041004 for 72 hours, followed by cell proliferation assay. Red dashed line indicates 50% of cell survival. (**b**) Proliferation assay in A549 harboring LKB1-null/WT/3KR (Top) and immunoblotting analysis showing the overexpression efficiency of LKB1-WT/3KR (Bottom). NCI-H460 cells harboring LKB1-null/WT/3KR were treated with a series of concentrations HY041004 for 72 hours, followed by cell proliferation assay. (means ± SD, n=3). (**c**) Representative images of *in situ* TUNEL assay of cancer cells inside tumor mass with or without JOSD2 inhibitor treatment. (means ± SD, n=3). n.s: *P* > 0.05; **P* < 0.05; ***P* < 0.01.


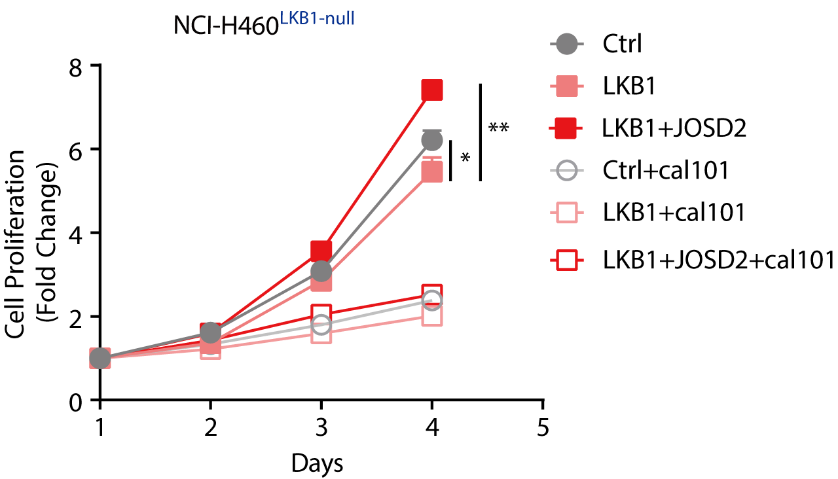


**Figure. S11. CAL-101 could almost completely abolish the accelerated cell proliferation triggered by JOSD2+LKB1**

Cell proliferation assays of NCI-H460 cells harboring LKB1-null/WT transfected with JOSD2 with treatment with DMSO or 2.5 μM cal-101 (means ± SD, n=3). **P* < 0.05; ***P* < 0.01.

**Table S1. Patients’ information of 80 LUADs, related to Fig. 1d**

| **Serial Number** | **Pathology  Number** | **Sex** | **Age** | **Grading** | **T** | **N** | **M** | **T1bN0M1** | **TNM Stage** | **Pathological Types of Tumor** |
| --- | --- | --- | --- | --- | --- | --- | --- | --- | --- | --- |
| A-1 | ZHFX001 | Female | 72 |  | T2 | N0 | M0 | T2N0M0 | IB | Peripheral adenocarcinoma with bronchioloalveolar carcinoma |
| A-2 | ZHFX002 | Male | 74 | 2 |  |  |  | T2N2M0 | IIIA | Adenocarcinoma |
| A-3 | ZHFX003 | Female | 56 | 2 | T2 | N2 | M0 | T2N2M0 | III | Adenocarcinoma |
| A-4 | ZHFX004 | Male | 63 | 21 | T2 | N2 | M0 | T2N2M0 | III | Adenocarcinoma |
| A-5 | ZHFX005 | Female | 61 | 21 | T2 | N2 | M0 | T2N2M0 | III | Adenocarcinoma |
| A-6 | ZHFX006 | Female | 52 |  | T1c | N2 | M0 | T1cN2M0 | IIIA | Adenocarcinoma with mucinous adenocarcinoma and bronchioloalveolar carcinoma |
| A-7 | ZHFX007 | Female | 71 | 2 | T2 | N2 | M0 | T2N2M0 | III | Adenocarcinoma |
| A-8 | ZHFX008 | Male | 72 | 21 | T3 | N2 | M1 | T3N2M1 | IV | Adenocarcinoma |
| A-9 | ZHFX009 | Male | 75 | 21 | T2 | N0 | M0 | T2N0M0 | IB | Adenocarcinoma |
| A-10 | ZHFX010 | Male | 59 | 2 | T3 | N0 | M0 | T3N0M0 | IIB | Bronchioloalveolar carcinoma |
| A-11 | ZHFX011 | Male | 60 | 21 | T1 | N1 | M0 | T1N1M0 | IIB | Adenocarcinoma |
| A-12 | ZHFX012 | Male | 66 | 21 | T1 | N2 | M0 | T1N2M0 | IIIA | Adenocarcinoma |
| A-13 | ZHFX013 | Female | 60 | 21 | T3 | N2 | M0 | T3N2M0 | IIIB | Adenocarcinoma |
| A-14 | ZHFX014 | Male | 58 |  | T3 | N0 | M0 | T3N0M0 | IIB | Adenocarcinoma |
| A-15 | ZHFX015 | Female | 59 | 21 | T1 | N0 | M0 | T1N0M0 | IA | Adenocarcinoma |
| A-16 | ZHFX016 | Female | 56 |  | T2 | N0 | M0 | T2N0M0 | IB | Adenocarcinoma |
| B-1 | ZHFX017 | Male | 64 | 1 | T3 | N0 | M1 | T3N0M1 | IV | Adenocarcinoma |
| B-2 | ZHFX018 | Male | 47 | 2 | T2b | N0 | M0 | T2bN0M0 | IIA | Adenocarcinoma |
| B-3 | ZHFX019 | Female | 64 | 2 | T1c | N1 | M0 | T1cN1M0 | IIB | Adenocarcinoma |
| B-4 | ZHFX020 | Male | 73 | 2 | T2a | N0 | M0 | T2aN0M0 | IB | Adenocarcinoma |
| B-5 | ZHFX021 | Male | 70 |  | T3 | N0 | M0 | T3N0M0 | IIB | Squamous cell carcinoma |
| B-6 | ZHFX022 | Male | 62 |  | T4 | N2 | M0 | T4N2M0 | IIIB | Squamous cell carcinoma |
| B-7 | ZHFX023 | Female | 62 | 21 | T1c | N2 | M0 | T1cN2M0 | IIIA | Adenocarcinoma |
| B-8 | ZHFX024 | Male | 48 | low | T3 | N0 | M0 | T3N0M1 | IV | Adenocarcinoma |
| B-9 | ZHFX025 | Male | 60 |  | T2a | N1 | M0 | T2aN1M0 | IIA | Adenocarcinoma |
| B-10 | ZHFX026 | Female | 54 |  | T1c | N0 | M0 | T1cN0M0 | IA | Adenocarcinoma |
| B-11 | ZHFX027 | Male | 56 | 21 | T2 | N2 | M0 | T2N2M0 | III | Adenocarcinoma |
| B-12 | ZHFX028 | Female | 58 |  | T2a | N2 | M0 | T2aN2M0 | IIIA | Adenocarcinoma |
| B-13 | ZHFX029 | Female | 48 |  | T2b | N1 | M1 | T2bN1M1 | IV | Adenocarcinoma |
| B-14 | ZHFX030 | Female | 52 |  | T2a | N2 | M1 | T2aN2M1 | IV | Adenocarcinoma |
| B-15 | ZHFX031 | Male | 58 | 2 | T2a | N0 | M1 | T2aN0M1 | IV | Adenocarcinoma |
| B-16 | ZHFX032 | Female | 38 | 21 | T1 | N0 | M1 | T1N0M1 | IV | Adenocarcinoma |
| C-1 | ZHFX033 | Male | 63 | 2 | T2a | N1 | M1 | T2aN1M1 | IV | Adenocarcinoma |
| C-2 | ZHFX034 | Female | 55 | 23 | T1c | N0 | M0 | T1cN0M0 | IA | Adenocarcinoma |
| C-3 | ZHFX035 | Female | 66 | 2 | T2a | N1 | M0 | T2aN1M0 | IIA | Squamous adenocarcinoma |
| C-4 | ZHFX036 | Female | 37 | 21 | T3 | N0 | M0 | T3N0M0 | IIB | Adenocarcinoma |
| C-5 | ZHFX037 | Female | 76 | 21 | T2b | N0 | M0 | T2bN0M0 | IIB | Adenocarcinoma |
| C-6 | ZHFX038 | Male | 40 | 2 | T2 | N2 | M1 | T2N2M1 | IV | Adenocarcinoma |
| C-7 | ZHFX039 | Female | 46 | 2 | T2b | N2 | M0 | T2bN2M0 | IIIA | Adenocarcinoma |
| C-8 | ZHFX040 | Male | 66 |  | T2b | N0 | M0 | T2bN0M0 | IIA | Adenocarcinoma |
| C-9 | ZHFX041 | Female | 64 | 1 | T3 | N1 | M0 | T3N1M0 | IIIA | Adenocarcinoma |
| C-10 | ZHFX042 | Male | 57 | 2 | T2 | N2 | M0 | T2N2M0 | IIIA | Squamous adenocarcinoma |
| C-11 | ZHFX043 | Male | 49 | 23 | T2 | N2 | M0 | T2N2M1 | IV | Adenocarcinoma |
| C-12 | ZHFX044 | Female | 37 | 1 | T3 | N1 | M0 | T3N1M1 | IV | Adenocarcinoma |
| C-13 | ZHFX045 | Female | 57 | 21 | T1 | N1 | M0 | T1N1M0 | IIB | Adenocarcinoma |
| C-14 | ZHFX046 | Female | 47 |  | T2 | N1 | M0 | T2N1M0 | II | Adenocarcinoma |
| C-15 | ZHFX047 | Female | 60 | 2 | T2a | N0 | M0 | T2aN0M0 | IB | Adenocarcinoma |
| C-16 | ZHFX048 | Male | 74 |  | T3 | N0 | M1 | T3N0M1 | IV | Large cell carcinoma |
| D-1 | ZHFX049 | Male | 61 | 2 | T2b | N1 | M0 | T2bN1M0 | IIB | Adenocarcinoma |
| D-2 | ZHFX050 | Male | 54 |  | T1c | N0 | M1 | T1cN0M1 | IV | Adenocarcinoma |
| D-3 | ZHFX051 | Male | 58 |  | T2a | N0 | M0 | T2aN0M0 | IB | Adenocarcinoma |
| D-4 | ZHFX052 | Female | 65 | 21 | T1 | N2 | M0 | T1N2M0 | IIIA | Large cell carcinoma |
| D-5 | ZHFX053 | Female | 67 |  | T2a | N0 | M0 | T2aN0M0 | IB | Adenocarcinoma |
| D-6 | ZHFX054 | Female | 57 | 23 | T1C | N0 | M0 | T1CN0M0 | IA | Adenocarcinoma |
| D-7 | ZHFX055 | Male | 80 | 2 | T2b | N0 | M0 | T2bN0M0 | IIIA | Adenocarcinoma |
| D-8 | ZHFX056 | Female | 62 | 2 | T1 | N2 | M0 | T1N2M0 | IIIA | Adenocarcinoma |
| D-9 | ZHFX057 | Female | 60 | 2 | T2 | N0 | M0 | T2N0M0 | IB | Adenocarcinoma |
| D-10 | ZHFX058 | Female | 53 |  | TX | N2 | M1 | TXN2M1 | IV | Adenocarcinoma |
| D-11 | ZHFX059 | Female | 58 | 2 | T1 | N2 | M0 | T1N2M0 | IIIA | Adenocarcinoma |
| D-12 | ZHFX060 | Female | 61 | 1 | T1 | N0 | M0 | T1N0M0 | IA | Adenocarcinoma |
| D-13 | ZHFX061 | Male | 58 | 23 | T2a | N0 | M0 | T2aN0M0 | IB | Adenocarcinoma |
| D-14 | ZHFX062 | Male | 57 | 1 | T3 | N2 | M0 | T3N2M0 | IIIB | Adenocarcinoma |
| D-15 | ZHFX063 | Male | 58 | 21 | T1 | N1 | M0 | T1N1M0 | IIB | Adenocarcinoma |
| D-16 | ZHFX064 | Male | 56 |  | T4 | N0 | M0 | T4N0M0 | IIIA | Adenocarcinoma |
| E-1 | ZHFX065 | Female | 61 | 3 | T1 | N0 | M0 | T1N0M0 | IA | Adenocarcinoma |
| E-2 | ZHFX066 | Male | 58 |  | T1 | N0 | M0 | T1N0M0 | IA | Peripheral bronchioloalveolar carcinoma |
| E-3 | ZHFX067 | Female | 61 | 21 | T2a | N1 | M0 | T2aN1M0 | IIA | Adenocarcinoma |
| E-4 | ZHFX068 | Female | 67 | 23 | T2a | N0 | M0 | T2aN0M0 | IB | Adenocarcinoma |
| E-5 | ZHFX069 | Female | 63 | 2 | T1 | N1 | M0 | T1N1M0 | IIB | Adenocarcinoma |
| E-6 | ZHFX070 | Male | 47 | 21 | T3 | N0 | M0 | T3N0M0 | IIB | Adenocarcinoma |
| E-7 | ZHFX071 | Male | 61 |  | TX | N0 | M1 | TXN0M1 | IV | Adenocarcinoma |
| E-8 | ZHFX072 | Male | 45 |  | T4 | N0 | M0 | T4N0M0 | IIIA | Adenocarcinoma |
| E-9 | ZHFX073 | Male | 81 | 21 | T2a | N0 | M0 | T2aN0M0 | IB | Adenocarcinoma |
| E-10 | ZHFX074 | Female | 49 | 2 | T3 | N1 | M0 | T3N1M0 | IIIA | Adenocarcinoma |
| E-11 | ZHFX075 | Male | 60 | 2 | T2 | N0 | M0 | T2N0M0 | IIB | Adenocarcinoma |
| E-12 | ZHFX076 | Male | 57 | 2 | T1 | N2 | M0 | T1N2M0 | IIIA | Adenocarcinoma |
| E-13 | ZHFX077 | Male | 47 | 2 | T2 | N0 | M0 | T2N0M0 | IB | Adenocarcinoma |
| E-14 | ZHFX078 | Male | 53 | 1 | T4 | N0 | M0 | T4N0M0 | IIIA | Peripheral poorly differentiated carcinoma |
| E-15 | ZHFX079 | Male | 65 |  |  |  | M1 |  | IV | Adenocarcinoma |
| E-16 | ZHFX080 | Female | 65 | 21 | T2 | N1 | M1 | T2N1M1 | IV | Adenocarcinoma |

Table S2. The identified proteins binding to JOSD2 by AP-MS

| **Number** | **Gene names** | **MW [kDa]** | **Protein score** | **Sequence coverage (%)** | **# Unique Peptides** | **# Peptides** |
| --- | --- | --- | --- | --- | --- | --- |
| 1 | JOSD2 | 20.7 | 8018 | 71 | 12 | 12 |
| 2 | SEC16A | 251.7 | 1546 | 35 | 49 | 49 |
| 3 | AHCYL1 | 58.9 | 556 | 36 | 7 | 14 |
| **4** | **STK11** | **48.6** | **525** | **25** | **9** | **9** |
| 5 | MDC1 | 226.5 | 487 | 13 | 15 | 15 |
| 6 | STRADA | 48.3 | 483 | 38 | 13 | 13 |
| 7 | CAB39 | 39.8 | 469 | 33 | 9 | 11 |
| 8 | AHCYL2 | 66.7 | 361 | 16 | 3 | 10 |
| 9 | NUFIP2 | 76.1 | 315 | 15 | 7 | 7 |
| 10 | RCN2 | 36.9 | 310 | 28 | 6 | 6 |
| 11 | CSNK1D | 47.3 | 285 | 18 | 2 | 6 |
| 12 | CHTOP | 26.4 | 217 | 21 | 5 | 5 |
| 13 | STRADB | 47 | 199 | 29 | 7 | 7 |
| 14 | KHDRBS3 | 38.8 | 197 | 13 | 3 | 5 |
| 15 | CP | 122.1 | 196 | 5 | 5 | 5 |
| 16 | PPP2R2D | 52 | 192 | 22 | 2 | 7 |
| 17 | CALU | 37.1 | 188 | 27 | 6 | 6 |
| 18 | HNRNPH3 | 36.9 | 183 | 25 | 6 | 7 |
| 19 | MACROH2A1 | 39.2 | 181 | 18 | 5 | 6 |
| 20 | DHRS7B | 35.1 | 159 | 12 | 3 | 3 |
| 21 | CNN3 | 36.4 | 144 | 23 | 5 | 5 |
| 22 | ATAD3B | 72.5 | 142 | 8 | 5 | 5 |
| 23 | RHOA | 21.8 | 133 | 21 | 4 | 4 |
| 24 | YLPM1 | 241.5 | 127 | 3 | 5 | 5 |
| 25 | FLG2 | 247.9 | 124 | 2 | 3 | 3 |
| 26 | ANKEF1 | 86.6 | 122 | 9 | 6 | 6 |
| 27 | AKAP8 | 76.1 | 115 | 13 | 5 | 5 |
| 28 | STOML2 | 38.5 | 115 | 9 | 2 | 2 |
| 29 | GTF2H2 | 44.4 | 106 | 19 | 5 | 5 |
| 30 | DNAJB9 | 25.5 | 97 | 15 | 2 | 2 |
| 31 | IQSEC2 | 162.7 | 84 | 3 | 3 | 3 |
| 32 | RSF1 | 163.7 | 78 | 3 | 3 | 3 |
| 33 | G3BP2 | 54.1 | 78 | 9 | 3 | 4 |
| 34 | TOMM40 | 37.9 | 76 | 12 | 2 | 2 |
| 35 | CLUH | 146.6 | 72 | 1 | 2 | 2 |
| 36 | GANAB | 106.8 | 69 | 6 | 3 | 3 |
| 37 | PSMD2 | 100.1 | 66 | 3 | 2 | 2 |
| 38 | CKB | 42.6 | 65 | 11 | 3 | 3 |
| 39 | AHCY | 47.7 | 65 | 6 | 3 | 3 |
| 40 | RPA2 | 29.2 | 65 | 17 | 2 | 2 |
| 41 | CDK4 | 33.7 | 64 | 9 | 2 | 2 |
| 42 | MRPL2 | 33.3 | 64 | 14 | 3 | 3 |
| 43 | IGHA1 | 37.6 | 64 | 10 | 3 | 3 |
| 44 | ZNF467 | 65.1 | 59 | 8 | 2 | 2 |
| 45 | PDK3 | 46.9 | 59 | 10 | 4 | 4 |
| 46 | GIPC1 | 36 | 56 | 9 | 2 | 2 |
| 47 | SAR1A | 22.4 | 55 | 22 | 3 | 3 |
| 48 | RRAS2 | 23.4 | 54 | 10 | 2 | 2 |
| 49 | EMILIN2 | 115.6 | 53 | 2 | 2 | 2 |
| 50 | PHIP | 206.6 | 52 | 1 | 2 | 2 |
| 51 | IQGAP2 | 180.5 | 51 | 1 | 2 | 2 |
| 52 | EIF3B | 92.4 | 50 | 3 | 2 | 2 |
| 53 | PPHLN1 | 52.7 | 50 | 3 | 2 | 2 |
| 54 | PSMC6 | 44.1 | 50 | 12 | 2 | 2 |
| 55 | PSMC2 | 48.6 | 49 | 4 | 2 | 2 |
| 56 | SNX29 | 91.2 | 49 | 3 | 2 | 2 |
| 57 | RANGAP1 | 63.5 | 49 | 6 | 2 | 2 |
| 58 | WDR43 | 74.8 | 48 | 3 | 2 | 2 |
| 59 | CGN | 137 | 48 | 1 | 2 | 2 |
| 60 | ZNF770 | 80 | 48 | 2 | 2 | 2 |
| 61 | CCT2 | 57.5 | 48 | 8 | 2 | 2 |
| 62 | RAB29 | 23.1 | 46 | 10 | 2 | 2 |
| 63 | MRPS18C | 15.8 | 44 | 13 | 2 | 2 |
| 64 | GH2 | 25 | 44 | 8 | 2 | 2 |
| 65 | DVL1 | 75.1 | 41 | 6 | 2 | 2 |
| 66 | DAPK3 | 52.5 | 38 | 7 | 2 | 2 |
| 67 | STK35 | 58 | 38 | 3 | 2 | 2 |
| 68 | ZNF775 | 59.7 | 38 | 9 | 2 | 2 |
| 67 | TRIP6 | 50.3 | 36 | 7 | 2 | 2 |
| 70 | ACTR1A | 42.6 | 36 | 7 | 2 | 2 |
| 71 | PSMC3 | 49.2 | 35 | 7 | 2 | 2 |
| 72 | ACOT9 | 49.9 | 34 | 5 | 2 | 2 |
| 73 | NDUFS8 | 23.7 | 33 | 10 | 2 | 2 |

MW [kDa]: Protein molecular weight; Protein score：Score for protein matching degree (the higher score, the better matching), the protein score is derived from the ions scores and represented the confidence of identification. In Mascot, the ions score for an MS/MS match is based on the calculated probability, *P*, that the observed match between the experimental data and the database sequence is a random event. The reported score is -10Log(*P*). The protein score is normally a sum of all possible matches；Sequence coverage (%)：Percent of identified peptide sequence covering in protein sequence；Unique Peptides：Number of unique peptides which spectrums hit；Peptides：Number of peptides which spectrums hit. The detailed interpretation and instructions of MS/MS results are available on the official website of Matrix Science (http://www.matrixscience.com/help/interpretation_help.html).

Table S3. The identified proteins binding to JOSD2 by LC-MS.

| **Number** | **Gene names** | **MW [kDa]** | **Protein score** | **Sequence coverage (%)** | **# Unique Peptides** | **# Peptides** |
| --- | --- | --- | --- | --- | --- | --- |
| 1 | MYH9 | 226.4 | 10108 | 50 | 82 | 96 |
| 2 | MYH10 | 228.9 | 7306 | 43 | 65 | 77 |
| 3 | SPTAN1 | 284.4 | 3413 | 36 | 63 | 63 |
| 4 | MYH14 | 227.7 | 2875 | 32 | 40 | 48 |
| 5 | MYO6 | 149.6 | 2475 | 42 | 40 | 40 |
| 6 | DBN1 | 71.4 | 2413 | 50 | 24 | 24 |
| 7 | SPTBN1 | 274.4 | 1674 | 22 | 37 | 37 |
| 8 | PARP1 | 113 | 1671 | 36 | 29 | 29 |
| 9 | TUBB | 49.6 | 1359 | 60 | 6 | 18 |
| 10 | LIMA1 | 85.2 | 1242 | 33 | 16 | 16 |
| 11 | MYO1B | 131.9 | 1164 | 26 | 21 | 21 |
| 12 | TUBB4B | 49.8 | 1144 | 47 | 3 | 15 |
| 13 | MYO1C | 121.6 | 1027 | 28 | 24 | 24 |
| 14 | MYL12B | 19.8 | 970 | 56 | 8 | 8 |
| 15 | H2BC18 | 13.9 | 962 | 41 | 5 | 6 |
| 16 | MYO1D | 116.1 | 907 | 34 | 28 | 28 |
| 17 | MYL6 | 16.9 | 905 | 76 | 7 | 9 |
| 18 | CAPZA1 | 32.9 | 871 | 62 | 9 | 11 |
| 19 | MPRIP | 116.5 | 814 | 28 | 20 | 20 |
| 20 | TUBA1B | 50.1 | 793 | 39 | 2 | 11 |
| 21 | TMOD3 | 39.6 | 790 | 45 | 12 | 14 |
| 22 | TMOD1 | 40.5 | 763 | 52 | 14 | 15 |
| 23 | CAPZB | 31.3 | 727 | 55 | 12 | 12 |
| 24 | MYO18A | 233 | 688 | 11 | 17 | 17 |
| 25 | HNRNPH2 | 49.2 | 677 | 28 | 4 | 9 |
| 26 | FLII | 144.7 | 675 | 16 | 16 | 16 |
| 27 | TPM4 | 28.5 | 657 | 42 | 5 | 12 |
| 28 | JOSD2 | 20.7 | 624 | 41 | 7 | 7 |
| 29 | SPECC1L | 124.5 | 567 | 20 | 15 | 15 |
| 30 | XRCC6 | 69.8 | 566 | 27 | 12 | 12 |
| 31 | TPM3 | 32.9 | 522 | 34 | 5 | 11 |
| 32 | BANF1 | 10.1 | 507 | 56 | 5 | 5 |
| 33 | PPP1R12A | 115.2 | 492 | 12 | 9 | 9 |
| 34 | CAPZA2 | 32.9 | 491 | 42 | 7 | 9 |
| 35 | PPP1R10 | 99 | 490 | 22 | 13 | 13 |
| 36 | ACTR3 | 47.3 | 488 | 42 | 11 | 11 |
| 37 | EFHD2 | 26.7 | 487 | 40 | 6 | 8 |
| 38 | VIM | 53.6 | 486 | 26 | 10 | 10 |
| 39 | CALM2 | 16.8 | 483 | 50 | 6 | 6 |
| 40 | PPP1CC | 37 | 463 | 41 | 2 | 11 |
| 41 | ACTR2 | 44.7 | 454 | 21 | 6 | 6 |
| 42 | RAI14 | 110 | 448 | 19 | 14 | 14 |
| 43 | TPM2 | 32.8 | 439 | 27 | 3 | 10 |
| 44 | LRRFIP2 | 82.1 | 415 | 20 | 10 | 10 |
| 45 | MYL6B | 22.8 | 410 | 46 | 7 | 9 |
| 46 | TPM1 | 32.7 | 403 | 28 | 4 | 10 |
| 47 | PPP1R9B | 89.3 | 395 | 15 | 8 | 9 |
| 48 | LRCH3 | 86 | 373 | 30 | 13 | 13 |
| 49 | CAB39 | 39.8 | 342 | 17 | 5 | 5 |
| 50 | RPS23 | 15.8 | 333 | 43 | 5 | 5 |
| 51 | AMOT | 118 | 331 | 13 | 10 | 10 |
| 52 | TJP1 | 195.3 | 320 | 5 | 6 | 6 |
| 53 | STRADA | 48.3 | 316 | 19 | 5 | 5 |
| 54 | ARPC4 | 19.7 | 308 | 35 | 5 | 5 |
| 55 | TMPO | 50.6 | 287 | 18 | 5 | 5 |
| 56 | ARPC3 | 20.5 | 276 | 35 | 4 | 4 |
| 57 | ARPC1A | 41.5 | 261 | 25 | 5 | 6 |
| 58 | INCENP | 105.4 | 250 | 10 | 6 | 6 |
| 59 | RCN2 | 36.9 | 247 | 18 | 4 | 4 |
| 60 | EEF1A1 | 50.1 | 241 | 23 | 8 | 8 |
| 61 | EFHD1 | 26.9 | 236 | 21 | 2 | 4 |
| 62 | CGN | 136.3 | 232 | 6 | 5 | 5 |
| 63 | CBX3 | 20.8 | 218 | 45 | 4 | 5 |
| 64 | CHERP | 103.6 | 217 | 6 | 3 | 3 |
| 65 | AIF1L | 17.1 | 217 | 27 | 3 | 3 |
| **66** | **STK11** | **48.6** | **215** | **14** | **4** | **4** |
| 67 | ABLIM1 | 87.6 | 208 | 7 | 4 | 4 |
| 68 | XRCC5 | 82.7 | 202 | 8 | 4 | 4 |
| 69 | ACTN4 | 104.8 | 199 | 6 | 5 | 5 |
| 70 | DEK | 42.6 | 194 | 16 | 4 | 4 |
| 71 | CBX5 | 22.2 | 192 | 33 | 5 | 5 |
| 72 | PCNA | 28.8 | 189 | 23 | 4 | 4 |
| 73 | RPL37A | 10.3 | 180 | 46 | 4 | 4 |
| 74 | ARPC2 | 34.3 | 179 | 31 | 7 | 7 |
| 75 | RPL10 | 24.6 | 170 | 35 | 6 | 6 |
| 76 | PCMT1 | 24.6 | 170 | 22 | 3 | 3 |
| 77 | MAZ | 48.6 | 165 | 13 | 4 | 4 |
| 78 | MACROH2A1 | 39.6 | 161 | 26 | 6 | 6 |
| 79 | TMOD2 | 39.6 | 161 | 19 | 4 | 5 |
| 80 | GSN | 85.6 | 158 | 5 | 3 | 3 |
| 81 | RPS9 | 22.6 | 152 | 22 | 5 | 5 |
| 82 | RPS3 | 26.7 | 152 | 25 | 5 | 5 |
| 83 | ARPC5 | 16.3 | 149 | 33 | 4 | 4 |
| 84 | HSPA9 | 73.6 | 149 | 6 | 3 | 3 |
| 85 | MDC1 | 226.5 | 147 | 2 | 3 | 3 |
| 86 | IRS4 | 133.7 | 142 | 3 | 2 | 2 |
| 87 | RPL13 | 24.2 | 142 | 15 | 3 | 3 |
| 88 | H2AC21 | 14 | 137 | 30 | 2 | 3 |
| 89 | VRK1 | 45.4 | 137 | 15 | 5 | 5 |
| 90 | SSBP1 | 17.2 | 137 | 22 | 2 | 2 |
| 91 | SH3BGRL2 | 12.3 | 137 | 25 | 3 | 3 |
| 92 | PARP2 | 66.2 | 131 | 6 | 2 | 2 |
| 93 | DOCK7 | 242.4 | 130 | 2 | 3 | 3 |
| 94 | ARF3 | 20.6 | 125 | 22 | 2 | 3 |
| 95 | RCC1 | 44.9 | 125 | 12 | 3 | 3 |
| 96 | ENO1 | 47.1 | 122 | 7 | 2 | 2 |
| 97 | MYO1E | 127 | 122 | 3 | 3 | 3 |
| 98 | RPL9 | 21.9 | 121 | 24 | 2 | 2 |
| 99 | RPS6 | 28.7 | 119 | 11 | 2 | 2 |
| 100 | RPS4X | 29.6 | 115 | 14 | 3 | 3 |
| 101 | H2BC20P | 21.5 | 112 | 17 | 2 | 3 |
| 102 | PSIP1 | 60.1 | 110 | 5 | 2 | 2 |
| 103 | OTX1 | 37.3 | 109 | 7 | 2 | 2 |
| 104 | YLPM1 | 241.5 | 105 | 2 | 4 | 4 |
| 105 | RPL17 | 21.4 | 103 | 26 | 4 | 4 |
| 106 | DSP | 331.6 | 102 | 1 | 3 | 3 |
| 107 | RPS27A | 18 | 96 | 18 | 2 | 2 |
| 108 | RPL24 | 17.8 | 96 | 14 | 2 | 2 |
| 109 | CHD1L | 100.9 | 92 | 3 | 2 | 2 |
| 110 | SRSF1 | 27.7 | 91 | 10 | 2 | 2 |
| 111 | PLS3 | 70.8 | 86 | 5 | 3 | 3 |
| 112 | RPS13 | 17.2 | 82 | 18 | 2 | 2 |
| 113 | HSP90AB1 | 83.2 | 78 | 5 | 3 | 3 |
| 114 | ARPC5L | 16.9 | 77 | 25 | 3 | 3 |
| 115 | BIRC5 | 16.4 | 76 | 36 | 3 | 3 |
| 116 | RPS2 | 31.3 | 73 | 12 | 3 | 3 |
| 117 | PNN | 81.6 | 69 | 4 | 2 | 2 |
| 118 | TCP1 | 60.3 | 68 | 5 | 2 | 2 |
| 119 | CDCA8 | 31.3 | 66 | 10 | 2 | 2 |
| 120 | RPS7 | 22.1 | 65 | 18 | 2 | 2 |
| 121 | NEXN | 80.6 | 64 | 3 | 2 | 2 |
| 122 | COL3A1 | 138.5 | 64 | 2 | 2 | 2 |
| 123 | ERH | 12.3 | 62 | 16 | 2 | 2 |
| 124 | YWHAZ | 27.7 | 59 | 7 | 2 | 2 |
| 125 | IGF2BP1 | 63.4 | 59 | 4 | 2 | 2 |
| 126 | RPS20 | 13.4 | 57 | 19 | 2 | 2 |
| 127 | RPL36AL | 12.5 | 57 | 16 | 2 | 2 |
| 128 | GRN | 63.5 | 54 | 4 | 2 | 2 |
| 129 | EIF4A1 | 46.1 | 54 | 6 | 2 | 2 |
| 130 | RPS11 | 18.4 | 40 | 12 | 2 | 2 |

Table S4. LKB1 potential ubiquitination modification sites identified by LC-MS in Fig. 4a and supplementary Fig. S5

| **Accession** | **MW (kDa)** | **Protein Score** | **Sequence Coverage** | **Position** | **Peptide Score** | **Modified**  **Sequence** | **Mass Error (ppm)** | **# PSMs** |
| --- | --- | --- | --- | --- | --- | --- | --- | --- |
| STK11 | 49.04 | 323.31 | 74.40% | K44 | 185.41 | AKLIGKYLMGDLLGEGSYGK | -3.2989 | 4 |
| STK11 | 49.04 | 323.31 | 74.40% | K48 | 185.41 | LIGKYLMGDLLGEGSYGKVK | 0.35277 | 2 |
| STK11 | 49.04 | 323.31 | 74.40% | K62 | 207.55 | YLMGDLLGEGSYGKVKEVLDSETLCR | 0.53165 | 1 |
| STK11 | 49.04 | 323.31 | 74.40% | K403 | 297.7 | GLPKAVCMNGTEAAQLSTKSR | 0.30142 | 3 |
| STK11 | 49.04 | 323.31 | 74.40% | K178 | 100.04 | DIKPGNLLLTTGGTLK | 0.30397 | 3 |
| STK11 | 49.04 | 323.31 | 74.40% | K191 | 253.06 | DIKPGNLLLTTGGTLK*ISDLGVAEALHPFAADDTCR | 0.38714 | 5 |
| STK11 | 49.04 | 323.31 | 74.40% | K388 | 219.99 | RGLPKAVCMNGTEAAQLSTK | -0.70759 | 7 |
| STK11 | 49.04 | 323.31 | 74.40% | K296 | 148.91 | GSYAIPGDCGPPLSDLLKGMLEYEPAKR | 1.0066 | 4 |
| STK11 | 49.04 | 323.31 | 74.40% | K287 | 152.88 | GSYAIPGDCGPPLSDLLKGMLEYEPAKR | 1.0066 | 4 |
| STK11 | 49.04 | 323.31 | 74.40% | K108 | 334.43 | HKNVIQLVDVLYNEEK | -1.3339 | 9 |
| STK11 | 49.04 | 323.31 | 74.40% | K122 | 299.58 | NVIQLVDVLYNEEKQK | -0.74512 | 6 |
| STK11 | 49.04 | 323.31 | 74.40% | K329 | 89.468 | HPPAEAPVPIPPSPDTKDR | -0.84238 | 12 |
| STK11 | 49.04 | 323.31 | 74.40% | K96 | 93.424 | RIPNGEANVKKEIQLLR | -0.39333 | 1 |
| STK11 | 49.04 | 323.31 | 74.40% | K97 | 93.424 | RIPNGEANVKKEIQLLR | -0.39333 | 1 |
| STK11 | 49.04 | 323.31 | 74.40% | K312 | 118.23 | KKHPPAEAPVPIPPSPDTK | -0.2784 | 1 |
| STK11 | 49.04 | 323.31 | 74.40% | K311 | 118.23 | KKHPPAEAPVPIPPSPDTK | -0.2784 | 1 |
| STK11 | 49.04 | 323.31 | 74.40% | K269 | 215.08 | LFENIGKGSYAIPGDCGPPLSDLLK | 0.3458 | 5 |
| STK11 | 49.04 | 323.31 | 74.40% | K64 | 117.83 | YLMGDLLGEGSYGKVKEVLDSETLCR | 0.53165 | 1 |
| STK11 | 49.04 | 323.31 | 74.40% | K146 | 214.14 | MYMVMEYCVCGMQEMLDSVPEKR | 0.16627 | 3 |
| **Accession** | **MW (kDa)** | **Protein Score** | **Sequence Coverage** | **Position** | **Peptide Score** | **Modified Sequence** | **Mass Error (ppm)** | **# PSMs** |
| STK11 | 49.04 | 102 | 64.20% | K178 | 34.38 | DIK*PGNLLLTTGGTLK | 20ppm | 2 |
| STK11 | 49.04 | 102 | 64.20% | K97 | 37.69 | K*EIQLLR | 20ppm | 2 |
| STK11 | 49.04 | 102 | 64.20% | K312 | 12.26 | K*HPPAEAPVPIPPSPDTK | 20ppm | 1 |
| STK11 | 49.04 | 102 | 64.20% | K269 | 16.4 | K.LFENIGK*.G | 20ppm | 1 |
| STK11 | 49.04 | 102 | 64.20% | K48 | 21.17 | LIGK*YLMGDLLGEGSYGK | 20ppm | 1 |
| STK11 | 49.04 | 102 | 64.20% | K122 | 31.66 | NVIQLVDVLYNEEK*QK | 20ppm | 1 |
| STK11 | 49.04 | 102 | 64.20% | K64 | 43.73 | VK*EVLDSETLCR | 20ppm | 1 |
| STK11 | 49.04 | 102 | 64.20% | K48 | 7.41 | AK*LIGK*YLMGDLLGEGSYGK | 20ppm | 1 |
| STK11 | 49.04 | 102 | 64.20% | K44 | 18.34 | AK*LIGK | 20ppm | 3 |
| **Accession** | **MW (kDa)** | **Protein Score** | **Sequence Coverage** | **Position** | **Peptide Score** | **Modified Sequence** | **Mass Error (ppm)** | **# PSMs** |
| STK11 | 49.04 | 6945.17 | 67.73% | K 122 | 91.1 | NVIQLVDVLYNEEkQK | -1.47 | 4 |
| STK11 | 49.04 | 6945.17 | 67.73% | K 48 | 82.67 | LIGkYLMGDLLGEGSYGK | 0.56 | 4 |
| STK11 | 49.04 | 6945.17 | 67.73% | K 108 | 61.57 | HkNVIQLVDVLYNEEK | 6.52 | 4 |
| STK11 | 49.04 | 6945.17 | 67.73% | K 64 | 55.37 | VkEVLDSETLcR | -1.56 | 2 |
| STK11 | 49.04 | 6945.17 | 67.73% | K 97 | 52.55 | kEIQLLR | -1.60 | 1 |
| STK11 | 49.04 | 6945.17 | 67.73% | K 62 | 51.91 | YLmGDLLGEGSYGkVK | 3.99 | 4 |
| STK11 | 49.04 | 6945.17 | 67.73% | K 296 | 42.71 | GmLEYEPAkR | -1.99 | 1 |
| STK11 | 49.04 | 6945.17 | 67.73% | K 178 | 40.2 | DIkPGNLLLTTGGTLK | -1.19 | 2 |
| STK11 | 49.04 | 6945.17 | 67.73% | K 423 | 34.87 | KAcSASSk*IR | 0.02 | 3 |
| STK11 | 49.04 | 6945.17 | 67.73% | K 329 | 33.93 | HPPAEAPVPIPPSPDTkDR | -2.48 | 1 |
| STK11 | 49.04 | 6945.17 | 67.73% | K 78 | 25.26 | AVkILK | 0.17 | 1 |
| STK11 | 49.04 | 6945.17 | 67.73% | K 312 | 25.08 | KkHPPAEAPVPIPPSPDTK | -1.89 | 4 |
| STK11 | 49.04 | 6945.17 | 67.73% | K 44 | 24.61 | AkLIGK | -0.38 | 1 |
| STK11 | 49.04 | 6945.17 | 67.73% | K 296 | 20.91 | GMLEYEPAkR | -1.14 | 1 |

**Table S5. The average RTV and tumor weight of PDXs**

| **PDX** | **Group** | **RTV** | **T/C** | **Tumor Weight (g)** | **Inhibition ratio** |
| --- | --- | --- | --- | --- | --- |
| LUAD 110519 (Case 1) | Scramble | 8.46 ± 3.13 | - | 0.41 ± 0.16 | - |
|  | shJOSD2#1 | 3.20 ± 1.37** | 37.84% | 0.14 ±0.07** | 64.73% |
| LUAD 083120 (Case 2) | Scramble | 4.24 ± 1.19 | - | 0.16 ± 0.07 | - |
|  | shJOSD2#1 | 2.73 ± 0.63* | 64.34% | 0.08 ± 0.02* | 52.24% |

*: *vs.* Scramble: *P* < 0.05; **: *vs.* Scramble: *P* < 0.01.

Table S6. The list of compounds for JOSD2 inhibitor screening, related to Fig. 7a

| **Number** | **Compounds** | **Concentration**  **(μM)** | **JOSD2 Activity Inhibition (%)** | **Source of compounds** |
| --- | --- | --- | --- | --- |
| 1 | PR-619 | 2 | 42.70% | DUB inhibitor |
| 2 | EOAI3402143 | 2 | 29.05% | DUB inhibitor |
| 3 | DUBs-IN-1 | 2 | 15.67% | DUB inhibitor |
| 4 | DUBs-IN-2 | 2 | 5.01% | DUB inhibitor |
| 5 | DUBs-IN-3 | 2 | 14.15% | DUB inhibitor |
| 6 | SJB2-043 | 2 | 13.43% | DUB inhibitor |
| 7 | SJB3-019A | 2 | 42.78% | DUB inhibitor |
| 8 | GW7647 | 2 | -29.69% | DUB inhibitor |
| 9 | USP7-IN-1 | 2 | -29.77% | DUB inhibitor |
| 10 | AC-17 (Carbazochrome sodium sulfonate) | 2 | 9.85% | DUB inhibitor |
| 11 | LDN-57444 | 2 | -16.75% | DUB inhibitor |
| 12 | C527 | 2 | 24.99% | DUB inhibitor |
| 13 | GNE-6640 | 2 | 12.54% | DUB inhibitor |
| 14 | Ubiquitin Isopeptidase Inhibitor I | 2 | 1.00% | DUB inhibitor |
| 15 | HBX 19818 | 2 | -0.37% | DUB inhibitor |
| 16 | Vigabatrin Hydrochloride | 2 | -16.37% | clinically-used drug |
| 17 | LCZ696 | 2 | -19.26% | clinically-used drug |
| 18 | Tyloxapol | 2 | 29.65% | clinically-used drug |
| 19 | Pargyline | 2 | 29.80% | clinically-used drug |
| 20 | Etamsylate | 2 | -21.36% | clinically-used drug |
| 21 | Procainamide hydrochloride | 2 | -20.45% | clinically-used drug |
| 22 | Molsidomine | 2 | 35.78% | clinically-used drug |
| 23 | Roxatidine Acetate hydrochloride | 2 | -20.82% | clinically-used drug |
| 24 | Pranoprofen | 2 | -2.73% | clinically-used drug |
| 25 | Nylidrin hydrochloride | 2 | -31.95% | clinically-used drug |
| 26 | Ticagrelor | 2 | 23.06% | clinically-used drug |
| 27 | Prednisolone phosphate sodium | 2 | 36.74% | clinically-used drug |
| 28 | Amlodipine Besylate | 2 | -24.34% | clinically-used drug |
| 29 | Amcinonide | 2 | -19.24% | clinically-used drug |
| 30 | Dimenhydrinate | 2 | -24.96% | clinically-used drug |
| 31 | Methylprodnisolone Sodium Succinate | 2 | -78.62% | clinically-used drug |
| 32 | Canrenone | 2 | -22.34% | clinically-used drug |
| 33 | Aminoguanidine hydrochloride | 2 | -35.00% | clinically-used drug |
| 34 | Leucobasal | 2 | -15.32% | clinically-used drug |
| 35 | Diammonium Glycyrrhizinate | 2 | -22.76% | clinically-used drug |
| 36 | Metyrapone | 2 | -6.94% | clinically-used drug |
| 37 | Pioglitazone hydrochloride | 2 | -5.00% | clinically-used drug |
| 38 | Medrysone | 2 | -8.83% | clinically-used drug |
| 39 | Ergotamine bitartrate | 2 | -5.18% | clinically-used drug |
| 40 | Risedronate Sodium | 2 | 4.34% | clinically-used drug |
| 41 | Capsaicin | 2 | 1.77% | clinically-used drug |
| 42 | Ketaneserin | 2 | -29.69% | clinically-used drug |
| 43 | Rasagiline | 2 | 28.97% | clinically-used drug |
| 44 | Betamethasone 17-valerate | 2 | -26.02% | clinically-used drug |
| 45 | Papaverine hydrochloride | 2 | -27.95% | clinically-used drug |
| 46 | Clopidogrel sulfate | 2 | -14.96% | clinically-used drug |
| 47 | CH011804 | 2 | 11.38% | synthesized small molecule |
| 48 | ZY011106 | 2 | 11.16% | synthesized small molecule |
| 49 | CH011501 | 2 | 13.63% | synthesized small molecule |
| 50 | ZY010506 | 2 | 10.22% | synthesized small molecule |
| 51 | HY040205 | 2 | 38.63% | synthesized small molecule |
| 52 | HY034901 | 2 | 38.52% | synthesized small molecule |
| 53 | HY034503 | 2 | 35.51% | synthesized small molecules |
| 54 | WL014202 | 2 | 30.88% | synthesized small molecule |
| 55 | ZY013301 | 2 | -14.73% | synthesized small molecule |
| 56 | ZY013602 | 2 | 33.05% | synthesized small molecule |
| 57 | HY041004 | 2 | 86.14% | synthesized small molecule |
| 58 | HY04071 | 2 | 63.02% | synthesized small molecule |
| 59 | HY040305 | 2 | 34.65% | synthesized small molecule |
| 60 | WL012803 | 2 | 5.49% | synthesized small molecule |
| 61 | HY042903 | 2 | -3.48% | synthesized small molecule |
| 62 | HY021621 | 2 | 1.70% | synthesized small molecules |
| 63 | HY021907 | 2 | 6.31% | synthesized small molecule |
| 64 | WL013002 | 2 | -3.29% | synthesized small molecule |
| 65 | HY023203 | 2 | -8.31% | synthesized small molecule |
| 66 | HY050101 | 2 | 9.59% | synthesized small molecule |
| 67 | HY013317 | 2 | 12.04% | synthesized small molecule |
| 68 | HY018708 | 2 | 8.62% | synthesized small molecule |
| 69 | HY021205 | 2 | 11.21% | synthesized small molecule |
| 70 | HY031511 | 2 | 12.28% | synthesized small molecule |
| 71 | HY022603 | 2 | 10.54% | synthesized small molecules |
| 72 | XL024202 | 2 | 9.45% | synthesized small molecule |
| 73 | XL023107 | 2 | 8.19% | synthesized small molecule |
| 74 | HY033205 | 2 | 36.51% | synthesized small molecule |
| 75 | HY041702 | 2 | 34.73% | synthesized small molecule |
| 76 | HY030213 | 2 | 16.85% | synthesized small molecule |
| 77 | HY012508 | 2 | -18.55% | synthesized small molecule |
| 78 | WL012301 | 2 | -18.63% | synthesized small molecule |
| 79 | HY033901 | 2 | 16.77% | synthesized small molecule |
| 80 | HY031004 | 2 | 4.38% | synthesized small molecules |
| 81 | HY031809 | 2 | -34.24% | synthesized small molecule |
| 82 | HY032607 | 2 | -49.01% | synthesized small molecule |
| 83 | XL022802 | 2 | -12.93% | synthesized small molecule |
| 84 | HY032204 | 2 | -21.14% | synthesized small molecule |
| 85 | HY022401 | 2 | -12.64% | synthesized small molecule |
| 86 | HY024804 | 2 | 24.65% | synthesized small molecule |
| 87 | WJ13002 | 2 | -21.24% | synthesized small molecule |
| 88 | HY052113 | 2 | -18.12% | synthesized small molecule |
| 89 | KT53504 | 2 | 39.84% | synthesized small molecules |
| 90 | HY032801 | 2 | -0.98% | synthesized small molecule |
| 91 | HY030804 | 2 | 4.80% | synthesized small molecule |
| 92 | HY023402 | 2 | 9.25% | synthesized small molecule |
| 93 | HY023902 | 2 | -16.00% | synthesized small molecule |
| 94 | XT010718 | 2 | -44.71% | synthesized small molecule |
| 95 | XT010726 | 2 | -39.77% | synthesized small molecule |
| 96 | XT010749 | 2 | -66.41% | synthesized small molecule |
| 97 | XL034402 | 2 | -54.94% | synthesized small molecule |
| 98 | XL034601 | 2 | -45.55% | synthesized small molecules |
| 99 | XL043108 | 2 | -15.14% | synthesized small molecule |

**Table S7. The average RTV and tumor weight of NCI-H1299 xenograft**

| **Group** | **RTV** | **T/C** | **Tumor Weight (g)** | **Inhibition ratio** |
| --- | --- | --- | --- | --- |
| Ctrl | 27.83 ± 3.61 | - | 2.40 ± 0.41 | - |
| HY041004 (50 mg/kg) | 18.01 ± 7.33* | 64.71% | 1.18 ± 0.49*** | 50.83% |
| HY041004 (100 mg/kg) | 12.21 ± 2.94*** | 43.88% | 0.90 ± 0.23*** | 62.50% |

*: *vs.* Ctrl: *P* < 0.05; ***: *vs.* Ctrl: *P* < 0.001.

**Table S8. The sequence for gene-specific shRNA.**

| Genes | Forward (5’ - 3’) | Reverse (5’ - 3’) |
| --- | --- | --- |
| shJOSD2#1 | GCAACTATGATGTCAATGTGA | TCACATTGACATCATAGTTGC |
| shJOSD2#2 | CCAGGTGGACGGTGTCTACTA | TAGTAGACACCGTCCACCTGG |
| shJOSD1 | TTGCGAGGAAAGAACTGTGAA | TTCACAGTTCTTTCCTCGCAA |
| shUSP22 | AGCTACCAGGAGTCCACAAAG | CTTTGTGGACTCCTGGTAGCT |
| shUSP32 | GCACTGATGATATTCCTGAAT | ATTCAGGAATATCATCAGTGC |
| shUSP38 | GCCTTCAAGTACAGCCTTCTT | AAGAAGGCTGTACTTGAAGGC |
| shUSP12 | GCACCAATAATGCTTTGAATT | AATTCAAAGCATTATTGGTGC |
| shUSP47 | CGGAATCATGTTGTGCACTAT | ATAGTGCACAACATGATTCCG |
| shUSP43 | CCATCGCAGAGGGAGATAATG | CATTATCTCCCTCTGCGATGG |
| shUSP7 | CCAGCTAAGTATCAAAGGAAA | TTTCCTTTGATACTTAGCTGG |
| shUSP33 | CGAGGTTATTCTCAGCAGGAT | ATCCTGCTGAGAATAACCTCG |
| shSTAMBPL1 | GCTGCTACTCTAAGTGCTGTT | AACAGCACTTAGAGTAGCAGC |

**Table S9. The primers sequence for site-directed mutagenesis.**

| Name | Forward (5’ - 3’) | Reverse (5’ - 3’) |
| --- | --- | --- |
| JOSD2-C24A-HA | CGCCTGGAGCTGGCTGCTGTCCACGC | GCGTGGACAGCAGCCAGCTCCAGGCG |
| JOSD2-H125Y-HA | CTGCGCCGGCGGTACTGGGTGGCCC | GGGCCACCCAGTACCGCCGGCGCAG |
| LKB1-K78I-Flag | GCAGGAGGGCCGTCATAATCCTCAAGAAGAAG | CTTCTTCTTGAGGATTATGACGGCCCTCCTGC |
| LKB1-K191R-Flag | GGTGGCACCCTCAGAATCTCCGACCTG | CAGGTCGGAGATTCTGAGGGTGCCACC |
| LKB1-K269R-Flag | GTTTGAGAACATCGGGAGGGGGAGCTACGCCATCC | GGATGGCGTAGCTCCCCCTCCCGATGTTCTCAAAC |
| LKB1-K423R-Flag | CCGCCAGCAGCAGGATCCGCCGGCTG | CAGCCGGCGGATCCTGCTGCTGGCGG |
| LKB1-K191-Flag | GGTGGCACCCTCAAAATCTCCGACCTG | CAGGTCGGAGATTTTGAGGGTGCCACC |
| LKB1-K269-Flag | GTTTGAGAACATCGGGAAGGGGAGCTACGCCATCC | GGATGGCGTAGCTCCCCTTCCCGATGTTCTCAAAC |
| LKB1-K423-Flag | GCTCCGCCAGCAGCAAGATCCGCCGGCTGTC | GACAGCCGGCGGATCTTGCTGCTGGCGGAGC |
| LKB1-K78R-Flag | CTGTGCAGGAGGGCCGTCAGGATCCTCAAGAAGAA | CTTCTTCTTCTTGAGGATCCTGACGGCCCTCCTGCACAG |
| LKB1-K84R-Flag | CCTCAAGAAGAAGAGGTTGCGAAGGATCC | GGATCCTTCGCAACCTCTTCTTCTTGAGG |
| LKB1-K96R-Flag | GGAGGCCAACGTGAGGAAGGAAATTCAAC | GTTGAATTTCCTTCCTCACGTTGGCCTCC |
| LKB1-K97R-Flag | GAGGCCAACGTGAAGAGGGAAATTCAACTACTG | CAGTAGTTGAATTTCCCTCTTCACGTTGGCCTC |
| LKB1-K108R-Flag | GAGGTTACGGCACAGAAATGTCATCCAGC | GCTGGATGACATTTCTGTGCCGTAACCTC |
| LKB1-K122R-Flag | GTTATACAACGAAGAGAGGCAGAAAATGTATATGG | CCATATACATTTTCTGCCTCTCTTCGTTGTATAAC |
| LKB1-K124R-Flag | CAACGAAGAGAAGCAGAGAATGTATATGGTGATGG | CCATCACCATATACATTCTCTGCTTCTCTTCGTTG |
| LKB1-K146R-Flag | GCGTGCCGGAGAGGCGTTTCCCAGTG | CACTGGGAAACGCCTCTCCGGCACGC |
| LKB1-K175-178R-Flag | GGCATTGTGCACAGGGACATCAGGCCGGGGAACCTGC | CAGCAGGTTCCCCGGCCTGATGTCCCTGTGCACAATGC |
| LKB1-K235R-Flag | CTTCTCCGGCTTCAGGGTGGACATCTGGTC | GACCAGATGTCCACCCTGAAGCCGGAGAAG |
| LKB1-K262R-Flag | GGGGACAACATCTACAGGTTGTTTGAGAACATC | GATGTTCTCAAACAACCTGTAGATGTTGTCCCC |
| LKB1-K287R-Flag | CTCTCTGACCTGCTGAGAGGGATGCTTGAGTAC | GTACTCAAGCATCCCTCTCAGCAGGTCAGAGAG |
| LKB1-K296R-Flag | GTACGAACCGGCCAGGAGGTTCTCCATCC | GGATGGAGAACCTCCTGGCCGGTTCGTAC |
| LKB1-K311-312R-Flag | CAGCTGGTTCCGGAGGAGGCATCCTCCGGCTGAAGC | GTGCTTCAGCCGGAGGATGCCTCCTCCGGAACCAGCTG |
| LKB1-K329R-Flag | GCCCAGACACCAGGGACCGGTGGCG | CGCCACCGGTCCCTGGTGTCTGGGC |
| LKB1-K388R-Flag | CGGGGCCTCCCCAGGGCCGTGTGTATG | CATACACACGGCCCTGGGGAGGCCCCG |
| LKB1-K403R-Flag | CAGCTGAGCACCAGATCCAGGGCGGAG | CTCCGCCCTGGATCTGGTGCTCAGCTG |

**References**

1 Aguirre-Gamboa, R. *et al.* SurvExpress: an online biomarker validation tool and database for cancer gene expression data using survival analysis. *PLoS One* **8**, e74250 (2013).

2 Altun, M. *et al.* Activity-based chemical proteomics accelerates inhibitor development for deubiquitylating enzymes. *Chem Biol* **18**, 1401-1412 (2011).

3 Liu, J. *et al.* C. N. I. P. Administration, Ed. (China, 2019), vol. CN11021898198A.

4 Martinez Molina, D. *et al.* Monitoring drug target engagement in cells and tissues using the cellular thermal shift assay. *Science* **341**, 84-87 (2013).
