# Supplementary material for: Josephin domain containing 2 (JOSD2) promotes lung cancer by inhibiting LKB1 (Liver kinase B1) activity
Source: Signal Transduct Target Ther. 2024 Jan 5;9:11. doi: 10.1038/s41392-023-01706-y (PMC10766984; doi:10.1038/s41392-023-01706-y)
Supplement: Supplementary file 2 — Raw Data of Statistical Analysis [file 41392_2023_1706_MOESM2_ESM.pdf]

Raw Data of Statistical Analysis

Figure 1d

Number	TNM	Position	Positive proportion score	Dyeing strength score	Score			*P* value
1	IA	A15	1.00	1.00	1.00		I vs II	0.00623
2	IA	B10	1.00	1.00	1.00		I vs III	0.00015
3	IA	C2	2.00	2.00	4.00		I vs IV	0.00002
4	IA	D12	3.00	2.00	6.00		II vs III	0.53328
5	IA	D6	2.00	2.00	4.00		II vs IV	0.02281
6	IA	E1	2.00	1.00	2.00		III vs IV	0.05472
7	IA	E2	3.00	2.00	6.00			
8	IB	A1	1.00	1.00	1.00			
9	IB	A16	1.00	1.00	1.00			
10	IB	A9	2.00	2.00	4.00			
11	IB	B4	1.00	1.00	1.00			
12	IB	C15	2.00	2.00	4.00			
13	IB	D13	1.00	1.00	1.00			
14	IB	D3	1.00	1.00	1.00			
15	IB	D5	2.00	1.00	2.00			
16	IB	D9	2.00	1.00	2.00			
17	IB	E13	1.00	1.00	1.00			
18	IB	E4	1.00	1.00	1.00			
19	IB	E9	1.00	1.00	1.00			
20	II	C14	1.00	1.00	1.00			
21	IIA	B2	1.00	2.00	2.00			
22	IIA	B9	2.00	3.00	6.00			
23	IIA	C3	3.00	3.00	9.00			
24	IIA	C8	4.00	2.00	8.00			
25	IIA	E3	1.00	2.00	2.00			
26	IIB	A10	2.00	3.00	6.00			
27	IIB	A11	2.00	2.00	4.00			
28	IIB	A14	2.00	3.00	6.00			
29	IIB	B3	2.00	2.00	4.00			
30	IIB	B5	2.00	1.00	2.00			
31	IIB	C13	4.00	3.00	12.00			
32	IIB	C4	2.00	1.00	2.00			
33	IIB	C5	1.00	1.00	1.00			
34	IIB	D1	1.00	1.00	1.00			
35	IIB	D15	4.00	2.00	8.00			
36	IIB	E11	3.00	2.00	6.00			
37	IIB	E5	2.00	2.00	4.00			
38	IIB	E6	2.00	3.00	6.00			
39	III	A3	1.00	3.00	3.00			
40	III	A4	2.00	3.00	6.00			
41	III	A5	3.00	3.00	9.00			
42	III	A7	2.00	2.00	4.00			
43	III	B11	3.00	3.00	9.00			
44	IIIA	A12	1.00	2.00	2.00			
45	IIIA	A2	1.00	2.00	2.00			
46	IIIA	A6	1.00	1.00	1.00			
47	IIIA	B12	3.00	2.00	6.00			
48	IIIA	B7	3.00	2.00	6.00			
49	IIIA	C10	1.00	2.00	2.00			
50	IIIA	C7	2.00	3.00	6.00			
51	IIIA	C9	3.00	3.00	9.00			
52	IIIA	D11	4.00	3.00	12.00			
53	IIIA	D16	2.00	3.00	6.00			
54	IIIA	D4	2.00	2.00	4.00			
55	IIIA	D7	2.00	2.00	4.00			
56	IIIA	D8	2.00	2.00	4.00			
57	IIIA	E10	2.00	3.00	6.00			
58	IIIA	E12	1.00	1.00	1.00			
59	IIIA	E14	2.00	3.00	6.00			
60	IIIA	E8	4.00	2.00	8.00			
61	IIIB	A13	4.00	2.00	8.00			
62	IIIB	B6	1.00	1.00	1.00			
63	IIIB	D14	4.00	2.00	8.00			
64	IV	A8	1.00	1.00	1.00			
65	IV	B1	4.00	3.00	12.00			
66	IV	B13	3.00	3.00	9.00			
67	IV	B14	4.00	3.00	12.00			
68	IV	B15	2.00	1.00	2.00			
69	IV	B16	4.00	2.00	8.00			
70	IV	B8	3.00	3.00	9.00			
71	IV	C1	2.00	2.00	4.00			
72	IV	C11	3.00	2.00	6.00			
73	IV	C12	2.00	2.00	4.00			
74	IV	C16	3.00	2.00	6.00			
75	IV	C6	4.00	3.00	12.00			
76	IV	D10	4.00	2.00	8.00			
77	IV	D2	4.00	3.00	12.00			
78	IV	E15	4.00	2.00	8.00			
79	IV	E16	2.00	2.00	4.00			
80	IV	E7	3.00	3.00	9.00			

Figure 1e

NCI-H1299	scramble			shJOSD2#1			shJOSD2#2		
day0	1	1	1	1	1	1	1	1	1
day2	3.078	2.931	3.156	1.308	1.223	1.096	1.421	1.297	1.554
day3	7.091	6.841	7.349	1.550	1.444	1.387	1.728	1.535	1.910
day4	13.260	14.252	15.387	1.356	1.632	1.571	1.418	1.352	1.833
day5	19.733	21.254	22.882	1.171	1.197	1.283	1.154	1.208	1.669
		P value							
	scramble vs shJOSD2#1	0.002							
	scramble vs shJOSD2#2	0.002							

PC-9	scramble			shJOSD2#1			shJOSD2#2		
day0	1.000	1.000	1.000	1.000	1.000	1.000	1.000	1.000	1.000
day2	2.933	2.877	2.844	1.554	1.324	1.362	1.568	1.626	1.356
day3	6.353	5.787	5.923	1.654	1.561	1.627	1.618	1.809	1.620
day5	13.332	12.792	14.068	1.484	1.491	1.638	1.637	1.581	1.644
day7	21.450	21.480	21.616	1.122	1.119	1.094	1.420	1.253	1.219
		P value							
	scramble vs shJOSD2#1	3.38E-06							
	scramble vs shJOSD2#2	2.78E-09							

H2347	scramble			shJOSD2#1			shJOSD2#2		
day0	1.000	1.000	1.000	1.000	1.000	1.000	1.000	1.000	1.000
day1	1.091	1.220	1.185	0.790	0.989	0.800	0.987	0.949	0.951
day2	1.621	1.803	1.721	0.826	1.029	0.789	1.033	0.941	0.975
day3	2.670	2.907	2.607	0.858	1.080	0.835	1.064	0.962	1.065
day4	4.049	4.485	4.176	0.875	1.081	0.871	1.122	1.013	1.035
day5	5.104	5.685	4.857	0.875	1.142	0.869	1.081	1.052	0.974
		P value							
	scramble vs shJOSD2#1	0.001							
	scramble vs shJOSD2#2	0.003							

H2228	scramble			shJOSD2#1			shJOSD2#2		
day0	1.000	1.000	1.000	1.000	1.000	1.000	1.000	1.000	1.000
day1	1.290	1.247	1.352	1.048	1.070	1.069	1.091	0.991	0.991
day2	1.875	1.734	1.937	0.989	1.080	0.958	0.958	0.880	0.880
day3	2.781	2.563	3.177	1.006	1.081	1.018	1.026	0.857	0.857
day4	4.636	4.274	4.134	1.025	1.107	0.841	1.018	0.828	0.828
day5	5.282	5.754	5.636	1.106	1.236	1.073	1.220	0.948	0.948
		P value							
	scramble vs shJOSD2#1	0.00031							
	scramble vs shJOSD2#2	0.00004							

H292	scramble			shJOSD2#1			shJOSD2#2		
day0	1.000	1.000	1.000	1.000	1.000	1.000	1.000	1.000	1.000
day1	1.253	1.234	1.454	1.188	1.152	1.067	1.055	1.150	1.157
day2	1.306	1.217	1.557	1.059	0.974	0.997	1.128	1.034	0.925
day3	1.610	1.346	1.922	1.192	1.132	1.112	1.250	1.114	1.098
day4	2.063	2.015	2.285	1.282	1.072	1.051	1.141	1.030	1.041
day5	2.518	2.135	2.657	1.321	1.032	1.052	1.142	1.029	1.032
day6	2.760	2.062	3.171	1.182	1.043	1.048	1.151	0.990	1.034
day7	3.445	2.464	3.189	1.139	1.124	1.103	1.170	1.053	1.067
day8	3.844	4.327	3.833	1.162	1.109	1.138	1.092	1.055	1.089
day9	4.900	4.805	5.313	1.199	1.105	1.140	1.082	1.056	1.089
		P value							
	scramble vs shJOSD2#1	0.001							
	scramble vs shJOSD2#2	0.002							

H358	scramble			shJOSD2#1			shJOSD2#2		
day0	1.000	1.000	1.000	1.000	1.000	1.000	1.000	1.000	1.000
day3	2.038	2.015	1.889	1.145	1.146	1.037	1.167	1.051	1.072
day6	4.773	4.333	4.078	1.142	1.207	1.076	1.121	0.974	1.152
day9	9.082	8.124	7.823	0.909	0.786	1.001	0.806	0.853	0.946
		P value							
	scramble vs shJOSD2#1	0.002							
	scramble vs shJOSD2#2	0.002							

Figure 1h

Tumor Volume (mm^3)	Ctrl									
Day 1	0	0	0	0	0	0	0	0	0	0
Day12	67.01	64.43	87.12	97.09	76.91	85.26	66.16	146.37	62.07	104.74
Day 18	122.39	97.49	151.82	127.24	101.47	123.3	110.93	197.15	102.38	129.53
Day 21	210.78	120.38	172.58	197.82	135.77	164.39	126.36	317.53	128.05	190.91
Day 24	363.73	214.46	220.41	222.29	153.15	224.8	156.84	359.73	143.34	236.61
Day 27	404.59	238.64	245.03	229.48	269.46	286.17	207.77	416.69	174.6	189.81
Day 30	449.29	340.42	247.9	396.64	348.48	506.05	296.42	421.28	261.79	224.2
Day 33	544.6	466.69	255.62	603.04	442.43	709.9	404.91	434.49	391.71	252.8
Day 37	788.14	576.81	293.86	1210.85	728.97	1037.52	652.39	655.05	428.41	512.29
average	688.429									
Tumor Volume (mm^3)	shJOSD2									
Day 1	0	0	0	0	0	0	0	0	0	0
Day12	0	0	0	0	0	0	0	0	0	0
Day 18	0	0	0	0	0	0	0	0	0	0
Day 21	0	0	0	0	0	0	0	0	0	0
Day 24	0	0	0	0	0	0	0	0	0	0
Day 27	0	0	0	0	0	0	0	0	0	0
Day 30	0	0	0	0	0	0	0	0	0	0
Day 33	0	0	0	0	0	0	0	0	0	0
Day 37	0	0	0	0	0	0	0	0	0	0
average	0									
P value	2.34E-05									

Figure 1i

	Ctrl									
Tumor weight (g)	0.2552	0.2217	0.1902	0.1843	0.2098	0.2392	0.193	0.0944	0.1688	0.0762
Average	0.1833									
	shJOSD2									
Tumor weight (g)	0	0	0	0	0	0	0	0	0	0
Average	0									
P value	3.56E-06									

Figure 1k

NCI-H1299 xenograft	Tumor weight（g）	average	Inhibition	*P* value
Scramble	0.1108	0.0807	0.5972	0.00006
	0.0995			
	0.0892			
	0.0769			
	0.0703			
	0.0726			
	0.0606			
	0.0654			
shJOSD2	0.0598	0.0325		
	0.0434			
	0.0466			
	0.0347			
	0.0238			
	0.0206			
	0.0193			
	0.0117			

Figure 5a

NCI-H1299	Vector			JOSD2		
	replicate-1	replicate-2	replicate-3	replicate-1	replicate-2	replicate-3
0	1.000	1.000	1.000	1.000	1.000	1.000
1	1.040	1.392	1.025	0.982	1.115	1.033
2	1.242	1.180	1.253	1.195	1.289	1.251
3	1.322	1.304	1.342	1.354	1.692	1.516
4	1.793	1.748	1.834	2.089	2.744	2.284
5	2.586	2.596	2.598	4.052	5.897	4.540
6	4.091	4.125	4.411	6.220	8.279	6.951
avarage	4.209			7.150		
SD	0.176			1.044		
P value	0.0363					
PC-9	Vector			JOSD2		
	replicate-1	replicate-2	replicate-3	replicate-1	replicate-2	replicate-3
0	1.000	1.000	1.000	1.000	1.000	1.000
1	1.100	1.168	1.081	1.140	1.245	1.214
2	1.389	1.531	1.444	1.525	1.695	1.667
3	1.878	1.835	1.837	2.044	2.477	2.437
4	2.348	2.422	2.272	3.592	4.081	3.759
5	3.673	4.061	3.446	7.237	6.563	6.993
6	5.539	5.476	5.310	11.017	9.948	10.913
avarage	5.442			10.626		
SD	0.118			0.589		
P value	0.0032					

Figure 5b

	A549					
	PCDH			JOSD2-HA		
	replicate1	replicate2	replicate3	replicate1	replicate2	replicate3
day0	1.000	1.000	1.000	1.000	1.000	1.000
day1	1.129	1.072	0.995	1.023	1.038	1.023
day2	1.218	1.248	1.228	1.185	1.229	1.207
day3	1.223	1.311	1.208	1.239	1.183	1.065
day4	2.188	2.517	1.960	1.663	2.015	1.540
day5	4.521	5.142	3.509	3.076	3.330	2.942
day6	7.804	8.262	6.916	5.969	6.797	5.123
avarage	7.661			5.963		
SD	0.684			0.837		
P value	0.0552					

	H460					
	PCDH			JOSD2-HA		
	replicate1	replicate2	replicate3	replicate1	replicate2	replicate3
day0	1.000	1.000	1.000	1.000	1.000	1.000
day1	0.933	0.900	1.066	0.988	0.796	0.899
day2	1.117	1.215	1.338	1.124	0.867	1.025
day3	1.244	1.342	1.589	1.619	1.347	1.496
day4	2.075	2.392	3.345	2.933	3.302	3.041
day5	3.974	5.375	7.009	5.898	6.808	6.037
day6	6.114	8.782	10.770	7.765	9.108	8.111
avarage	8.555			8.328		
SD	2.336			0.697		
P value	0.8847					

Figure 5f

	Ctrl				
	Replicate 1	Replicate 2	Replicate 3	Replicate 4	Replicate 5
Day 1	1.000	1.000	1.000	1.000	1.000
Day 3	1.201	1.152	2.198	2.305	2.223
Day 5	2.325	2.225	3.152	4.695	3.297
Day 7	3.860	3.537	7.039	7.671	7.413
Day 8	7.775	8.400	8.837	9.154	9.064
Average	8.646				
	LKB1				
	Replicate 1	Replicate 2	Replicate 3	Replicate 4	Replicate 5
Day 1	1.000	1.000	1.000	1.000	1.000
Day 3	1.208	1.173	2.067	2.091	2.062
Day 5	2.504	2.229	2.682	2.702	2.707
Day 7	3.343	3.332	4.542	4.760	5.072
Day 8	7.186	7.822	5.663	5.972	5.998
Average	6.528				
	3KR				
	Replicate 1	Replicate 2	Replicate 3	Replicate 4	Replicate 5
Day 1	1.000	1.000	1.000	1.000	1.000
Day 3	1.178	1.183	2.359	2.311	2.346
Day 5	2.476	2.455	3.694	3.694	3.728
Day 7	3.973	3.856	7.870	8.446	8.725
Day 8	8.261	7.482	10.353	10.214	10.559
Average	9.374				
	Ctrl+JOSD2				
	Replicate 1	Replicate 2	Replicate 3	Replicate 4	Replicate 5
Day 1	1.000	1.000	1.000	1.000	1.000
Day 3	1.128	1.172	2.160	2.328	2.228
Day 5	2.038	2.237	3.349	3.711	3.625
Day 7	2.746	3.225	7.217	8.234	8.116
Day 8	5.995	6.541	9.421	10.313	11.873
Average	8.829				
	LKB1+JOSD2				
	Replicate 1	Replicate 2	Replicate 3	Replicate 4	Replicate 5
Day 1	1.000	1.000	1.000	1.000	1.000
Day 3	1.847	1.950	2.650	3.067	3.089
Day 5	8.185	8.176	4.173	5.030	5.032
Day 7	16.328	17.511	8.584	9.441	9.890
Day 8	22.687	21.000	12.170	12.412	13.851
Average	16.424				
	3KR+JOSD2				
	Replicate 1	Replicate 2	Replicate 3	Replicate 4	Replicate 5
Day 1	1.000	1.000	1.000	1.000	1.000
Day 3	1.175	1.259	2.443	2.117	2.104
Day 5	2.345	2.950	3.663	3.246	3.269
Day 7	3.927	5.152	8.138	7.803	7.678
Day 8	7.467	8.257	10.005	9.775	9.398
Average	8.980				
			P value		
	Ctrl vs LKB1		0.0038		
	Ctrl vs 3KR		0.3295		
	Ctrl vs Ctrl+JOSD2		0.8808		
	LKB1 vs LKB1+3KR		0.0107		
	3KR vs 3KR+JOSD2		0.6339		

Figure 5i

Tumor Volume (mm^3)	Ctrl						
Day 1	0	0	0	0	0	0	0
Day 7	143.92	297.31	312.18	225.69	194.33	284.03	182.2
Day 9	182.21	340.49	371.46	256.95	233.56	420.62	274.04
Day 11	212.83	447.74	422.73	315.87	258.94	483.57	362
Day 14	225.74	462.52	425.76	321.03	271.26	498.81	386.61
Day 17	290.68	514.16	372.29	375.03	334.54	607.6	412.53
average	415.2614286						
Tumor Volume (mm^3)	LKB1						
Day 1	0	0	0	0	0	0	0
Day 7	132.85	168.71	149.98	130.96	156.73	255.27	157.88
Day 9	134.64	170.39	156.11	140.79	166.32	274.36	172.56
Day 11	140.13	194.66	169.88	159.97	178.3	299.84	201.51
Day 14	144.62	221.97	182.7	163.73	189.16	309.13	210.76
Day 17	153.93	280.89	202.13	171.92	214.97	324.1	224.23
average	224.5957143						
Tumor Volume (mm^3)	3KR						
Day 1	0	0	0	0	0	0	0
Day 7	193.58	215.1	142.87	124.06	293.2	149.99	189.01
Day 9	331.11	272.65	151.01	159.14	318.5	248.59	235.62
Day 11	411.28	298.08	160.59	171.01	327.32	277.51	238.64
Day 14	694.67	340.53	169.49	235.83	377.4	312.6	270.13
Day 17	776.27	375.07	223.94	324.94	400.42	270.45	302.68
average	381.9671429						
Tumor Volume (mm^3)	Ctrl+JOSD2						
Day 1	0	0	0	0	0	0	0
Day 7	228.97	269.87	226.05	207.85	230.21	275.81	215.63
Day 9	264	283.8	266.51	261.26	249.84	363.53	333.66
Day 11	282.62	371.81	286.83	361.42	259.75	418.28	378.86
Day 14	308.34	479.68	289.64	375.42	300.09	458.37	395.56
Day 17	365.68	622.32	298.25	413.84	330.34	481.98	420.4
average	418.9728571						
Tumor Volume (mm^3)	LKB1+JOSD2						
Day 1	0	0	0	0	0	0	0
Day 7	270.91	289.35	334.99	251.79	325.78	349.83	212.94
Day 9	405.48	345.72	334.66	373.05	408.1	471.5	269.16
Day 11	455.49	379.57	377	391.63	455.9	502.37	298.98
Day 14	543.5	428.07	463.86	461.89	521.82	551.41	372.85
Day 17	561.04	455.4	498.16	467.44	531.52	573.43	415.35
average	500.3342857						
Tumor Volume (mm^3)	3KR+JOSD2						
Day 1	0	0	0	0	0	0	0
Day 7	198.79	248.71	235.91	234.11	196.75	147.14	256.62
Day 9	309.58	278.13	282.83	257.69	211.42	160.38	306.43
Day 11	334.19	299.79	359.88	272.69	245.38	220.42	306.43
Day 14	393.77	348.87	422.99	292.72	269.16	244.62	335.6
Day 17	325.83	420.33	442.7	306.31	292.15	416.8	377.79
average	368.8442857						
		P value					
Ctrl vs LKB1		0.002771					
Ctrl vs 3KR		0.689643					
Ctrl vs Ctrl+JOSD2		0.950314					
LKB1 vs LKB1+JOSD2		0.000002					
3KR vs 3KR+JOSD2		0.862569					

Figure 5j

	Number	tumor weight（g）	average（g）
Ctrl	1	0.181	0.0684
	2	0.074	
	3	0.0539	
	4	0.0532	
	5	0.0403	
	6	0.039	
	7	0.0373	
LKB1	1	0.0215	0.0152
	2	0.021	
	3	0.0195	
	4	0.012	
	5	0.0114	
	6	0.0109	
	7	0.01	
3KR	1	0.1039	0.0594
	2	0.0796	
	3	0.0753	
	4	0.0433	
	5	0.0426	
	6	0.0405	
	7	0.0309	
Ctrl+JOSD2	1	0.1455	0.0922
	2	0.1339	
	3	0.0918	
	4	0.0758	
	5	0.0721	
	6	0.0658	
	7	0.0608	
LKB1+JOSD2	1	0.1746	0.1223
	2	0.1371	
	3	0.1317	
	4	0.1191	
	5	0.102	
	6	0.1017	
	7	0.0899	
3KR+JOSD2	1	0.106	0.0656
	2	0.0944	
	3	0.0848	
	4	0.0497	
	5	0.0447	
	6	0.0399	
	7	0.0395	
	P value		
Ctrl vs LKB1	0.03343		
Ctrl vs 3KR	0.69235		
Ctrl vs Ctrl+JOSD2	0.32811		
LKB1 vs LKB1+JOSD2	0.00005		
3KR vs 3KR+JOSD2	0.68636		

Figure 6b

LUAD110519	scramble			shJOSD2#1			shJOSD2#2		
	replicate-1	replicate-2	replicate-3	replicate-1	replicate-2	replicate-3	replicate-1	replicate-2	replicate-3
day 0	1.000	1.000	1.000	1.000	1.000	1.000	1.000	1.000	1.000
day 2	1.674	1.563	1.825	1.056	0.978	0.965	1.359	1.140	0.841
day 4	1.851	2.225	2.759	1.289	1.018	1.083	1.376	1.174	0.892
day 6	2.671	2.667	2.749	1.286	1.078	1.393	1.155	1.183	1.097
day 8	2.973	2.988	3.011	1.352	1.192	1.661	1.058	1.424	1.204
day 10	3.339	3.400	3.640	1.663	1.509	1.533	1.069	1.707	1.238
day 12	4.963	3.988	4.356	1.953	1.470	1.841	1.232	1.705	1.150
avarage	4.436			1.755			1.362		
SD	0.493			0.253			0.299		
		P value							
	scramble vs shJOSD2#1	0.0036							
	scramble vs shJOSD2#2	0.0018							

LUAD050820	scramble			shJOSD2#1			shJOSD2#2		
	replicate-1	replicate-2	replicate-3	replicate-1	replicate-2	replicate-3	replicate-1	replicate-2	replicate-3
day 0	1.000	1.000	1.000	1.000	1.000	1.000	1.000	1.000	1.000
day 2	1.688	1.245	1.142	0.997	1.178	1.205	1.101	1.311	1.171
day 4	2.293	1.812	1.642	1.120	0.924	0.829	1.136	0.985	0.889
day 6	2.696	2.421	2.248	1.245	1.228	1.226	1.140	1.329	1.209
day 8	2.991	2.761	2.515	1.397	1.546	1.218	1.210	1.565	1.221
day 10	3.464	3.474	3.177	1.563	1.310	1.410	1.301	1.502	1.285
day 12	4.418	4.160	3.918	1.738	1.220	1.143	1.324	1.189	1.027
		P value							
	scramble vs shJOSD2#1	0.0004							
	scramble vs shJOSD2#2	0.0002							

Figure 6d-6e

LUAD 110519	Number	Body weight (g)	Tumor weight (g)	tumor weight	scramble vs shJOSD2
scramble	1	21.9	0.6129	*P* value	0.0035
	2	20.6	0.4377	body weight	scramble vs shJOSD2
	3	17.2	0.2744	*P* value	0.6318
	4	21.6	0.4628		
	5	20.4	0.1773		
	6	21.8	0.5754		
	7	17.8	0.3291		
shJOSD2	1	18.6	0.2639		
	2	19.4	0.0827		
	3	21.5	0.1278		
	4	19.5	0.1556		
	5	19.5	0.0526		
	6	20.2	0.155		
	7	19.8	0.1744		
LUAD 083120	Number	Body weight (g)	Tumor weight (g)	tumor weight	scramble vs shJOSD2
scramble	1	22	0.1954	*P* value	0.0497
	2	22.6	0.2638	body weight	scramble vs shJOSD2
	3	23.8	0.1082	*P* value	0.6765
	4	21.1	0.1568		
	5	22.2	0.0914		
shJOSD2	1	21.8	0.0576		
	2	23.9	0.0822		
	3	23.6	0.0972		
	4	21.6	0.0534		
	5	22.2	0.0991		

Figure 6f

LUAD 110915								LUAD 110915							
tumor volume (mm^3)	Scramble							tumor volume (mm^3)	shJOSD2						
day 0	50.78	52.14	58.05	54.03	42.9	51.75	60.15	day 0	57.49	59.77	50.6	47.94	27.68	53.36	33.7
day 2	100.71	59.61	57.98	91.43	59.65	68.89	78.95	day 2	32.15	52.46	37.99	44.13	24.7	41.2	39.15
day 4	127.6	117.74	108.12	100.96	67.47	73.07	94.94	day 4	58.53	49.53	45.72	44.89	22.27	48.87	49.82
day 6	170.53	128.42	108.02	114.93	70.4	120.23	119.13	day 6	66.68	34.44	70.59	71.12	32.36	53.92	60.59
day 8	252.12	166.64	106.02	139.14	121.85	140.83	179.71	day 8	95.24	41.12	82.97	92.69	25.87	50.33	53.29
day 10	335.7	237.27	141.76	198.87	137.26	181.06	228.95	day 10	109.72	27.89	55.57	95.92	31.97	94.79	89.58
day 12	470.48	265.37	165.83	202.54	138.76	286.72	277.9	day 12	171.98	34.34	65.16	101.8	41.58	99.15	95.09
day 14	541.56	297.72	214.32	265.1	158.46	331.2	320.57	day 14	150.34	45.58	75.15	110.67	30.64	98.88	103.78
day 16	515.62	415.61	213.04	350.11	152.58	338.37	312.72	day 16	158.75	68.6	81.2	103.74	41.15	209.95	102.63
day 18	561.32	452.56	337.77	447.92	166.53	498.12	346.2	day 18	173.19	90.66	109.12	128.17	52.18	230.63	115.22
day 20	577.85	577.68	345.76	494.44	180.54	607.53	341.18	day 20	204.01	89.97	108.37	159.35	57.79	272.67	157.61
avarage	446.43						149.97								
			P value												
scramble vs shJOSD2			0.00192												

LUAD 083120						LUAD 083120									
tumor volume (mm^3)	Scramble					tumor volume (mm^3)	shJOSD2								
day 0	60.39	47.12	44.53	27.33	27.8	day 0	24.03	36.34	32.52	18.45	24.38				
day 2	72.42	78.39	66.72	49.84	30.8	day 2	27.93	44.91	57.25	19.87	21.49				
day 4	96.09	133.49	71.25	56.53	33.47	day 4	29.78	59.16	53.36	30.1	19.53				
day 6	105.24	199.25	101.63	83.58	47.69	day 6	31.08	46.99	55.85	36.64	44.11				
day 8	150.83	203.42	118.01	70.57	56.54	day 8	30.35	64.64	58.82	28	35.99				
day 10	172.94	225.9	169.7	66.9	62.34	day 10	38.64	67.6	73.35	25.08	41.65				
day 12	181.93	243.17	193.94	63.63	77.33	day 12	43.9	65.8	68.99	42.99	68.31				
day 14	226.62	294.47	186.93	107.33	86	day 14	54.62	104.94	91.39	37.46	89.07				
avarage	180.27						75.496								
			P value												
scramble vs shJOSD2			0.04998												

Figure 8c

H1299 xenograft																		
Tumor Volume	Ctrl						HY041004 50 mg/kg						HY041004 100 mg/kg					
	1.0	2.0	3.0	4.0	5.0	6.0	1.0	2.0	3.0	4.0	5.0	6.0	1.0	2.0	3.0	4.0	5.0	6.0
0.000	52.48	54.48	81.79	53.67	47.50	57.84	47.23	44.28	41.24	30.84	57.66	61.28	53.98	51.58	46.35	56.25	50.43	56.23
2.000	254.93	251.24	215.97	226.92	162.70	138.12	120.13	67.55	58.88	127.84	117.37	75.89	58.68	64.39	58.07	128.59	119.09	173.59
4.000	387.73	541.45	446.91	430.38	423.12	235.95	216.89	153.07	101.82	216.63	365.02	88.41	74.70	139.22	71.66	165.31	163.61	308.14
6.000	748.79	1331.72	1063.14	1017.26	972.26	479.24	285.95	277.14	189.62	602.27	392.12	169.09	183.18	330.83	181.26	505.10	333.41	597.90
8.000	1067.62	1537.06	1538.43	1193.65	1425.60	902.30	504.49	476.16	585.20	643.97	968.47	399.61	296.26	533.31	321.73	629.52	467.34	815.63
10.000	1368.59	1638.02	2123.45	1318.70	1623.15	1510.50	801.44	704.91	762.64	896.74	1214.92	401.60	518.83	527.62	483.69	857.93	562.66	932.20

Relative Tumor Volume	Ctrl						HY041004 50 mg/kg						HY041004 100 mg/kg					
	1.00	2.00	3.00	4.00	5.00	6.00	1.00	2.00	3.00	4.00	5.00	6.00	1.00	2.00	3.00	4.00	5.00	6.00
0.000	1.00	1.00	1.00	1.00	1.00	1.00	1.00	1.00	1.00	1.00	1.00	1.00	1.00	1.00	1.00	1.00	1.00	1.00
2.000	4.86	4.61	2.64	4.23	3.42	2.39	2.54	1.53	1.43	4.15	2.04	1.24	1.09	1.25	1.25	2.29	2.36	3.09
4.000	7.39	9.94	5.46	8.02	8.91	4.08	4.59	3.46	2.47	7.02	6.33	1.44	1.38	2.70	1.55	2.94	3.24	5.48
6.000	14.27	24.44	13.00	18.95	20.47	8.29	6.06	6.26	4.60	19.53	6.80	2.76	3.39	6.41	3.91	8.98	6.61	10.63
8.000	20.34	28.21	18.81	22.24	30.01	15.60	10.68	10.75	14.19	20.88	16.80	6.52	5.49	10.34	6.94	11.19	9.27	14.51
10.000	26.08	30.06	25.96	24.57	34.17	26.12	16.97	15.92	18.49	29.08	21.07	6.55	9.61	10.23	10.43	15.25	11.16	16.58

Relative Tumor Volume	Ctrl	HY041004 50 mg/kg	HY041004 100 mg/kg
AVERAGE	27.826	18.013	12.210
STDEV	3.614	7.331	2.942
P value		0.02070	0.00001

Figure 8d

	Ctrl						HY041004 50 mg/kg						HY041004 100 mg/kg					
Tumor Weight （g）	2.129	2.583	2.633	2.300	2.959	1.817	0.634	1.241	1.167	1.175	2.062	0.807	0.891	0.940	0.617	1.218	1.055	0.684
Tumor Weight/g	Ctrl	HY041004 50 mg/kg	HY041004 100 mg/kg															
AVERAGE	2.404	1.181	0.901															
STDEV	0.406	0.494	0.225															
P value		0.00095	0.00005															

Supplementary Figure 2b

	Ctrl			shJOSD2#1			shJOSD2#2		
	Replicated 1	Replicated 2	Replicated 3	Replicated 1	Replicated 2	Replicated 3	Replicated 1	Replicated 2	Replicated 3
DAY1	1.000	1.000	1.000	1.000	1.000	1.000	1.000	1.000	1.000
DAY2	2.032	2.238	2.296	1.574	1.653	1.823	1.316	2.045	1.904
DAY3	3.174	3.282	3.745	1.810	1.800	2.461	1.146	1.708	1.970
DAY4	5.441	6.078	6.257	2.429	2.906	3.313	1.803	2.573	2.989
DAY5	9.931	10.793	12.110	4.283	4.274	4.906	2.715	4.014	4.820
DAY6	15.233	19.209	18.001	12.542	12.726	13.197	10.650	15.092	13.445
		P value							
	Ctrl vs shJOSD2#1	0.0548							
	Ctrl vs shJOSD2#2	0.0657							

Supplementary Figure 2c

NCI-H1299 Colony Formation Assay			
Raw	Scramble	shJOSD2#1	shJOSD2#2
replicate-1	1.000	0.038	0.043
replicate-2	1.000	0.020	0.039
replicate-3	1.000	0.026	0.031
P value	Scramble vs shJOSD2#1	2.99E-05	
	Scramble vs shJOSD2#2	1.40E-05	

NCI-H2347 Colony Formation Assay			
Raw	Scramble	shJOSD2#1	shJOSD2#2
replicate-1	1.000	0.002	0.002
replicate-2	1.000	0.001	0.002
replicate-3	1.000	0.001	0.002
P value	Scramble vs shJOSD2#1	6.98E-08	
	Scramble vs shJOSD2#2	7.58E-08	

PC-9 Colony Formation Assay			
Raw	Scramble	shJOSD2#1	shJOSD2#2
replicate-1	1.000	0.050	0.059
replicate-2	1.000	0.022	0.041
replicate-3	1.000	0.032	0.038
P value	Scramble vs shJOSD2#1	7.46E-05	
	Scramble vs shJOSD2#2	4.71E-05	

NCI-H2228 Colony Formation Assay			
Raw	Scramble	shJOSD2#1	shJOSD2#2
replicate-1	1.000	0.001	0.002
replicate-2	1.000	0.001	0.002
replicate-3	1.000	0.000	0.001
P value	Scramble vs shJOSD2#1	2.27E-08	
	Scramble vs shJOSD2#2	8.16E-08	

NCI-H292 Colony Formation Assay			
Raw	Scramble	shJOSD2#1	shJOSD2#2
replicate-1	1.000	0.000	0.000
replicate-2	1.000	0.000	0.000
replicate-3	1.000	0.000	0.000
P value	Scramble vs shJOSD2#1	1.27E-11	
	Scramble vs shJOSD2#2	1.43E-10	

NCI-H358 Colony Formation Assay			
Raw	Scramble	shJOSD2#1	shJOSD2#2
replicate-1	1.000	0.063	0.074
replicate-2	1.000	0.034	0.042
replicate-3	1.000	0.036	0.041
P value	Scramble vs shJOSD2#1	9.51E-05	
	Scramble vs shJOSD2#2	1.30E-04	

Supplementary Figure 3f

	replicate-1	replicate-2	replicate-3	Average	*P* value	
Scramble	1.000	1.000	1.000	1.000		
shJOSD2#1	3.048	3.358	2.778	3.061	0.007	*vs.* Scramble
shJOSD2#2	2.400	2.641	3.166	2.736	0.017	*vs.* Scramble

Supplementary Figure 3g

	p-LKB1	JOSD2		r value
NCI-H1299	0.412	1.490		-0.7512
PC-9	1.269	0.744		*P* value
NCI-H2228	1.000	0.875		0.0031
NCI-H2347	0.854	0.324		
NCI-H292	1.747	0.359		
NCI-H358	0.396	1.229		
NCI-H226	0.769	1.157		
NCI-H1650	0.748	1.170		
NCI-H1373	1.147	0.530		
Calu-1	0.856	0.729		
MDA-MB-231	0.499	0.765		
CAPAN-2	0.517	1.000		
PANC-1	0.073	1.354		
	p-AMPK	JOSD2		r value
NCI-H1299	0.437	1.490		-0.7851
PC-9	2.094	0.744		*P* value
NCI-H2228	1.264	0.875		0.0015
NCI-H2347	1.000	0.324		
NCI-H292	2.061	0.359		
NCI-H358	0.301	1.229		
NCI-H226	0.245	1.157		
NCI-H1650	0.768	1.170		
NCI-H1373	1.563	0.530		
Calu-1	1.493	0.729		
MDA-MB-231	1.429	0.765		
CAPAN-2	0.742	1.000		
PANC-1	0.253	1.354		
	p-LKB1	p-AMPK		r value
NCI-H1299	0.412	0.437		0.8197
PC-9	1.269	2.094		*P* value
NCI-H2228	1.000	1.264		0.0006
NCI-H2347	0.854	1.000		
NCI-H292	1.747	2.061		
NCI-H358	0.396	0.301		
NCI-H226	0.769	0.245		
NCI-H1650	0.748	0.768		
NCI-H1373	1.147	1.563		
Calu-1	0.856	1.493		
MDA-MB-231	0.499	1.429		
CAPAN-2	0.517	0.742		
PANC-1	0.073	0.253		

Supplementary Figure 3h

	replicate-1	replicate-2	replicate-3	Average	*P* value	
GST	1.000	1.000	1.000	1.000		
GST-JOSD2	0.384	0.462	0.389	0.412	0.0019	*vs.* GST
GST-C24A	0.836	0.869	0.872	0.859	0.0008	*vs.* GST-JOSD2
GST-H125Y	0.892	0.916	0.931	0.913	0.0006	*vs.* GST-JOSD2
GST-CA&HY	0.948	0.974	1.003	0.975	0.0002	*vs.* GST-JOSD2

Supplementary Figure 4a

	replicate-1	replicate-2	replicate-3	Average	*P* value
GST	1.000	1.000	1.000	1.000	
GST-JOSD2	0.362	0.305	0.440	0.369	0.0038

Supplementary Figure 4c

	replicate-1	replicate-2	replicate-3	Average	*P* value	
Vector	1.000	1.000	1.000	1.000		
JOSD2	0.160	0.201	0.152	0.171	0.0003	*vs.* Vector
C24A	0.645	0.835	0.638	0.706	0.0109	*vs.* JOSD2
H125Y	0.774	1.276	0.766	0.939	0.0439	*vs.* JOSD2
CA&HY	1.319	1.396	1.071	1.262	0.0069	*vs.* JOSD2

Supplementary Figure 4d

	replicate-1	replicate-2	replicate-3	Average	*P* value	
GST	1.000	1.000	1.000	1.000	0.0127	GST-JOSD2 *vs.* GST
GST-JOSD2	0.188	0.377	0.068	0.211	0.0343	GST-JOSD2 *vs.* GST-C24A
GST-C24A	1.421	0.847	0.834	1.034	0.8766	GST *vs.* GST-C24A

Supplementary Figure 6c

	A549					
	replicate-1	replicate-2	replicate-3	Average	*P* value	
Vector	1.000	1.000	1.000	1.000		
LKB1-WT	4.738	4.746	4.970	4.818	0.0004	*vs.* Vector
LKB1-3KR	2.398	2.401	2.495	2.431	0.0002	*vs.* LKB1-WT
	H460					
	replicate-1	replicate-2	replicate-3	Average	*P* value	
Vector	1.000	1.000	1.000	1.000		
LKB1-WT	6.256	6.268	6.586	6.370	0.0004	*vs.* Vector
LKB1-3KR	2.634	2.637	2.720	2.664	0.0004	*vs.* LKB1-WT

Supplementary Figure 6d

	STRAD					
	replicate-1	replicate-2	replicate-3	Average	*P* value	
Vector						
LKB1	1	1	1	1		
LKB1-3KR	0.464	0.414	0.401	0.426	0.0011	*vs.* LKB1
LKB1-KR	0.000	0.000	0.000	0.000	0.0020	*vs.* 3KR
	MO25					
	replicate-1	replicate-2	replicate-3	Average	*P* value	
Vector						
LKB1	1.000	1.000	1.000	1.000		
LKB1-3KR	0.636	0.568	0.568	0.590	0.0030	*vs.* LKB1
LKB1-KR	0.000	0.000	0.000	0.000	0.0015	*vs.* 3KR

Supplementary Figure 6f

	STRAD						
	replicate-1	replicate-2	replicate-3	Average	*P* value		
Vector							
LKB1-WT	1.000	1.000	1.000	1.000	0.0003	LKB1-WT *vs.* LKB1-3KR	
LKB1-3KR	0.353	0.316	0.345	0.338	0.0003	LKB1-WT *vs.* LKB1-WT+JOSD2	
Vector+JOSD2							
LKB1-WT+JOSD2	0.488	0.457	0.481	0.475	0.0004	LKB1-WT+JOSD2 *vs.* LKB1-3KR+JOSD2	
LKB1-3KR+JOSD2	0.313	0.274	0.304	0.297	0.0650	LKB1-3KR *vs.* LKB1-3KR+JOSD2	
	MO25						
	replicate-1	replicate-2	replicate-3	Average	*P* value		
Vector							
LKB1-WT	1.000	1.000	1.000	1.000	0.0014	LKB1-WT *vs.* LKB1-3KR	
LKB1-3KR	0.283	0.190	0.261	0.245	0.0017	LKB1-WT *vs.* LKB1-WT+JOSD2	
Vector+JOSD2							
LKB1-WT+JOSD2	0.439	0.359	0.421	0.406	0.0153	LKB1-WT+JOSD2 *vs.* LKB1-3KR+JOSD2	
LKB1-3KR+JOSD2	0.293	0.202	0.272	0.256	0.7952	LKB1-3KR *vs.* LKB1-3KR+JOSD2	

Supplementary Figure 6g

	STRAD					
	replicate-1	replicate-2	replicate-3	Average	*P* value	
Vector	1.000	1.000	1.000	1.000		
JOSD2	0.248	0.238	0.130	0.205	0.0022	*vs.* Vector
C24A	0.450	0.443	0.369	0.420	0.0124	*vs.* JOSD2
H125Y	0.797	0.794	0.764	0.785	0.0024	*vs.* JOSD2
CA&HY	1.380	1.567	1.658	1.535	0.0010	*vs.* JOSD2
	MO25					
	replicate-1	replicate-2	replicate-3	Average	*P* value	
Vector	1.000	1.000	1.000	1.000		
JOSD2	0.169	0.141	0.095	0.135	0.0006	*vs.* Vector
C24A	0.461	0.445	0.414	0.440	0.0007	*vs.* JOSD2
H125Y	0.838	1.015	1.015	0.956	0.0022	*vs.* JOSD2
CA&HY	2.070	2.107	1.720	1.966	0.0036	*vs.* JOSD2

Supplementary Figure 6h

	STRAD					
	replicate-1	replicate-2	replicate-3	Average	*P* value	
-	0.000	0.000	0.000	0.000		
Ctrl+LKB1	1.000	1.000	1.000	1.000		
shJOSD2+LKB1	2.210	2.988	2.080	2.426	0.0373	*vs.* Ctrl+ LKB1
	MO25					
	replicate-1	replicate-2	replicate-3	Average	*P* value	
-	0	0	0	0		
Ctrl+LKB1	1.000	1.000	1.000	1.000		
shJOSD2+LKB1	2.332	2.245	1.596	2.058	0.0449	*vs.* Ctrl+ LKB1

Supplementary Figure 6i

LKB1-Flag						LKB1-3KR-Flag				
Number	All	Nuclear	Cytoplasm	Nuc/Cyt		Number	All	Nuclear	Cytoplasm	Nuc/Cyt
1	293.95	116.89	177.07	0.66		1.00	162.38	119.85	42.53	2.82
2	395.60	93.00	302.60	0.31		2.00	142.65	90.13	52.53	1.72
3	338.38	110.89	227.49	0.49		3.00	156.81	107.28	49.53	2.17
4	210.24	25.87	184.38	0.14		4.00	228.72	142.83	85.89	1.66
5	260.97	114.63	146.34	0.78		5.00	120.22	64.30	55.93	1.15
6	919.14	313.43	605.71	0.52		6.00	144.86	82.51	62.35	1.32
7	525.49	203.25	322.24	0.63		7.00	121.55	86.46	35.09	2.46
8	504.99	117.62	387.37	0.30		8.00	131.49	100.55	30.93	3.25
9	782.27	138.39	643.88	0.21		9.00	144.09	109.84	34.25	3.21
10	612.89	219.84	393.05	0.56		10.00	188.91	117.48	71.43	1.64
11	295.55	97.42	198.13	0.49		11.00	171.68	109.13	62.56	1.74
12	413.81	105.84	307.97	0.34		12.00	181.88	133.46	48.41	2.76
13	373.58	99.41	274.17	0.36		13.00	187.33	123.77	63.56	1.95
14	232.07	59.89	172.18	0.35		14.00	125.73	38.56	87.17	0.44
15	242.42	70.35	172.08	0.41		15.00	225.06	132.76	92.30	1.44
16	160.20	63.43	96.77	0.66		16.00	125.25	95.59	29.66	3.22
17	217.21	42.28	174.93	0.24		17.00	328.00	218.21	109.79	1.99
18	372.15	62.40	309.75	0.20		18.00	312.75	176.98	135.77	1.30
19	428.95	117.99	310.96	0.38		19.00	96.99	57.13	39.86	1.43
20	386.47	98.10	288.37	0.34		20.00	103.26	87.52	15.74	5.56
21	332.45	90.14	242.31	0.37		21.00	122.13	92.74	29.39	3.16
22	202.72	80.36	122.36	0.66		22.00	205.66	167.88	37.78	4.44
23	192.46	51.83	140.63	0.37		23.00	160.79	130.73	30.06	4.35
24	306.21	90.83	215.38	0.42		24.00	148.74	111.02	37.72	2.94
25	415.04	133.22	281.83	0.47		25.00	174.11	116.68	57.43	2.03
26	395.73	83.92	311.81	0.27		26.00	148.74	118.11	30.63	3.86
27	286.72	52.25	234.47	0.22		27.00	160.68	130.56	30.12	4.34
28	260.53	81.64	178.89	0.46		28.00	153.15	110.13	43.02	2.56
29	171.33	37.29	134.04	0.28		29.00	246.36	153.98	92.38	1.67
30	266.86	104.66	162.19	0.65		30.00	211.80	165.42	46.38	3.57
31	567.19	87.23	479.96	0.18		31.00	127.35	107.16	20.19	5.31
32	373.22	53.02	320.20	0.17		32.00	207.82	157.06	50.76	3.09
33	115.34	15.26	100.08	0.15		33.00	159.20	120.07	39.13	3.07
34	163.08	62.79	100.29	0.63		34.00	167.87	121.18	46.69	2.60
35	134.24	71.60	62.64	1.14		35.00	121.30	73.84	47.47	1.56
36	211.27	42.45	168.82	0.25		36.00	121.77	88.93	32.84	2.71
37	276.01	75.48	200.53	0.38		37.00	142.13	100.89	41.25	2.45
38	277.42	49.86	227.56	0.22		38.00	135.82	108.23	27.59	3.92
39	495.23	34.22	461.01	0.07		39.00	232.35	182.59	49.76	3.67
40	141.22	14.80	126.43	0.12		40.00	246.26	170.76	75.50	2.26
41	180.21	49.64	130.58	0.38		41.00	134.34	108.80	25.54	4.26
42	180.93	19.63	161.30	0.12		42.00	100.19	84.19	15.99	5.26
43	324.24	139.51	184.73	0.76		43.00	133.48	123.52	9.96	12.40
44	540.10	160.80	379.30	0.42		44.00	151.26	132.41	18.85	7.03
45	448.03	64.21	383.82	0.17		45.00	169.72	132.02	37.69	3.50
46	251.20	17.63	233.57	0.08		46.00	120.68	98.85	21.83	4.53
47	201.48	21.09	180.39	0.12		47.00	122.19	91.99	30.20	3.05
48	246.65	32.92	213.73	0.15		48.00	111.42	87.05	24.38	3.57
49	247.59	52.94	194.65	0.27		49.00	100.80	60.42	40.38	1.50
50	370.83	48.97	321.86	0.15		50.00	130.97	70.83	60.14	1.18
Average				0.369						3.061
SD				0.218						1.898
									P value	1.7E-13

Supplementary Figure 7a

	vector	JOSD2	C24A
Repeat-1	1.000	2.056	1.213
Repeat-2	1.000	1.801	1.106
Repeat-3	1.000	2.100	1.126
Average	1.000	1.986	1.149
P value		0.0088	JOSD2 vs. vector
		0.0068	JOSD2 vs. C24A

Supplementary Figure 7b

Tumor volume (mm^3)	Vector (EGFP)							
Day 1	0.00	0.00	0.00	0.00	0.00	0.00	0.00	0.00
Day 18	30.01	69.11	101.82	106.24	40.27	61.45	65.04	100.26
Day 22	34.21	48.97	78.47	123.26	46.49	72.41	84.98	119.27
Day 27	67.11	97.79	102.93	202.89	119.35	112.68	99.27	218.73
Day 31	122.59	160.93	217.43	248.72	153.32	117.81	137.62	230.54
Day 34	152.03	103.49	255.37	279.36	219.77	163.87	254.47	251.46
Day 37	249.01	189.91	335.60	390.36	287.12	243.55	357.40	346.02
Average	299.87							
Tumor volume (mm^3)	JOSD2							
Day 1	0.00	0.00	0.00	0.00	0.00	0.00	0.00	0.00
Day 18	175.77	93.51	85.34	142.93	92.95	57.96	64.98	65.19
Day 22	227.68	122.87	121.08	169.49	116.51	71.15	81.58	82.60
Day 27	401.23	197.44	223.99	245.95	180.93	95.80	135.26	138.07
Day 31	657.40	271.68	385.25	446.24	369.47	165.89	273.98	262.08
Day 34	787.12	339.06	403.65	621.09	428.47	190.18	394.19	370.83
Day 37	912.32	614.77	635.56	788.65	460.56	346.58	553.93	943.03
Average	656.93							
Tumor volume (mm^3)	C24A							
Day 1	0.00	0.00	0.00	0.00	0.00	0.00	0.00	0.00
Day 18	197.61	141.86	40.18	77.98	65.47	195.20	46.41	41.84
Day 22	232.29	188.46	44.91	100.62	79.26	238.49	66.02	55.79
Day 27	368.66	256.84	67.52	132.46	115.65	396.79	101.58	77.07
Day 31	432.25	294.11	69.64	163.85	148.17	502.19	142.68	233.98
Day 34	532.67	422.66	110.40	218.37	248.37	600.41	197.68	366.60
Day 37	683.49	492.19	197.01	286.43	350.24	626.68	248.13	451.85
Average	417.00							
		P value						
Vector vs. JOSD2		0.0016						
C24A vs. JOSD2		0.0279						
Vector vs. C24A		0.1153						

Supplementary Figure 7c

	Vector (EGFP)							
Tumor weight (g)	0.078	0.052	0.086	0.079	0.069	0.052	0.052	0.090
Average	0.070							
	JOSD2							
Tumor weight (g)	0.223	0.243	0.149	0.154	0.130	0.070	0.107	0.172
Average	0.156							
	C24A							
Tumor weight (g)	0.121	0.060	0.054	0.078	0.080	0.158	0.076	0.153
Average	0.097							
		P value						
Vector (EGFP) vs. JOSD2		0.0033						
C24A vs. JOSD2		0.0353						
Vector (EGFP) vs. C24A		0.1086						

Supplementary Figure 7d-e

	Vector			LKB1			Vector-shJOSD2			LKB1-shJOSD2		
	Repeat-1	Repeat-2	Repeat-3	Repeat-1	Repeat-2	Repeat-3	Repeat-1	Repeat-2	Repeat-3	Repeat-1	Repeat-2	Repeat-3
Day 0	1	1	1	1	1	1	1	1	1	1	1	1
Day 1	1.27	1.38	1.32	1.29	1.23	1.23	1.04	1.10	1.17	1.14	1.13	1.16
Day 3	2.45	2.43	2.43	1.82	1.87	1.80	1.24	1.20	1.25	1.07	1.11	1.09
Day 5	4.96	5.40	5.76	3.15	3.00	2.79	1.74	1.71	1.80	1.09	1.05	1.12
Day 7	13.67	19.28	17.80	9.64	9.39	9.25	4.66	4.52	3.53	1.61	1.08	1.33
Day 9	25.31	30.39	26.82	17.58	23.59	18.59	8.34	11.23	8.32	2.06	1.15	1.11
Average		27.51			19.92			9.30			1.44	
inhibition ratio (%)							67.03	63.06	68.97	88.27	95.14	94.04
average (%)								66.35			92.48	
SD (%)								3.01			3.69	
P value									0.0008			
		P value										
Vector vs LKB1		0.0358										
Vector vs Vector-shJOSD2		0.0012										
LKB1 vs LKB1-shJOSD2		0.0085										

Supplementary Figure 7f

		Ctrl	Ctrl+JOSD2	LKB1	LKB1+JOSD2	3KR	3KR+JOSD2
	replicate-1	1.000	0.768	0.779	2.779	1.049	0.785
	replicate-2	1.000	0.740	0.570	2.408	1.062	0.871
	replicate-3	1.000	0.927	0.549	2.548	0.992	0.894
	replicate-4	1.000	0.864	0.617	2.868	1.246	1.052
	replicate-5	1.000	0.706	0.944	8.570	0.795	1.024
	replicate-6	1.000	0.964	1.068	8.354	1.715	2.000
			P value				
Ctrl *vs.* LKB1-WT			0.0379				
Ctrl *vs.* LKB1-3KR			0.3163				
LKB1-WT *vs.* LKB1-WT+JOSD2			0.0260				

Supplementary Figure 8c

JOSD2/GAPDH	Number	replicate-1	replicate-2	replicate-3	AVRAGE
Scramble	1	0.437	0.487	0.585	0.503
	2	0.936	0.832	0.779	0.849
	3	1.000	1.000	0.795	0.932
	4	0.540	0.888	0.937	0.788
	5	0.534	0.809	1.000	0.781
shJOSD2	1	0.325	0.076	0.108	0.170
	2	0.103	0.098	0.112	0.104
	3	0.205	0.090	0.144	0.146
	4	0.231	0.103	0.130	0.155
	5	0.220	0.108	0.186	0.171
P value	Scramble vs shJOSD2				
	0.0008				

p-LKB1/GAPDH	Number	replicate-1	replicate-2	replicate-3	AVRAGE
Scramble	1	1.011	3.077	1.000	1.696
	2	1.186	4.508	1.363	2.352
	3	1.000	3.740	1.001	1.914
	4	2.023	1.489	1.390	1.634
	5	2.288	1.000	1.328	1.538
shJOSD2	1	2.355	10.346	6.526	6.409
	2	3.927	10.015	8.548	7.497
	3	4.224	9.297	7.699	7.073
	4	4.177	13.103	6.085	7.788
	5	3.689	14.033	5.786	7.836
P value	Scramble vs shJOSD2				
	1.3E-06				

Supplementary Figure 8e

Cell Cycle									
	%	control	shJOSD2				control	shJOSD2	*P* value
Repeat-1	G1	59.6	76.4		G1	Repeat-1	59.6	76.4	0.0090
	S	25	13.1			Repeat-2	47.8	71.3	
	G2	11.2	8.5			Repeat-3	51	84.2	
		control	shJOSD2						
Repeat-2	G1	47.8	71.3		S	Repeat-1	25	13.1	0.0360
	S	38.1	21.8			Repeat-2	38.1	21.8	
	G2	12.6	6.15			Repeat-3	38.6	13.4	
		control	shJOSD2						
Repeat-3	G1	51	84.2		G2	Repeat-1	11.2	8.5	0.0583
	S	38.6	13.4			Repeat-2	12.6	6.15	
	G2	9.69	3.09			Repeat-3	9.69	3.09	

Supplementary Figure 9f

	Replicated-1		Replicated-2		Replicated-3		
	control	shJOSD1	control	shJOSD1	control	shJOSD1	
Day 1	1.00	1.00	1.00	1.00	1.00	1.00	
Day 2	1.54	1.53	1.56	1.57	1.49	1.57	
Day 3	2.48	2.27	2.35	2.08	2.48	2.26	
Day 4	3.53	2.99	3.28	3.28	3.60	3.45	
Day 5	4.99	4.24	4.23	4.01	5.34	4.58	
			Day 5				
		Replicated-1	Replicated-2	Replicated-3		*P* value	
	control	4.99	4.23	5.34			
	shJOSD1	4.24	4.01	4.58		control vs shJOSD1	0.2141

Supplementary Figure 10a

	Survival Fraction (%)								Survival Fraction (%)								
concentration (μM)	NCI-H460			A549				concentration (μM)	NCI-H23			NCI-H838			NCI-H1395		
0.3	26.22	20.97	29.31	80.00	78.56	76.59		1	12.37	11.62	12.68	10.25	8.23	10.52	7.67	5.76	7.18
0.25	43.24	48.33	49.54	89.19	82.36	78.96		0.75	49.93	44.37	51.97	63.01	57.19	45.16	27.26	23.21	31.35
0.2	61.9	80.35	65.75	105.69	89.59	93.82		0.5	83.93	84.71	89.56	86.18	77.66	77.19	52.52	54.16	58.78
0.15	81.11	94.41	87.75	105.91	90.02	99.59		0.375	88.25	98.72	104.35	89.59	81.55	71.49	46.59	50.97	40.32
0.1	86.11	108.25	94.88	112.64	97.86	106.08		0.25	98.34	99.73	107.32	87.56	107.78	120.60	101.80	86.71	88.39
						LKB1-null	IC_{50} (μM)	LKB1-WT	IC_{50} (μM)								
						NCI-H1395	0.46	NCI-H292	0.14								
						NCI-H23	0.73	NCI-H358	0.13								
						A549	0.46	NCI-H1299	0.1								
						NCI-H460	0.24	PC-9	0.12								
						NCI-H838	0.72										
						Average	0.52		0.1225								
						P value		0.0119									

Supplementary Figure 10b

HY041004	Vector Inhibiton ratio			LKB1 Inhibiton ratio			3KR Inhibiton ratio		
0 µM	100.00	100.00	100.00	100.00	100.00	100.00	100.00	100.00	100.00
0.15µM	104.34	96.57	104.21	100.36	96.42	102.42	105.48	95.87	102.74
0.2µM	89.71	91.82	97.90	82.72	85.24	88.95	93.05	89.08	95.76
0.25µM	83.19	85.37	91.68	66.53	68.34	71.09	79.18	80.68	86.82
0.3µM	63.51	65.70	71.64	42.37	37.63	54.46	60.72	63.25	66.41
		P value			*P* value			*P* value	
Vector vs LKB1	0 µM		Vector vs 3KR	0 µM					
	0.15µM	0.5650		0.15µM	0.9326				
	0.2µM	0.0752		0.2µM	0.8777				
	0.25µM	0.0080		0.25µM	0.2616				
	0.3µM	0.0305		0.3µM	0.3081				

Supplementary Figure 11

	Ctrl			LKB1			LKB1+JOSD2			Ctrl+cal101			LKB1+cal101			LKB1+JOSD2+cal101		
Day 1	1	1	1	1	1	1	1	1	1	1	1	1	1	1	1	1	1	1
Day 2	1.572	1.631	1.628	1.323	1.361	1.390	1.447	1.673	1.657	1.282	1.381	1.340	1.160	1.200	1.296	1.427	1.431	1.435
Day 3	2.982	3.180	3.081	2.699	2.918	2.929	3.583	3.539	3.543	1.892	1.796	1.701	1.606	1.493	1.683	1.899	2.033	2.186
Day 4	6.004	6.453	6.173	5.091	5.759	5.521	7.230	7.570	7.423	2.386	2.504	2.253	2.014	2.076	1.945	2.509	2.485	2.550
Average	6.210			5.457			7.408			2.381			2.012			2.515		
P value					0.0399			0.0025						0.0202			0.2015	
				LKB1 vs Ctrl			LKB1+JOSD2 vs Ctrl						LKB1+cal101 vs Ctrl+cal101			LKB1+JOSD2+cal101 vs Ctrl+cal101		
